# Listen up, kids! How mind wandering affects immediate and delayed memory in children

**DOI:** 10.3758/s13421-023-01509-0

**Published:** 2023-12-27

**Authors:** Jessica Cherry, Teresa McCormack, Agnieszka J. Graham

**Affiliations:** School of Psychology, Queen’s University Belfast, Belfast, BT7 1NN UK

**Keywords:** Mind wandering, Task-unrelated thoughts, Cognitive development, Learning, Memory

## Abstract

Mind wandering occurs when attention becomes disengaged from the here-and-now and directed toward internally generated thoughts; this is often associated with poorer performance on educationally significant tasks. In this study, 8- to 9-year-old children (*N* = 60) listened to audio stories embedded with intermittent thought probes that were used to determine if participants’ thoughts were on or off task. The key objective was to explore the impact of probe-caught mind wandering on both immediate and delayed memory retention. Children reported being off task approximately 24% of the time. Most inattention episodes were classified as task-unrelated thoughts (i.e., ‘pure’ instances of mind wandering, 9%) or attentional failures due to distractions (9%). Higher frequency of mind wandering was strongly associated with poorer memory recall, and task-unrelated thoughts strongly predicted how well children could recall components of the audio story both immediately after the task and after a 1-week delay. This study is the first to demonstrate the impact of mind wandering on delayed memory retention in children. Results suggest that exploring mind wandering in the foundational years of schooling could provide the necessary empirical foundation for the development of practical interventions geared toward detecting and refocusing lapses of attention in educational contexts.

**Supplementary Information:**

The online version contains supplementary material available at 10.3758/s13421-023-01509-0.

People’s conscious experiences are not always tied to ongoing events and immediate surroundings. For example, while reading this article, you may suddenly realize that you are no longer paying attention to the text and that, in spite of your best efforts to maintain focus, you start thinking about what you are going to eat this evening. This shift of attention from the task at hand towards internally generated thoughts is often referred to as *mind wandering* (Murray et al., 2020; Smallwood & Schooler, 2006). Although specific definitions vary, in psychological research, mind wandering is typically operationalized as task-unrelated thought (Murray & Krasich, 2022). Studies of daily-life mind wandering indicate that it consumes a substantial amount of time (Seli et al., 2018) and can be costly in educational contexts because learning depends on extracting information from the learning environment and aligning this new information with existing knowledge (e.g., Sanchez & Naylor, 2018); mind wandering signals a breakdown in this process. To date, the link between mind wandering and learning has been studied primarily in adult student populations, generating a body of evidence in authentic settings of potential value to educators interested in its impact on educational outcomes (Szpunar, 2017).

Strikingly, despite its obvious educational significance, only a relatively small number of studies have attempted to examine mind wandering in children (Cherry et al., 2022; Frick et al., 2020; Jones, 2019; Keulers & Jonkman, 2019; McCormack et al., 2019; Van den Driessche et al., 2017; Wilson et al., 2022; Ye et al., 2014; Zhang et al., 2015). The findings of these studies are promising, though, in that they suggest that it is possible to take meaningful measures of inattentive episodes in children as young as around 7 years. Nevertheless, to date, little is known about the impact of mind wandering on children’s learning. Although it may seem probable, based on findings with adults, that mind wandering is detrimental to children’s learning, it is important to establish this empirically, in particular because it cannot be taken as a given that children’s reports of mind wandering are sufficiently robust as to be predictive of educationally significant outcomes.

Much work in the field of mind wandering has focused on understanding and quantifying the costs of mind wandering in learning environments, with most of the educationally relevant research in adults and adolescents suggesting that higher rates of mind wandering are associated with poorer comprehension and less learning. In studies of mind wandering while reading, participants read texts and periodically classify the focus of their thoughts as ‘on task’ or ‘off task’ (e.g., asked to judge whether they are thinking about *The text*; *How well I’m understanding the story*; *A memory from the past*; *Something in the future*; *Current state of being*; *Other*; McVay & Kane, 2012; Unsworth & McMillan, 2013) in response to structured probes, with certain off-task responses (e.g., *A memory from the past*; *Something in the future*) treated as a measure of mind wandering. These types of studies reliably show that participants who report more task-unrelated thoughts in response to probes during reading also tend to recall less of what they read than those who report fewer task-unrelated thoughts (McVay & Kane, 2012; Sanchez & Naylor, 2018; Smallwood et al., 2008; Unsworth & McMillan, 2013). Furthermore, mind-wandering rates are relatively stable in that those who mind wander more during one reading task also tend to mind wander more in others (Al-Balushi & Al-Harthy, 2015; McVay & Kane, 2012).

A complementary strand of evidence for the link between mind wandering and learning comes from studies embedding intermittent thought-probes within live or video-recorded lectures and other types of learning activities (e.g., discussions, problem-solving tasks, student presentations; Bunce et al., 2010; Cameron & Giuntoli, 1972; Locke & Jensen, 1974; Risko et al., 2012; Schoen, 1970; Shukor, 2005; Szpunar et al., 2013). Despite considerable variation in lecture durations, playback speed, and topics, adult participants consistently report mind wandering approximately 30%–40% of the time and, in line with the reading comprehension findings noted earlier, students who mind wander more tend to recall less of the lecture content (Hollis & Was, 2016; Kane et al., 2017; Murphy et al., 2023; Wammes et al., 2016; Wammes & Smilek, 2017). Higher rates of mind wandering have also been associated with taking fewer and poorer quality notes during lectures (Dewey, 2020; Jing et al., 2016; Kane et al., 2017; Szpunar et al., 2013; Wong & Lim, 2021) and with lower topic interest and motivation (Lindquist & McLean, 2011; Seli et al., 2015; Wammes et al., 2016). Furthermore, many studies with young adults have indicated that the occurrence of mind wandering increases for young adults as a function of time-on-task in lectures (Farley et al., 2013; Risko et al., 2012; Varao-Sousa & Kingstone, 2019). Interestingly, a growing body of literature suggests that older adults tend to mind wander less than younger adults when completing learning activities (14% vs. 40%, Jackson & Balota, 2012; 19% vs. 42%; Murphy et al., 2023). This trend also extends to other daily activities (Jordão et al., 2019; Maillet et al., 2018; Seli et al., 2017).

Although a handful of recent studies have attempted to look at mind wandering in the childhood period before adolescence (Frick et al., 2020; Jones, 2019; Keulers & Jonkman, 2019; McCormack et al., 2019; Van den Driessche et al., 2017; Wilson et al., 2022; Ye et al., 2014; Zhang et al., 2015) only Cherry et al. (2022) specifically investigated the link between off-task thinking and learning in children. In their study, 6- to 11-year-olds listened to an experimenter read out a story. Intermittently during the listening exercise, children were probed by a computer to report whether their thoughts were focused on the story they were listening to or whether they were thinking about something else. Immediately after the story ended, children completed a multiple-choice memory test based on the story material and indicated their situational interest in the story topic. Cherry et al. (2022) reported three key findings. First, children who reported more off-task episodes also showed poorer immediate recall for the information that they had just been exposed to. Second, children self-reported being off task around 25% of the time, a figure that did not change significantly with age, suggesting there may not be age differences in levels of off-task thoughts in school-aged children. The third finding was that situational interest had a significant indirect effect on memory recall via off-task thoughts.

While Cherry et al.’s (2022) findings indicate that off-task thoughts can be detrimental for children’s ability to recall information after a short delay, there is much that is still not known about the link between children’s mind wandering and their learning. Here, our aim was to begin to fill these gaps in existing knowledge, focusing on three specific issues. First, we sought to provide a more detailed analysis of children’s inattentive episodes by distinguishing between task-unrelated thoughts, task-related interference, and attentional lapses rooted in distractions. Our second objective was to assess the stability of mind wandering reports across two similar testing sessions. The third and final aim of the present study was to explore the impact of probe-caught mind wandering on delayed, rather than just immediate, memory recall. We now go on to describe the motivation behind each of these objectives.

## Current study aims

Although Cherry et al. (2022) found a relation between off-task thoughts and children’s immediate memory recall, their measure of mind wandering did not distinguish between internally generated task-unrelated thoughts (i.e., ‘pure’ instances of mind wandering) and other forms of inattention. Mind wandering is typically characterized as involving a shift away from processing events in the external environment and towards self-generated thoughts; this perceptual decoupling makes mind wandering conceptually distinct from other forms of inattention, such as task-related interference or external distractions (Barron et al., 2011; Smallwood, 2013). Thus, thought-probe procedures that only distinguish between on-task and off-task thoughts might not provide a true estimate of ‘pure’ mind wandering, which may lead to erroneous inferences. Indeed, in studies with adults, participants not only routinely report mind wandering during lectures and while studying, they also frequently report being distracted by information in the external environment. A diary study where college students were asked to report on their attentional failures over the course of a week found that 31% of the reported attentional failures were due to distractions either while studying (22%) or while in class (9%; Unsworth, Brewer et al. 2012a, Unsworth, McMillan et al. 2012b). Like mind wandering, external distraction reflects a general lapse of attention and accordingly can also impede performance, learning, and memory (Stawarczyk et al., 2011; Varao-Sousa et al., 2018). For example, Shelton et al. (2009) found that hearing a ringing mobile phone during a lecture resulted in lower retention for material presented at the same time as the ringing mobile phone, compared with material presented before the onset of the ringtone. In such situations attention is shifted from the current task to irrelevant (and potentially irritating) information in the external environment. External distractors can take on multiple forms including extraneous noises and sights (e.g., talking, other students moving around), or bodily sensations (e.g., feeling too hot or too cold, feeling hungry).

To provide a more accurate characterization of children’s inattentive episodes during learning activities, in the current study, child participants were asked to make a distinction between task-unrelated thoughts, task-related interference, and instances of inattention rooted in distraction during educational-style stories about historical events. In line with the extant literature in adults, task-unrelated thoughts were operationalized as thoughts that are unrelated to the ongoing, externally oriented, task (e.g., reminiscing about past events, contemplating the future, fantasizing). By contrast, task-related interference was defined as episodes involving evaluative thoughts about the task or about task performance (e.g., *I’m not very good at this, I don’t find this interesting*; e.g., Sarason et al., 1986; Smallwood et al., 2004). In the area of mind wandering, reports of task-related interference are typically excluded from data analysis because, as noted by McVay and Kane (2009), task-related thoughts are an “ambiguous intermediary between on- and off-task thought” (p. 200) and thus subject to ambiguous interpretations (McVay & Kane, 2009, 2012; Unsworth & McMillan, 2013).

To the best of our knowledge, Van den Driessche et al.’s (2017) study of children with and without attention-deficit/hyperactivity disorder (ADHD) is the first to date that has attempted to get children to subcategorize their off-task thoughts into task-unrelated thoughts, task-related interference, and instances of inattention rooted in distraction while completing a series of go/no-go trials. Their data suggest that children may be able to use these categories, although the age of their sample of children was wide (20 typically developing 6–12-year-olds). Building on their promising initial findings, in the present study we also asked children to further categorize their off-task thoughts, and then examined whether the link between mind wandering and learning reported by Cherry et al. (2022) remains intact when the index of mind wandering reflects only the frequency of task-unrelated thoughts. We also examined whether task-related interference and distraction were predictive of learning. Distinguishing between different categories of inattention is important because it is essential for informing how best to develop child-friendly strategies to enhance task-focused behavior during learning activities. Thus, our study examined whether children, like adults, can make such distinctions, and whether episodes in these different categories were differentially predictive of learning.

The second aim of the current study was to assess the stability of mind wandering reports. As already noted, adults’ mind wandering reports are relatively consistent in the sense that those who mind wander more during a particular task also tend to mind wander more in other similar tasks (Al-Balushi & Al-Harthy, 2015; McVay & Kane, 2012; Varao-Sousa & Kingstone, 2019). Although it is promising that the handful of studies that have used the probe-caught method with children provide comparable estimates of the frequency of off-task thoughts (25%, Cherry et al., 2022; 20-25%, Keulers & Jonkman, 2019; 33%, Zhang et al., 2015), to the best of our knowledge only Keulers and Jonkman’s (2019) study looked at the relation between the frequency of children’s off-task thoughts reported in one session and that reported in another. These authors found that, in 9- to 11-year-olds, levels of self-reported off-task thoughts were moderately correlated across the two sessions, despite the fact that the tasks completed in each session were quite different (i.e., a listening task versus a battery of executive function tasks). We sought to replicate this finding when distinguishing between different categories of off-task thought rather than just general inattentiveness, and also to examine whether the correlation may be stronger if the primary task was similar for the two testing sessions (listening to a story).

Our third key objective was to replicate and extend the results of Cherry et al. (2022) by measuring the impact of probe-caught mind wandering on not only immediate memory recall, but also delayed memory recall to test whether the effect is still present after a 1-week delay. In the adult literature, the longer-term impact of mind wandering on academic performance has been most often studied in the context of exam performance or end of term/year course marks (Kane et al., 2021; Mrazek et al., 2012; Wammes et al., 2016). The handful of studies that have included a delayed memory measure typically sought to demonstrate the effectiveness of different strategies at reducing mind wandering and, ultimately, improving academic success (e.g., Fenesi et al., 2018; Mills et al., 2021; Peterson & Wissman, 2020). Taken together, these studies clearly suggest that mind wandering is a useful predictor of delayed learning performance. However, it is still unknown whether self-reports of mind wandering in child populations is similarly predictive of long-term learning. Nevertheless, based on the adult findings, we anticipated that our mind wandering measure would predict delayed as well as immediate memory performance. Moreover, we also sought to examine the relation between level of interest in the listening task, mind wandering, and both immediate and delayed memory performance. Cherry et al. found that task interest had a significant indirect effect on memory performance via off-task thoughts, suggesting that participants with lower interest in the story topic were potentially more likely to engage in mind wandering with detrimental effects on their memory for the story contents. We hoped to replicate this finding in our study and extend it to include delayed as well as immediate recall.

## The present study

To achieve the objectives listed above, each participant completed two testing sessions scheduled approximately one week apart. On both occasions, 8- to 9-year-old children listened to an audio story (one about a fictional Pharaoh based in ancient Egypt) at Time 1 [T1] and a different story (about a fictional species of dinosaurs based in the Cretaceous period) at Time 2 [T2] and reported if their thoughts were on or off task in response to child-friendly structured probes. If children reported being off task they were further instructed to categorize their thought as task-related (i.e., episodes of task-related interference), task-unrelated, or a result of distraction, with task-unrelated episodes conceptualized as instances of ‘pure’ mind wandering. Participants also completed two immediate memory tests (one after each story) and one delayed memory test probing their ability to recall key components of the story they had listened to 7 days prior. We also measured participants’ verbal ability and collected ratings of prior and situational interest in story topics.

Several predictions were formulated for the present research. We expected that children would report being off task approximately 20%–33% of the time, in line with previously published reports (Cherry et al., 2022; Keulers & Jonkman, 2019; Zhang et al., 2015), and that there would be a correlation between reported levels of off-task thoughts across T1 and T2. However, our primary purpose was not simply to establish rates of mind wandering in children, which might vary by context, but to examine the relative frequency of different types of episodes of inattention. Based on previous studies with adult participants conducted in educational environments, we expected task-unrelated thoughts and attentional lapses due to distraction to be more frequent than task-related interference (Kane et al., 2017, 2021; Was et al., 2019). We anticipated that, using our measure of ‘pure’ mind wandering, we would find that higher levels of mind wandering during a learning activity are predictive of poorer immediate memory recall, consistent with the findings of Cherry et al. (2022). Furthermore, based on research with adult populations (e.g., Fenesi et al., 2018; Wammes et al., 2016) we hypothesized that the impact of mind wandering on memory recall would still be present after a weeklong delay. We also predicted that mind wandering would mediate the relationship between memory retention and ratings of interest in the topic of the story, replicating Cherry et al. (2022) among others (Hollis & Was, 2016; Soemer et al., 2019; Unsworth & McMillan, 2013).

## Method

### Participants

The total sample included 61 8- to 9-years-olds (50.82% female, *M*_age_ = 8.99 years, *SD*_age_ = 0.52). A power analysis conducted using the ‘pwr’ package in R (Champely, 2012) indicated that this sample size was sufficient to detect medium linear regression effects at 80% power and α = .05. One participant was unable to attend the second testing session and their data were removed from the analyses. The final sample consisted of 60 children aged between 8–9 years (50% female, *M*_age_ = 8.99 years, *SD*_age_ = 0.52). Due to local demographics, the majority of participating children were white (98.33%) and of low to middle socioeconomic status.

All participants were recruited through parental interest generated by advertisements placed on social media platforms. Due to ongoing disruptions to face-to-face data collection caused by the COVID-19 pandemic, children were tested online using Microsoft Teams video-conferencing software. Over both testing sessions, most parents chose to stay in the same room as their children. Average scores obtained on the measure of verbal ability (Wechsler, 2014) indicated that participating children were just above the expected range (*M* = 10.92, *SD* = 2.66, where 10 is the standardized average score).

### Materials and procedure

Data collection took place over video-conferencing software via a series of PowerPoint presentations across two separate testing sessions approximately one week apart (*M* = 6.93 days, *SD* = 1.91). The first testing session (T1) began with the researcher providing an age-appropriate overview of the study. Children then took part in an extensive training procedure; a cartoon character was introduced to explain the distinctions between different categories of thoughts (on-task, task-related interference, task-unrelated, and external distractions; see supplementary materials for additional details).

The children then engaged in a sorting activity to organize a sample of off-task thoughts into one of three boxes representing task-related interference (‘thoughts connected to the story’), task-unrelated thoughts (‘thoughts about other things happening at different times’), and attentional failures due to external distractions (‘thoughts about other things happening right now’). In the current study, 23.33% (14 children) made one or more errors in the initial sorting task, with the remaining 76.67% (46 children) successfully sorting the different off-task thoughts on their first try. Next, each child completed four practice trials. During practice, children listened to brief descriptions of the cartoon character’s thoughts before responding to thought probes. To answer each of the four probes, participants first made judgments on whether the cartoon character’s thoughts were on task (‘thinking about what was just said in the story’) or off task (‘thinking about something different’). If the cartoon character was thinking about things other than what was just said in the story, the children had to categorize the thought as task-unrelated, task-related interference or as an episode of inattention caused by external distraction. If the first four practice trials were completed successfully, children advanced to the listening activity. Otherwise, an additional four practice trials would commence. In the first set of training trials, the majority of participants (93.33%, *n* = 56) were able to correctly identify all four of the fictional character’s thoughts as on or off task, and when off task, they were accurately able to categorize the thoughts as task-unrelated thoughts, task-related interference or thoughts rooted in distraction. The remaining 6.67% (four children) made an error when sorting the fictional character’s thoughts in the first phase of practice questions but then went on to correctly answer the second set of practice questions. Error-free completion of at least one set of training trials was a prerequisite for taking part in the study. The precise wording of all task procedures can be found in the supplementary materials.

It was then explained to the children that they were going to listen to a story and that during that story they themselves would be probed about their thoughts to which they would answer verbally. At this point, children were asked to rate their general interest in the topic area (i.e., ancient Egypt at T1 and dinosaurs at T2) using a 5-point scale, ranging from *I really don’t like it* to *I really like it*. Another 5-point scale ranging from *I really didn’t like it* to *I really liked it* was displayed at the end of each story to gauge situational interest for the content of the listening task.

**Fig. 1:**
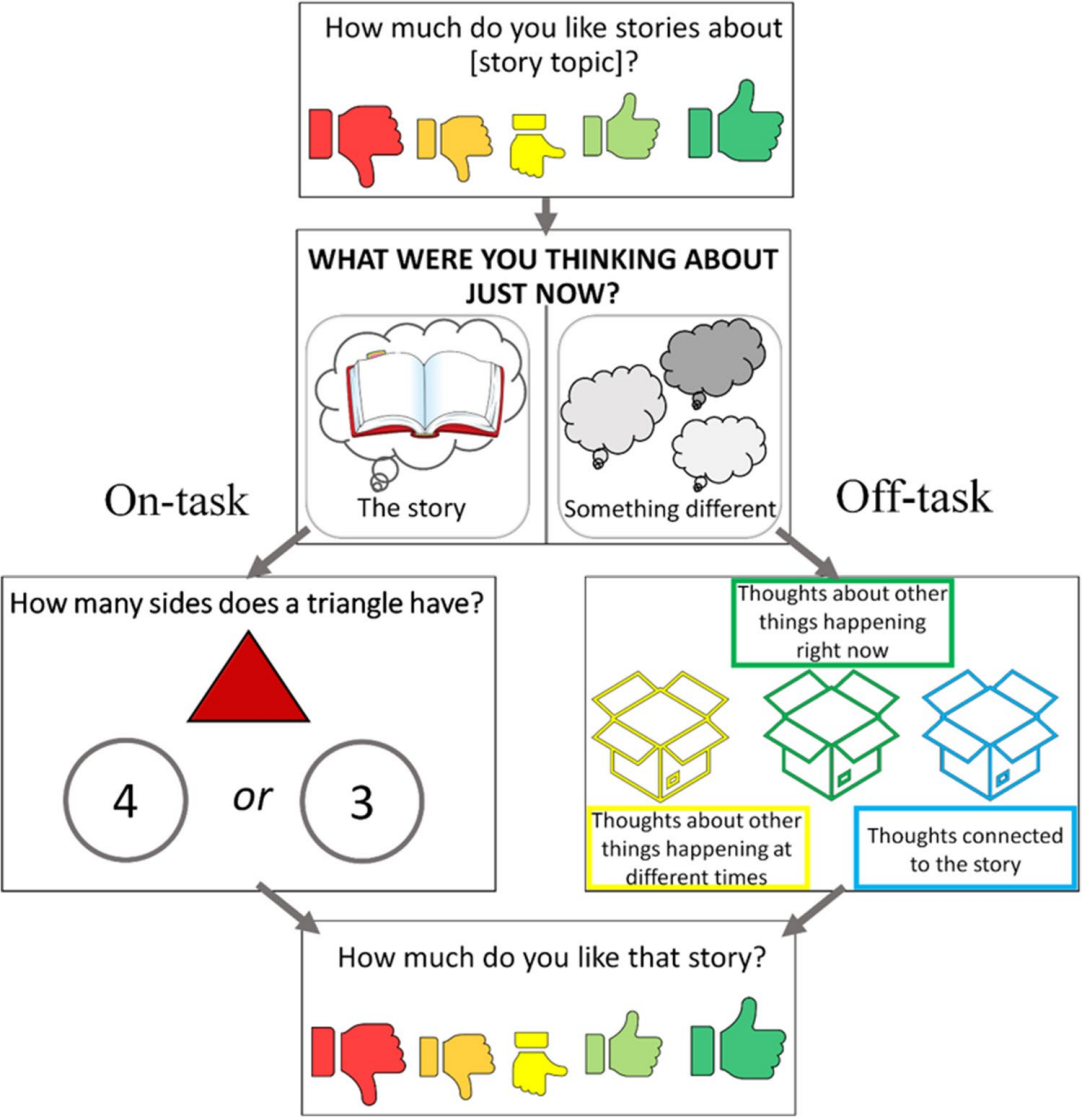
The structure of each thought probe. Participants were asked to answer verbally (i.e., *I was thinking about what was just said in the story* or *I was thinking about something different*). The sequence on the left side was presented if the child reported on-task thoughts, whereas the right side shows the pathway taken if thoughts were reported as off task. Topic interest was measured both before and after each story. (Color figure online)

Once a rating of prior interest in the story topic was obtained, the story was played, and children were probed about their thoughts. The story about ancient Egypt was 1,854 words in length and lasted just over 12 minutes, the spoken word rate of about 2.5 words per second is regarded as the average speech production rate (Tauroza & Allison, 1990). To gain reliable and valid information about individual differences in mind wandering rate, and in line with recommendations from Welhaf et al. (2022), each story was embedded with eight intermittent thought probes that appeared on the screen approximately every 85 s (with a range of 65–105 s). Each probe consisted of an initial question dichotomizing whether the participant’s thoughts were on or off task (i.e., *What were you thinking about just now? What was just said in the story or something different?*). If the participant was thinking about something different, they were asked to categorize their thought as task-unrelated, task-related interference, or driven by distraction. This was achieved by asking children to ‘place’ their thoughts into one of three boxes (i.e., *Can you tell me in which box does your thought belong? Is the thought about other things happening at different times, is the thought about other things happening right now, or is the thought connected to the story?*). When participants instead indicated they were thinking about what was just said in the story, they were asked to answer a simple factual question by selecting one of two alternatives (e.g., *How many sides does a triangle have? Three or four?*). Inclusion of this factual question ensured that number of questions asked after each probe was equal for all participants regardless of levels of on- or off-task thoughts; this follow-up question served as an attempt to make task completion time comparable across the entire sample. For a visual representation of the question layout see Fig. 1; note that all text displayed on the screen was also audio-presented to all children.

When the mind wandering task ended, participants indicated their situational interest in the story they just listened to (see Fig. 1) and completed an immediate memory retention test consisting of 10 questions in a multiple-choice format with three alternatives. All questions were derived from novel material presented within the fictional stories to ensure answers could not be based on participants’ prior knowledge on the topics. As there were two sets of 10 item memory test questions about ancient Egypt (one presented immediately after the story and another presented after a one-week delay), the tests were administered in a counterbalanced order (Set A/B at T1, followed by Set B/A at T2). All questions were scored as correct (1) or incorrect (0).

Finally, at the end of the first session, all children had their verbal ability assessed using the vocabulary subtest from the WISC-V (Wechsler, 2014). The vocabulary subtest required children to either name or define a range of items with the prompt “What is this?” or “What does . . . mean?” The first four items were picture items, the following items were all presented orally. The children could achieve a score of 0, 1, or 2 depending on the accuracy of their response.

**Fig. 2:**
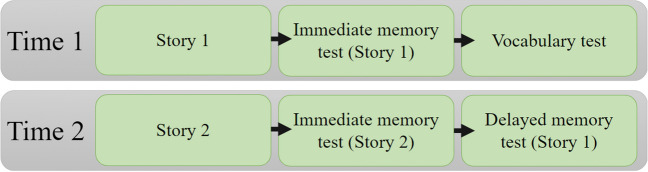
The structure of the testing sessions. At T1 participants were played the story about ancient Egypt; immediately after listening to the story they answered 10 memory questions about the story. At the end of this testing session, participants had their vocabulary ability assessed. Approximately a week later at T2, children listened to a new audio story about dinosaurs. Immediately after the children completed a memory test about that story. Finally, at the end of T2, children answered 10 memory questions about the Egypt story they had listened to at T1 to test delayed memory. As there were two sets of 10 memory test questions about ancient Egypt (i.e., 20 items total) these memory tests were presented in a counterbalanced order. Children who completed Set A at T1 went on to complete Set B at T2 and the other set of children who completed Set B at T1 then went on to complete Set A at T2

The second testing session (T2) followed a similar format. After a brief overview of the session, the children completed another thought sorting activity and four or eight training trials. Similar to T1, the majority of participants completed the first four training trials without error (93.33%, *n* = 56). Following the successful completion of training procedures, children were asked to rate their interest in dinosaurs before listening to the second audio story. The listening activity about dinosaurs had a word length of 1,517 words and lasted just over 10 minutes, again the speech rate of 2.5 words per second is regarded as normal speech rate. As was the case at T1, the audio story contained 8 intermittent thought probes. When the story had finished, the children rated how much they enjoyed it and also completed two sets of multiple-choice questions. The first set of questions tested children's ability to recall content from the story they had just listened to (i.e., the story about dinosaurs). The following 10 questions tested children's delayed memory of the information from the story played at T1 (i.e., the story about ancient Egypt). If the children were shown Set A questions about the story at T1, they completed Set B at T2; if Set B was completed at T1 they received Set A at T2. The structure of the testing sessions is outlined in Fig. 2.

The study was approved and conducted in accordance with the guidelines of the Faculty Ethics Committee of the authors’ university. Parents were fully debriefed and provided informed consent for participation prior to testing. Children also provided assent prior to and on both days of testing. All participants received a voucher worth £10 (British pounds) for completing both testing sessions.

## Results

**Fig. 3:**
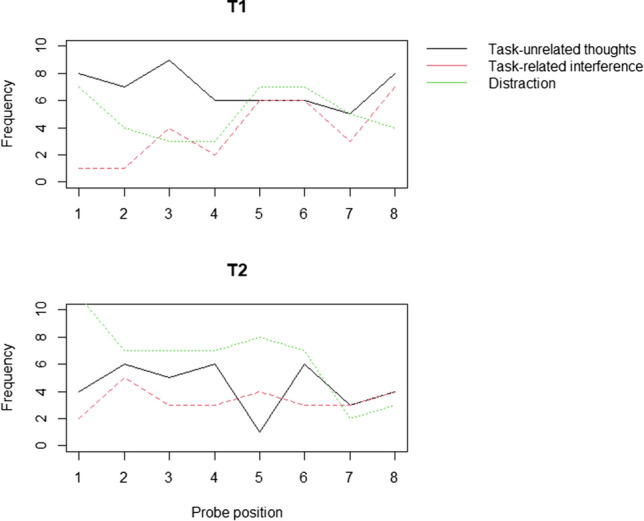
Line graphs depicting the frequency of different types of off-task thoughts across the duration of both testing sessions. (Color figure online)

Data were analyzed using R (R Core Team, 2021). A summary of task performance and normality values is provided in Table 1. Across both testing sessions children reported being off task 24.17% of the time. Summed across both sessions, task-unrelated thoughts were reported most often—9.48% of the time—while the frequency of task-related interference was 5.52%. Attentional failures rooted in external distraction accounted for 9.17% of probe-caught responses. To assess if attentional states differed significantly between the first half and the second half of the listening activities, a series of paired *t* tests were run using the ‘t,test’ function in R (visual depiction of off-task probe responses can be found in Fig. 3). At T1, there was a significant effect of time on task, *t*(59) *=* −2.27, *p* = .027 for task related interference only. Participants reported more task -related interference in the second half of the activity (8%) compared with the first half of the activity (3%). None of the other attentional states varied significantly during the first and second half of the listening activity (*p* > .05). At T2, participants reported being on-task significantly more as the story progressed from the first half (73%) to the second half (80%), *t*(59) = −2.12, *p =* .039. This was also marked by fewer thoughts about distractions in the second half (8%) compared with the first half of the activity (13%), *t*(59) = −2.26, *p =* .028. Rates of mind wandering and task-related interference did not differ significantly from the first half to the second half of the listening activity at T2 (*p* > .05).

**Table 1:** Descriptive statistics for variables of interest

	*N*	*M (SD)*	Min	Max	Skew	Kurtosis
T1 on-task (out of 8 probes)	60	6.02 (0.93)	3	8	−0.36	−1.05
T1 task-unrelated thoughts (out of 8 probes)	60	0.93 (1.07)	0	4	1.08	0.63
T1 task-related interference (out of 8 probes)	60	0.45 (0.72)	0	3	1.57	1.92
T1 distractions (out of 8 probes)	60	0.60 (0.74)	0	2	0.81	−0.71
T2 on-task (out of 8 probes)	60	6.10 (1.55)	3	8	−0.29	−0.92
T2 task-unrelated thoughts (out of 8 probes)	60	0.58 (0.77)	0	3	1.12	0.49
T2 task-related interference (out of 8 probes)	60	0.43 (0.75)	0	3	1.89	0.80
T2 distractions (out of 8 probes)	60	0.87 (0.93)	0	3	0.80	−0.29
T1 immediate test (out of 10)	60	7.23 (1.48)	4	10	0.10	−0.98
Set A	30	7.13 (1.53)	5	10	0.20	−1.34
Set B	30	7.33 (1.45)	4	10	−0.47	0.83
T2 immediate test (out of 10)	60	6.85 (1.66)	3	10	−0.10	−0.98
Delayed test (out of 10)	60	5.77 (1.32)	3	8	−0.33	−0.81
Set A	30	5.73 (1.29)	3	8	−0.51	−0.37
Set B	30	5.80 (1.38)	3	8	−0.21	−1.12
T1 prior topic interest (out of 5)	60	3.68 (0.79)	2	5	0.21	−0.69
T1 situational topic interest (out of 5)	60	4.20 (0.77)	2	5	−0.90	1.57
T2 prior topic interest (out of 5)	60	3.75 (1.02)	1	5	−0.47	<0.01
T2 situational topic interest (out of 5)	60	4.33 (0.77)	2	5	−0.89	−0.04
Raw vocabulary score (out of 54)	60	24.87 (5.03)	14	41	0.66	1.30

The minimum and maximum values shown refer to observed reports, for example, in the delayed memory recall test, the minimum score achieved was 3 and the maximum score achieved was 8, as this was a 10-item test the absolute minimum that could be achieved was 0 with the maximum being 10. Normality distributions can be inferred from skewness and kurtosis values

Overall, the index of mind wandering had good split-half reliability between T1 and T2 (Spearman–Brown coefficient = 0.88). When probe responses are split into groups (i.e., two groups of four probes from T1 and another two groups of probes from T2), the split-half reliability remains strong on all comparisons (Spearman–Brown coefficients = 0.59 to 0.75). We also found that rates of total numbers off-task thoughts were highly positively correlated, *r*(58) = 0.85, *p* < .001, across the two testing sessions: Children who reported more off-task thoughts when listening to the story about ancient Egypt were also more likely to report off-task thoughts when listening to the story about dinosaurs one week later. A correlation matrix was computed for the three categories of off-task thought (task-unrelated thought, task-related interference, and thoughts rooted in distraction) reported at T1 and T2 (Table 2). We found a moderately large significant correlation between task-unrelated thoughts reported one week apart, *r*(58) = 0.67, *p* < .001, suggesting that children who mind wandered more during the audio story played at T1 were also more likely to report task-unrelated thoughts during the audio story at T2. A similar but weaker link was observed for task-related interference, *r*(58) = 0.34,* p* = .007, indicating that evaluative thoughts about the task and about task performance may be relatively stable across similar learning activities. Finally, a moderately large significant correlation was found between the proportions of time children reported being distracted at T1 and T2, *r*(58) = 0.66, *p* < .001. For each type of off-task thought, the largest cross-session correlations were consistently within each subcategory.

**Table 2:** Pearson’s correlation coefficients between the different categories of off-task thoughts reported at T1 and T2

		T1			T2	
		TUT	TRI	Distraction	TUT	TRI
T1	TRI	0.08				
	Distraction	0.09	−0.04			
	TUT	0.67**	0.34*	0.18		
T2	TRI	0.12	0.48**	0.01	0.17	
	Distraction	0.42**	−0.09	0.66**	0.21	−0.14

TUT = task-unrelated thought; TRI = task-related interference, *p < .05, **p < .001

With regards to memory recall, the two sets of 10 items testing knowledge of the ancient Egypt story had a Spearman–Brown coefficient of 0.48, and the 10 items on the dinosaur story test had a Spearman–Brown coefficient of 0.49.

### Relations with memory performance

A partial correlation matrix was constructed to explore the associations between the different types of on- and off-task thoughts and memory performance (Table 3), controlling for age (in months), topic interest, and raw vocabulary score. The overall proportion of off-task thoughts was significantly negatively correlated with both immediate and delayed recall. When off-task thoughts were further categorized, only mind wandering (i.e., the frequency of thoughts categorized as task-unrelated) was consistently negatively associated with both immediate and delayed memory performance. The rate at which children reported experiencing distractions was negatively associated with immediate memory recall only at T2 and frequency of task-related interference only at T1.

**Table 3:** Pearson’s partial correlation coefficients between the proportions of reported off-task thoughts and memory performance (controlling for topic interest ratings, age, and raw vocabulary scores)

	Immediate memory recall		Delayed memory recall
	T1	T2	
Off-task thoughts (overall)	–0.53**	–0.57**	–0.31*
Task-unrelated thoughts	–0.41*	–0.48**	–0.27*
Task-related interference	–0.32*	–0.18	–0.18
Distractions	–0.21	–0.42*	–0.12

*p < .05, **p < .001

**Fig. 4:**
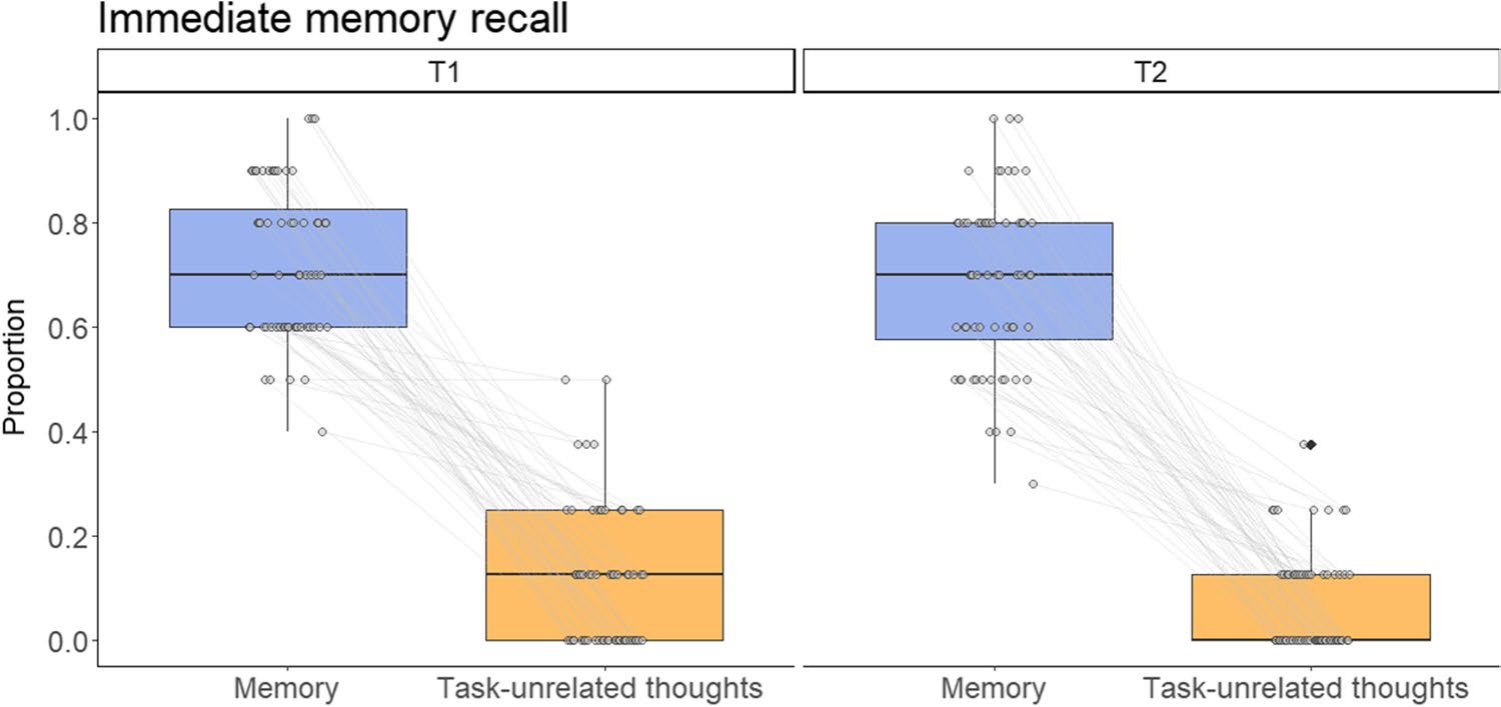
Paired-points graph demonstrating the links between task-unrelated thoughts (proportional score out of 8 probes) and immediate memory recall (proportional score out of 10 questions). Black lines indicate median; the lower and upper hinges correspond to the first and third quartiles; whiskers depict maximum and minimum values within 1.5 times the interquartile range

**Fig. 5:**
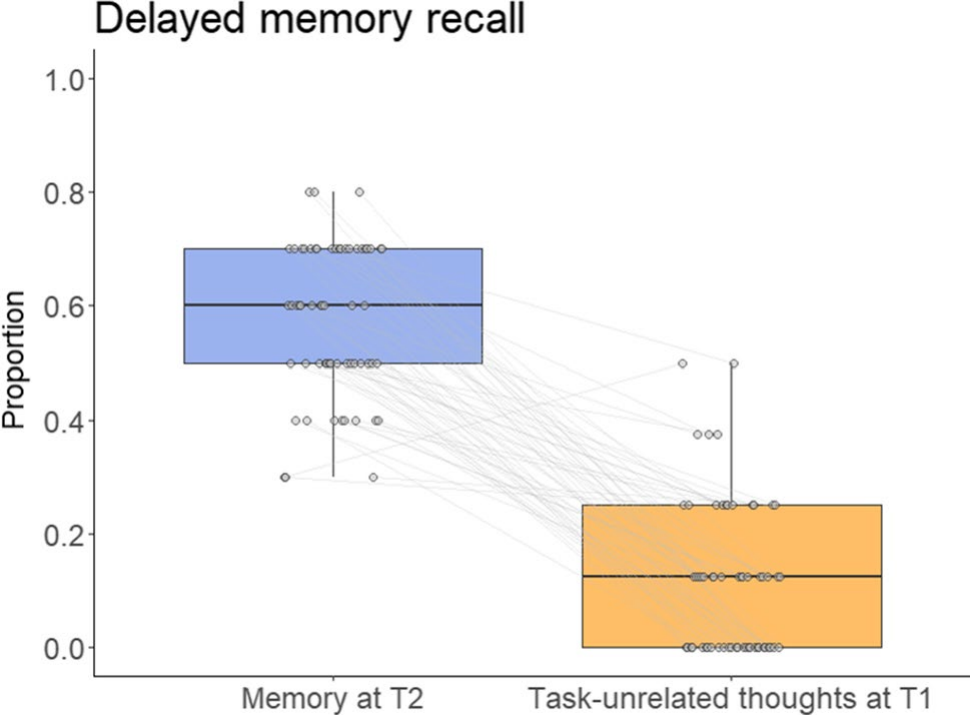
Paired-points graph illustrating the link between task-unrelated thoughts reported at T1 (proportional score out of 8) and delayed memory recall measured at T2 (proportional score out of 10). Black lines indicate median; the lower and upper hinges correspond to the first and third quartiles; whiskers depict maximum and minimum values within 1.5 times the interquartile range

The links between task-unrelated thoughts and immediate memory recall are depicted in the paired-point graph in Fig. 4, and the link between task-unrelated thoughts and delayed memory retention is displayed in Fig. 5 (note that the graphs depict the proportional values of task-unrelated thoughts and memory test performance rather than raw scores).

Prior to regression analyses, proportional data were arcsine transformed to stabilize variance and meet the necessary assumptions required for linear models. To investigate the impact of off-task thoughts on immediate memory performance we pooled the relevant data obtained at T1 and T2 before conducting multiple linear regression analyses (Table 4). To account for each participant having two data points (i.e., performance at T1 and performance at T2), participant ID was added as a random intercept in this analysis which was performed using the ‘lm’ function, alongside the ‘lm.beta’ package, in R (Behrendt, 2022). First, age and raw vocabulary score were added to the null model which accounted for a moderate amount of variance in immediate memory performance (*R*^2^_adjusted_ = .10, *F*[2, 117]= 7.37, *p* = .001). At this step, only vocabulary ability was identified as a significant predictor (β = 0.34, *p* < .001). Next, different forms of inattention—task-unrelated thoughts, distractions, and task-related interference—were added successively to the null model (see Models 1–3 in Table 4). In the final model (Model 3), task-related interference (β = −0.24, *p* = .003), thoughts due to distraction (β = −0.32, *p* < .001), task-unrelated thoughts (β = −0.29, *p* < .001), and vocabulary ability (β = 0.40, *p* < .001) were all identified as significant predictors of immediate memory recall. Model comparison using the ‘anova’ function in R, revealed that the final model provided the best fit for the data, *F*(4, 114) = 8.90, *p* = .003. The results indicate that all forms of inattention are detrimental to immediate memory performance.

**Table 4:** Linear regression analyses investigating the link between off-task thoughts and immediate memory retention (both measured at T1 and T2)

Immediate memory recall					
	*B* (*SE*)	*β*	95% CI	*R*^2^adjusted	∆*R*^2^
Null model (Intercept)	4.65 (2.38)		[−0.06, 9.36]	.10**	
Age	−0.01 (0.02)	−0.01	[−0.05, 0.04]		
Vocabulary	0.11 (0.03)	0.34**	[0.05, 0.16]		
Model 1 (Intercept)	4.41 (2.18)		[0.10, 8.72]	.24**	.14**
Age	0.01 (0.02)	0.03	[−0.03, 0.05]		
Vocabulary	0.10 (0.03)	0.31**	[0.05, 0.15]		
Task-unrelated thoughts	−2.58 (0.53)	−0.39**	[−3.64, −1.53]		
Model 2 (Intercept)	2.87 (2.13)		[−1.35, 7.08]	.31**	.07**
Age	0.02 (0.02)	0.09	[−0.02, 0.06]		
Vocabulary	0.10 (0.02)	0.32**	[0.05, 0.15]		
Task-unrelated thoughts	−2.22 (0.52)	−0.34**	[−3.25, −1.19]		
Distractions	−1.94 (0.56)	−0.28*	[−3.06, −0.83]		
Model 3 (Intercept)	1.41 (2.12)		[−2.79, 5.60]	.35**	.04**
Age	0.03 (0.02)	0.13	[−0.01, 0.07]		
Vocabulary	0.13 (0.03)	0.40**	[0.08, 0.18]		
Task-unrelated thoughts	−1.95 (0.51)	−0.29**	[−2.96, −0.94]		
Distractions	−2.23 (0.55)	−0.32**	[−3.33, −1.36]		
Task-related interference	−1.89 (0.63)	−0.24*	[−3.15, −0.63]		

*p < .05, **p < .001

Another key aim of this study was to explore the impact of off-task thoughts, particularly instances of ‘pure’ mind wandering, on delayed memory performance; to this end, another multiple linear regression analysis was conducted (Table 5). Age and raw vocabulary score were entered into the regression analysis to formulate the null model which did not significantly explain variance in delayed memory performance (*R*^2^_adjusted_ = .05, *F*[2, 57] = 2.66, *p =* .078), although vocabulary ability was identified as a significant predictor (β = 0.30, *p* = .027). Similar to the analyses described in the previous paragraph, task-unrelated thoughts, distractions, and task-related interference were added to the null model one by one to assess the contribution of different forms of inattention to delayed memory performance (see Models 1–3 in Table 5). Overall, Model 2 (containing age, vocabulary score, and task-unrelated thoughts) was deemed to have the best fit, *F*(2, 56) = 5.90, *p* = .018. Neither thoughts due to distraction nor task-related interference emerged as significant predictors of delayed memory recall.

**Table 5:** Linear regression analyses investigating the link between off-task thoughts (measured at T1) and delayed memory retention (measured at T2)

Delayed memory recall					
	*B* (*SE*)	*β*	95% CI	*R*^2^adjusted	∆*R*^2^
Null model (Intercept)	4.37 (2.89)		[−1.41, 10.15]	.05	
Age	−0.01 (0.03)	−0.02	[−0.06, 0.05]		
Vocabulary	0.07 (0.03)	0.30*	[0.01, 0.15]		
Model 1 (Intercept)	4.35 (2.77)		[−1.20, 9.90]	.13*	.08
Age	−0.01 (0.03)	−0.01	[−0.05, 0.05]		
Vocabulary	0.07 (0.03)	0.28*	[0.01, 0.14]		
Task-unrelated thoughts	−1.53 (0.64)	−0.29*	[−2.80, −0.25]		
Model 2 (Intercept)	3.77 (2.84)		[−1.93, 9.47]	.12*	-.01
Age	0.01 (0.03)	0.02	[−0.05, 0.06]		
Vocabulary	0.08 (0.03)	0.30*	[0.01, 0.15]		
Task-unrelated thoughts	−1.41 (0.65)	−0.27*	[−2.71, −0.12]		
Distractions	−0.74 (0.80)	−0.12	[−2.34, 0.85]		
Model 3 (Intercept)	2.94 (2.86)		[−2.79, 8.68]	.15*	.03
Age	0.01 (0.03)	0.05	[−0.04, 0.07]		
Vocabulary	0.09 (0.03)	0.35*	[0.02, 0.16]		
Task-unrelated thoughts	−1.32 (0.64)	−0.25*	[−2.61, −0.03]		
Distractions	−0.91 (0.79)	−0.15	[−2.50, 0.69]		
Task-related interference	−1.27 (0.82)	−0.20	[−2.92, 0.38]		

*p < .05, **p < .001

### Relations with topic interest

Topic interest ratings obtained before and after the story (i.e., prior topic interest and situational topic interest, respectively) were moderately strongly positively correlated, *r*(118) = 0.52, *p* < .001. Next, a correlation matrix was computed to assess the relationship between topic interest, mind wandering, and memory performance (Table 6). For all measures bar delayed memory accuracy, data obtained at both T1 and T2 were pooled together prior to analysis. As shown in Table 6, situational topic interest ratings (i.e., ratings gathered *after* children listened to the stories) appear to be more closely linked to indices of inattention and memory performance. Overall, however, the robust associations between topic interest, mind wandering, and memory accuracy observed in previous studies do not emerge consistently in the present experiment.

**Table 6:** Pearson’s correlation coefficients between ratings of topic interest, proportions of reported off-task thoughts and memory performance

	Prior topic interest	Situational topic interest
Immediate memory	0.17	0.14
Delayed memory	0.10	0.32*
Off-task thoughts	−0.08	−0.21*
Task-unrelated thoughts	−0.08	−0.15
Task-related interference	−0.01	−0.01
Distractions	−0.05	−0.19*

*p < .05, **p < .001

To investigate if mind wandering mediates the relationship between topic interest and memory performance a series of mediation analyses were computed with the ‘lavaan’ package in R (Rosseel, 2012) using bootstrapping with 5000 samples (reported in Table 7). First, to replicate the approach taken by Cherry et al. (2022), we tested if situational topic interest had an indirect effect on immediate memory performance via off-task thoughts. For this analysis, the outcome variable was immediate memory performance, the predictor variable was situational topic interest, and the mediator variable was off-task thoughts. To account for participants having two data points (performance from T1 and T2), participant ID was added as a random intercept when constructing the mediation model. Using this approach, and contradictory to Cherry et al., the effect of topic interest on immediate memory recall was not found to be mediated by off-task thoughts (*b* = 0.18, *SE* = 0.10, 95% CI [0.01, 0.39], *p* = .051). The second mediation model was built with situational topic interest as the predictor, delayed memory performance as the outcome, and off-task thoughts at T1 as the mediation variable. Although the mediation revealed a significant direct effect of situational topic interest on delayed memory performance (*b* = 0.50, *SE* = 0.23, 95% CI [0.01, 0.90], *p* = .026), off-task thoughts were not found to mediate this effect (*b* = 0.10, *SE* = 0.08, 95% CI [−0.03, 0.26], *p* = .183).

**Table 7:** Mediation models summarizing the effect of situational topic interest on memory retention via off-task thoughts and task-unrelated thoughts

		*B* (SE)	95% CI	*z* value	*p*
Model 1	Direct effect	0.11 (0.20)	[−0.28, 0.50]	0.56	.576
	Indirect effect	0.18 (0.10)	[0.01, 0.39]	1.95	.051
	Total effect	0.30 (0.18)	[−0.06, 0.66]	1.60	.110
Model 2	Direct effect	0.50 (0.23)	[0.01, 0.90]	2.23	.026
	Indirect effect	0.10 (0.08)	[−0.03, 0.26]	1.33	.183
	Total effect	0.60 (0.23)	[0.09, 1.00]	2.60	.108
Model 3	Direct effect	0.17 (0.18)	[−0.15, 0.53]	0.98	.329
	Indirect effect	0.13 (0.08)	[−0.02, 0.29]	1.60	.111
	Total effect	0.30 (0.19)	[−0.06, 0.66]	1.61	.108
Model 4	Direct effect	0.52 (0.23)	[0.01, 0.92]	2.27	.023
	Indirect effect	0.08 (0.08)	[−0.04, 0.25]	1.07	.287
	Total effect	0.60 (0.23)	[0.09, 1.00]	2.57	.010

Two further mediation analysis were conducted to investigate if the more specific index of mind wandering (i.e., the proportion of task-unrelated thoughts) mediates the relationship between memory recall and ratings of interest in the topic of the story (Table 7). In the third mediation model, immediate memory performance was entered as the outcome variable and situational topic interest was entered as the predictor variable; task-unrelated thoughts were entered as the mediator variable. For the final mediation analysis, ratings of situational topic interest were entered as the predictor, task-unrelated thoughts were entered as the mediator and delayed memory performance was entered as the outcome. Neither model revealed a significant indirect effect of topic interest on memory performance via mind wandering (for immediate memory: *b* = 0.13, *SE* = 0.08, 95% CI [−0.02, 0.29], *p* = .111; for delayed memory: *b* = 0.08, *SE* = 0.08, 95% CI [−0.04, 0.25], *p* = .287).

## Discussion

The present study applied a more nuanced approach to children’s off-task reports with the introduction of distinct off-task thought categories spanning task-unrelated thoughts, task-related interference, and inattention due to distraction. Using this approach, we aimed to examine the consistency of mind wandering reports across different testing sessions. A further aim was to examine whether mind wandering was predictive of both immediate and delayed memory performance, and whether mind wandering mediated the relationship between topic interest and memory recall. To summarize our key findings, we found that children reported engaging in mind wandering around 9% of the time, with more task-unrelated thoughts being reported during the listening activity at T1 (12%) compared with T2 (7%). Overall, children reported off-task thoughts around 24% of the time and this global estimate of inattention did not change significantly between T1 and T2; there were also strong correlations between levels of off-task thoughts across the two sessions. In the present study ‘pure’ mind wandering did not increase as a function of time-on-task, a finding frequently reported with young adults attending lectures (Farley et al., 2013; Risko et al., 2012; Varao-Sousa & Kingstone, 2019), although there was some limited and inconsistent evidence that other types of off-task thoughts increased over the testing period. Importantly, probe-caught ‘pure’ mind wandering, operationalized specifically as task-unrelated thoughts, significantly predicted how well 8- to 9-year-olds remembered key components of an audio story both immediately after listening to it and after a delay of 7 days. Contrary to our predictions, though, we did not find any significant indirect effect of topic interest on either immediate or delayed memory retention that was mediated by mind wandering. How each of these findings relate to the existing research literature will now be discussed.

### Types of inattentive episodes

One of our key aims was to provide a more precise estimate of childhood mind wandering. Motivated by the extant literature on adult populations (e.g., Kane et al., 2017, 2021; Stawarczyk et al., 2014; Unsworth & McMillan, 2014), we developed age-appropriate thought probes that would allow children to distinguish between task-unrelated thoughts, task-related interference, and thoughts rooted in distractions. Despite the fact that this involved children making a more complex judgment than simply whether their thoughts were on task or off task, the data revealed robust cross-session correlations within categories suggesting that children as young as 8 years of age were able to sort their off-task thoughts into the three aforementioned categories. Prior to this study, most estimates of probe-caught childhood mind wandering were obtained by asking children to simply distinguish if their thoughts were on-task or off task, with off-task thoughts being used as the measure of mind wandering (Cherry et al., 2022; Keulers & Jonkman, 2019; Ye et al., 2014; Zhang et al., 2015). The introduction of more specific thought probes suggests that ‘pure’ instances of mind wandering occurred just over 9% of the time, indicating that previous estimates of childhood mind wandering (20%–33%) may potentially have been inflated. That is, these previous estimates likely included other types of inattentive episodes rather than simply mind wandering. To the best of our knowledge, the only previous study with child participants that has looked in more detail at types of probe-caught inattentive episodes is that of Van den Driessche et al. (2017), who also distinguished between inattention due to distraction, task-related interference, and mind wandering (these authors included an additional category of mind blanking, the mind seeming empty, to test a hypothesis about the specific deficit in ADHD). They found a very similar level of ‘pure’ mind wandering in their sample of typically-developing control children (8.3%) aged 6–12 years, and moreover similar levels of inattention due to distraction as we did (8%–10%), despite the fact that they used a very different primary task (a multitrial go/no-go task) and testing context. Of note is that, when operationalized specifically as task-unrelated thoughts and distinguished from other types of inattentive episodes, levels of childhood ‘pure’ mind wandering appear to be below those reported by adults engaging in similar listening activities (20%–24%, Varao-Sousa et al., 2018) and in executive functioning tasks (21%, Stawarczyk et al., 2014; 13%–24%, Unsworth & McMillan, 2014).

There are a number of possible explanations for this apparent difference. It may be that there is no genuine developmental difference, but instead differences in reported levels of mind wandering between children in our study and adults in other studies reflect procedural differences, such as the nature of the primary task or the testing context in which it was set, and that if adults were tested in a similar way, we would find similar levels of mind wandering as in our child sample. One reason for thinking this is not the correct explanation is that Van den Driessche et al. (2017) tested both child and adult participants with almost identical procedures. Although they did not statistically compare these samples, inspection of their data (see Fig. 1 in Van den Driessche et al., 2017) suggests that adults were reporting substantially more instances of mind wandering than children (around double) but similar levels of inattentive episodes due to distraction. Thus, their data suggest that there may indeed be a developmental increase in levels of ‘pure’ mind wandering.

It was not the aim of our study was to examine developmental change, and thus we did not include different age groups, but we note that the idea that levels of ‘pure’ mind wandering increase developmentally is at least consistent with the claim that maintaining a continuous train of internally-generated thought is resource-dependent (Smallwood, 2013; Smallwood & Schooler, 2015) and thus may increase with cognitive development. At first sight, such a claim may seem at odds, for example, with existing findings suggesting that aspects of children’s executive function are either negatively associated with levels of off-task thoughts or show no relation to them (Keulers & Jonkman, 2019; Wilson et al., 2022). However, these previous developmental studies did not specifically isolate ‘pure’ mind wandering; moreover, as Smallwood and Schooler (2015) suggest, the relation between executive functioning and mind wandering may be complex and context specific. In particular, cognitive resources may play different roles with regard to preventing lapses in attention to a primary task (lapses may reflect a failure of cognitive control; McVay & Kane, 2010) and sustaining trains of internally generated thoughts (which may itself demand cognitive resources). The debate about the relation between cognitive resources and mind wandering remains ongoing (Wong et al., 2022), and our findings do not speak to this issue as we did not take any cognitive measures (other than levels of vocabulary). Nevertheless, when put alongside those of Van den Driessche et al. (2017), as well as findings suggesting levels of mind wandering decline at the other end of the lifespan (Jackson & Balota, 2012; Jordão et al., 2019; Maillet et al., 2018; Murphy et al., 2023; Seli et al., 2017), the results of the current study suggest it may be important to examine the developmental profile of ‘pure’ mind wandering in future studies. This would involve testing a broader age range of children as well as examining whether there are developmental changes beyond childhood.

In addition to examining mind wandering, we also included a category of inattentive episode that mapped to task-related interference. This was the most infrequently reported category in our study, and our data differ in this respect from those of Van den Driessche et al. (2017) who found it to be the most frequently reported type of inattentive episode in their child sample. These differences may be due to the different primary tasks used in the studies: children were listening to a story in our study, whereas in Van den Driessche et al.’s paradigm thought probes were inserted between trials in a go/no-go task; in the latter context it is plausible that participants were more likely to be monitoring how they were getting on in the task or might have been more likely to be evaluating what was an unfamiliar task. Such an interpretation would be compatible with findings suggesting levels of task-related interference are related to task difficulty (McVay et al., 2013; Zavagnin et al., 2014). As with ‘pure’ mind wandering, it would be useful to examine whether there is developmental change in levels of self-reported task-related interference, not least because, unlike mind wandering, levels of task-related interference seem to increase at the other end of the life span (Jordano & Touron, 2018; McVay et al., 2013).

### Consistency across sessions

A further aim of the current study was to assess the stability of mind wandering reports. We measured mind wandering twice, across two testing sessions that were similarly structured but involved different story content. Under these circumstances we found substantial across-session correlations in the overall proportion of off-task thoughts (*r* = 0.85). This link appears to be stronger than that reported by Keulers and Jonkman (2019; *r* = 0.41), speculatively because the primary task used in our study was very similar across the two sessions. Looking at the rates of ‘pure’ mind wandering specifically, the link between the frequency of task-unrelated thoughts reported across the two times points appears to be large (*r* = 0.67), consistent with the adult findings from McVay and Kane (2012) who reported that mind wandering measures from four different tasks as diverse as the go/no-go task and reading *War and Peace* loaded onto a single latent variable. Al-Balushi and Al-Harthy (2015) reported a similar finding concluding that probe-caught mind wandering rates during reading exercises were stable for university students across two types of chemistry based textual narrations (*r* = 0.42). With similar patterns emerging from the thought probes across both testing sessions in our study, these correlations suggest that the current procedure is a reliable way of measuring off-task thoughts in children, and indeed the measurements of all three subcategories of off-task thoughts showed this type of reliability. The cross-session correlations in mind wandering also suggest that the tendency to engage in mind wandering may potentially be a stable characteristic even in children, although establishing this would involve testing across a much wider range of task contexts.

We note that one difference between our method and that of other studies of children’s mind wandering was that we used a two-stage procedure in which children initially categorized their responses as on- or off-task and then were asked a further follow up question. The nature of that follow-up question varied depending on whether children had reported themselves to be on or off task: If children reported being on-task they were asked a filler question (about the number of sides on a simple shape), whereas if they were off-task they further subcategorized their off-task thought. Follow-up questions were asked in both instances to ensure the overall number of questions children were asked was constant, regardless of how often they were on or off task. However, we acknowledge that there may be other ways to probe children’s thoughts that avoid the need for follow-up questions, making filler questions unnecessary. For example, it might be possible to get children to give a single response to one more lengthy probe question (e.g., ‘*What were you thinking about just now? Select one of the following: ‘the story’, ‘other things happening right now’, ‘other things happening at other times’ or ‘things connected with the story’)*. Research on children’s mind wandering is still relatively limited and future studies need to establish exactly how best to elicit reliable self-reports from children.

### Mind wandering and learning

The preliminary link between mind wandering and learning found by Cherry et al. (2022) remained intact when using a more stringent index of mind wandering comprising only task-unrelated thoughts. As expected, high levels of mind wandering during the learning activities were predictive of poorer memory recall of the novel information presented within the audio stories. These results are in line with previous adult studies demonstrating a link between mind wandering and memory recall (e.g., Hollis & Was, 2016; Kane et al., 2017; McVay & Kane, 2012; Sanchez & Naylor, 2018; Smallwood et al., 2013; Wammes et al., 2016; Wammes & Smilek, 2017). In this study, we also found that all categories of off-task thoughts (task-unrelated thoughts, task-related interference and inattention due to distractions) were linked to poorer immediate memory performance. Regardless of their origin, off-task thoughts can impact instantaneous memory recall to varying degrees, indicating that, like mind wandering, other types of inattention can attenuate the learning process (Shelton et al., 2009; Varao-Sousa et al., 2018). However, further regression analyses suggested that task-related interference and inattention due to distractions were not significantly linked to delayed memory retention in the same way as task-unrelated thoughts.

This latter finding provides some support for the conceptual premise that mind wandering differs from other types of inattention (Barron et al., 2011; Smallwood et al., 2008). We found that the link between ‘pure’ mind wandering and memory was still present after a weeklong delay, consistent with the idea that mind wandering can particularly hinder the sort of deep semantic encoding of information that supports long term memory (Thomson et al., 2014). More broadly, if mind wandering affects children’s long-term retention of information, it may be linked to more global indices of academic achievement, in parallel to the way that probe-caught mind wandering aligns closely with longer term learning outcomes in adults, such as course grades (Kane et al., 2021; Mrazek et al., 2012, Wammes et al., 2016). To develop a more detailed account of the educational significance of mind wandering in the foundational years of schooling, future studies need to examine whether, and under what circumstances, indices of mind wandering serve as predictors of children’s scores on tests measuring academic performance in core subject areas. Establishing this could prove useful in developing strategies to enhance children’s learning in educational contexts by reducing mind wandering.

### Mind wandering and topic interest

At the outset of the study, we predicted that mind wandering would mediate the relationship between memory recall and topic interest, replicating previous work with adolescent and adult populations (Hollis & Was, 2016; Soemer et al., 2019; Unsworth & McMillan, 2013) and with children (Cherry et al., 2022). This finding did not emerge from the data. Topic interest did not have a significant indirect effect on memory recall via mind wandering or general off-task thoughts, contradicting several studies which found higher topic interest to be associated with less mind wandering in both youth and adult populations (e.g., Linquist & McLean, 2011; Seli et al., 2015; Soemer et al., 2019; Unsworth & McMillan, 2013). This inconsistency may have been driven by a procedural detail – namely, the fact that topic interest ratings were obtained twice. Children were first asked to rate their interest in the *overall story topic* prior to listening to it using a five-point Likert scale. Then after listening to the audio recording, children were asked to use the same scale to report their interest in the *actual story* they had just listened to. It is a possibility the scales were worded too similarly, and a strong body of research suggests repeated questioning can lower the veracity and consistency of children’s answers (Bonawitz et al., 2020; Fivush & Schwarzmueller, 1995). Alternatively, the association between topic interest, mind wandering, and memory performance is not as consistent and salient for younger children as it is for adults. Future work taking into account other factors that are often investigated in parallel with topic interest (e.g., task difficulty and motivation; Guthrie et al., 2007; Hidi & Harackiewicz, 2000; Soemer & Schiefele, 2019; Unsworth & McMillan, 2013) is needed to expand our understanding of how topic interest is likely to influence childhood mind wandering and learning.

## Conclusion

We set out to improve our knowledge of mind wandering in children by focusing on three specific issues. First, we sought to provide a more precise measure of mind wandering in children and to this end, our findings suggested that 8- to 9-year-olds are appropriately able to distinguish between attentional lapses that are rooted in task-unrelated thoughts, task-related interference, and those that are a result of distraction. The second aim of the present study was to assess the stability of mind wandering reports in children; we found that probe-caught mind wandering remained stable across both testing sessions. Our final objective was to explore the impact of mind wandering on delayed memory recall. This study was the first to uncover task-unrelated thoughts, as opposed to general off-task thinking, as an important predictor for both immediate and delayed memory recall in children. Taken together, the current findings indicate greater investigation on children’s attentional focus during learning activities within the classroom could provide a springboard for the development of strategies geared towards equipping children with the necessary skills to detect and refocus lapses of attention to improve overall learning outcomes.

### Supplementary Information

Below is the link to the electronic supplementary material.

Supplementary file1 (DOCX 13422 KB)

## Data Availability

Materials, data, and analysis code are available on the Open Science Framework at https://osf.io/76chp/?view_only=68fbcc54b138435ca3797f74244166c7. The authors declare no conflict of interest.
