# Supplementary material for: Listen up, kids! How mind wandering affects immediate and delayed memory in children
Source: Mem Cognit. 2023 Dec 27;52(4):909–25. doi: 10.3758/s13421-023-01509-0 (PMC11111549; doi:10.3758/s13421-023-01509-0)
Supplement: Supplementary file 1 — Supplementary file1 (DOCX 13422 KB) [file 13421_2023_1509_MOESM1_ESM.docx]

Supplement to ‘Listen up, kids! How mind wandering affects immediate and delayed memory in children.’

Abstract

This document presents supplementary material for the manuscript “Listen up, kids! How mind wandering affects immediate and delayed memory in children.”

*Keywords:* mind wandering, task-unrelated thoughts, cognitive development, learning

**Contents**

1. Final sample characteristics…………………………………………………………....2
2. Sorting activity………………………………………………………………………...3
3. Training activity…………………………………………………………….……...….7
4. Audio story 1 transcript………………………………………………………………15
5. Audio story 2 transcript………………………………………………………............20
6. Memory recall tests…………………………………………………………………..25

___________________________________________________________________________

**1. Final sample characteristics**

The final sample characteristics for participating children.

*Table 1* Final sample characteristics.

| Variable | N | Mean (SD) |
| --- | --- | --- |
| Age (in years) | 60 | 8.99 (0.52) |
| Parent proximity at Time 1 |  |  |
| Beside child | 21 | 35.00% |
| Same room | 26 | 43.33% |
| Other room | 13 | 21.67% |
| Parent proximity at Time 2 |  |  |
| Beside child | 12 | 20.00% |
| Same room | 21 | 35.00% |
| Other room | 27 | 45.00% |
| WISC Vocabulary |  |  |
| Raw | 60 | 24.87 (5.03) |
| Scaled | 60 | 10.92 (2.66) |

**2. Sorting activity**

When the participants first logged on to their testing session, they were introduced to a fictional character who to explain the nature and depictions of the different categories of thought. The children would then engage in an initial sorting activity which consisted of placing sample off-task thoughts into one of the three categories (task-unrelated thoughts, task-related interference, or external distractions). Provided below is the precise wording used in to explain on-and off-task thoughts. The screens that accompanied the sorting activity are also supplied below.

*Researcher:* Now I would like to introduce you to my friend panda. Panda can you say hello?


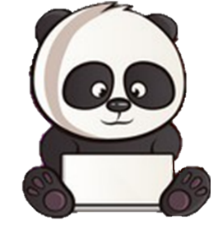


*Panda:* Hello

*Researcher:* Panda is listening to a story on the laptop. Just like you will be listening to a story in a short while. Panda is trying hard to listen to the story, but always listening hard is impossible! Often panda might start to think of something other than the story. Let’s have a look at some of panda’s thoughts…


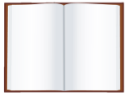

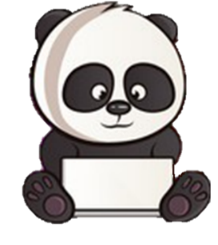


You see here panda is thinking about the story they are listening to and nothing else. Panda’s thoughts match what Panda is doing, and no other thoughts have popped into Panda’s head, Panda is just thinking about what is being said in the story.


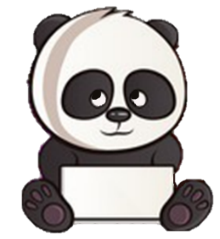


Now Panda isn’t really listening to what is being said in the story anymore and other thoughts have popped into Panda’s head.


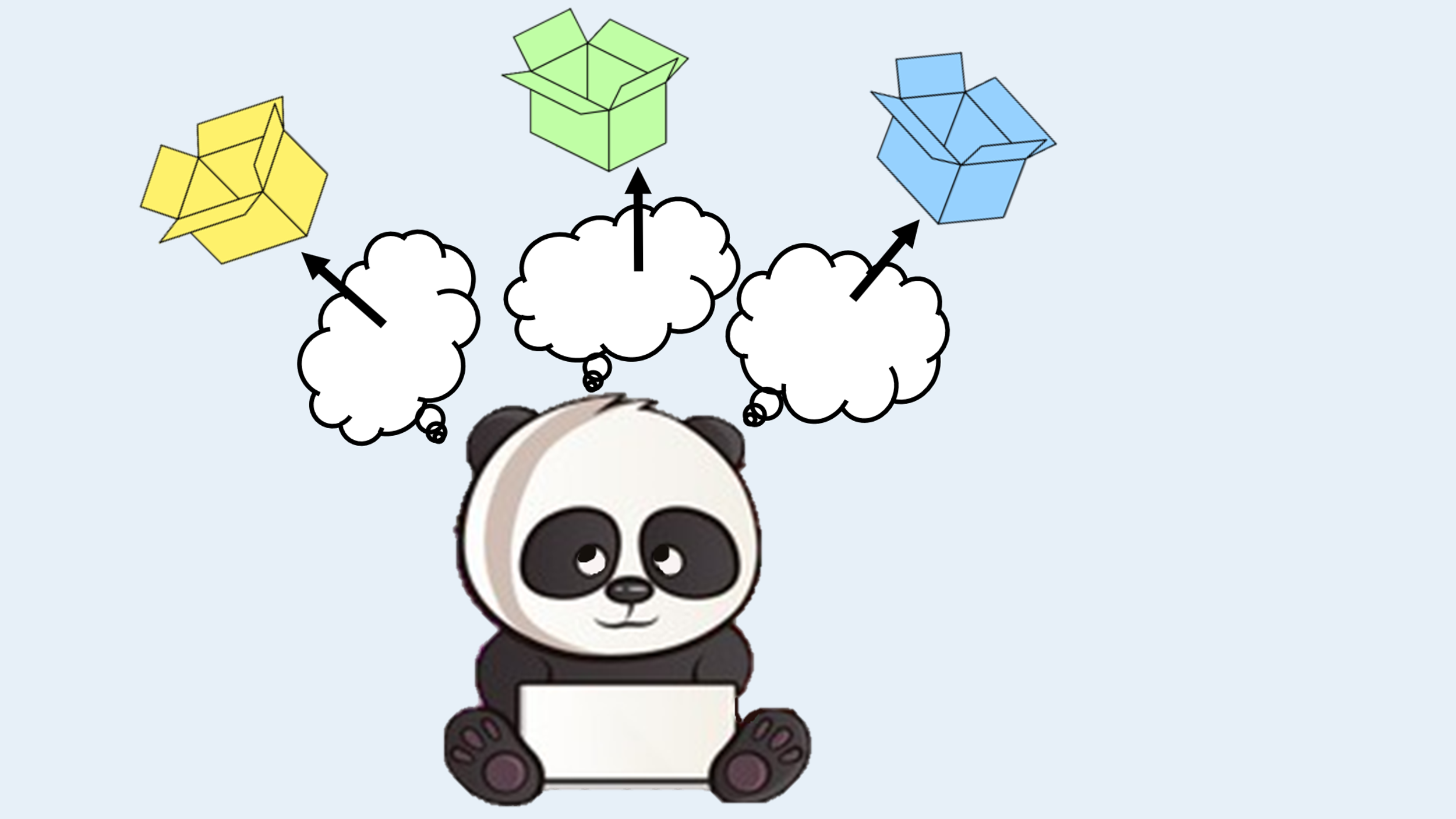


There can be different sorts of thoughts. I’m going to explain to you now the different kinds of thoughts and we are going to sort them into different boxes.


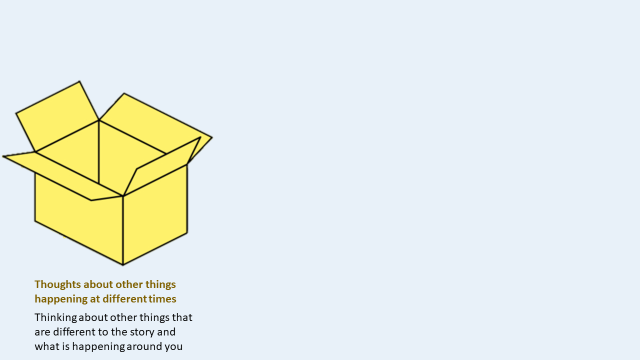
First there is this box which is for thoughts about other things that are completely different to what is happening around you. We will call these "thoughts about other things happening at different times". For example, thoughts about playing with your friends earlier today, or thoughts about what you might have for dinner later, or what homework you have to do will go inside this box.


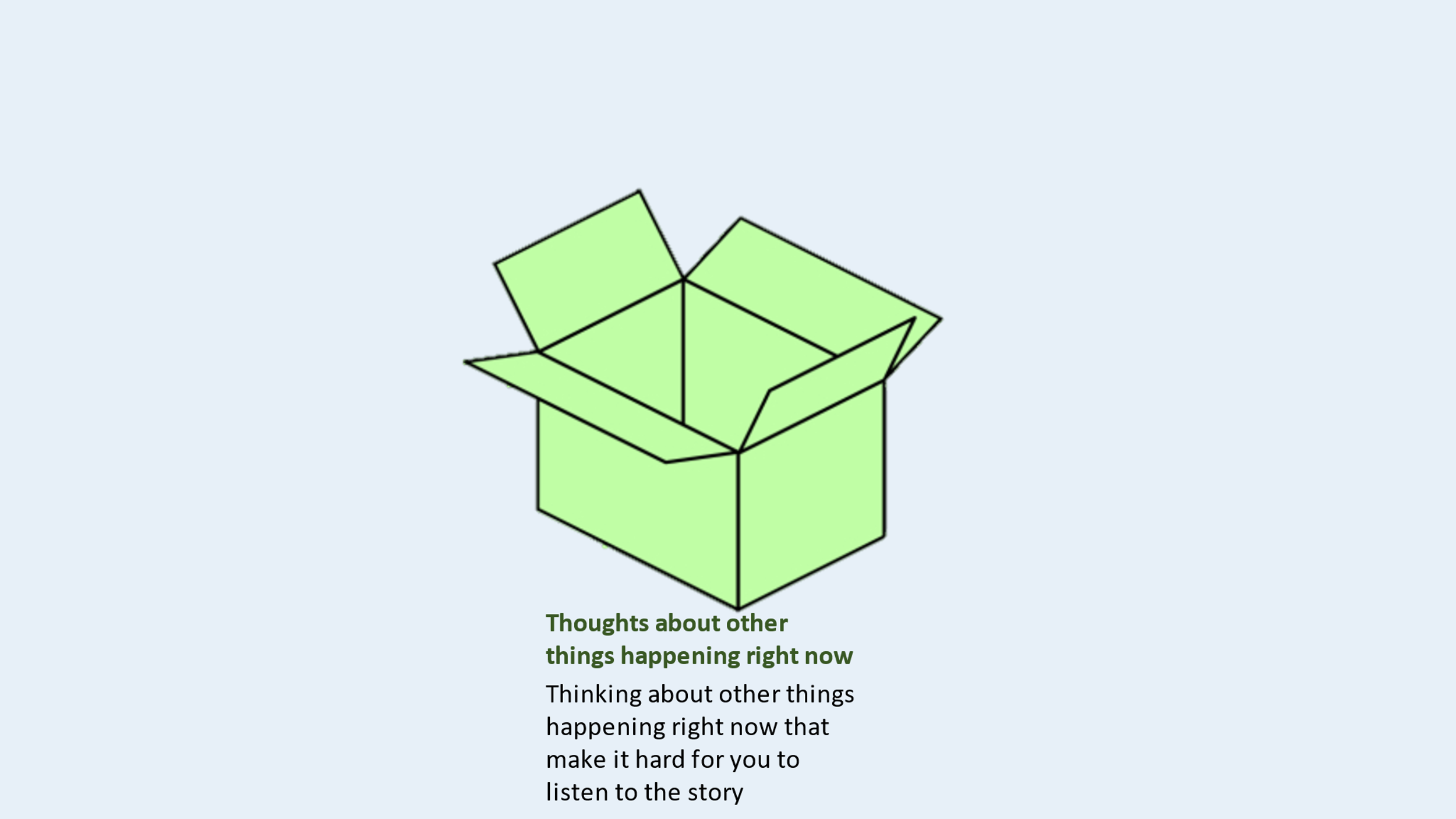


Next, we have this box which is for thoughts about other things happening right now that make it hard for you to listen to the story. We will call these “thoughts about other things happening right now”. For example, thoughts about noises you can hear outside, or noticing your mum/dad coming in or leaving the room will go inside this box.


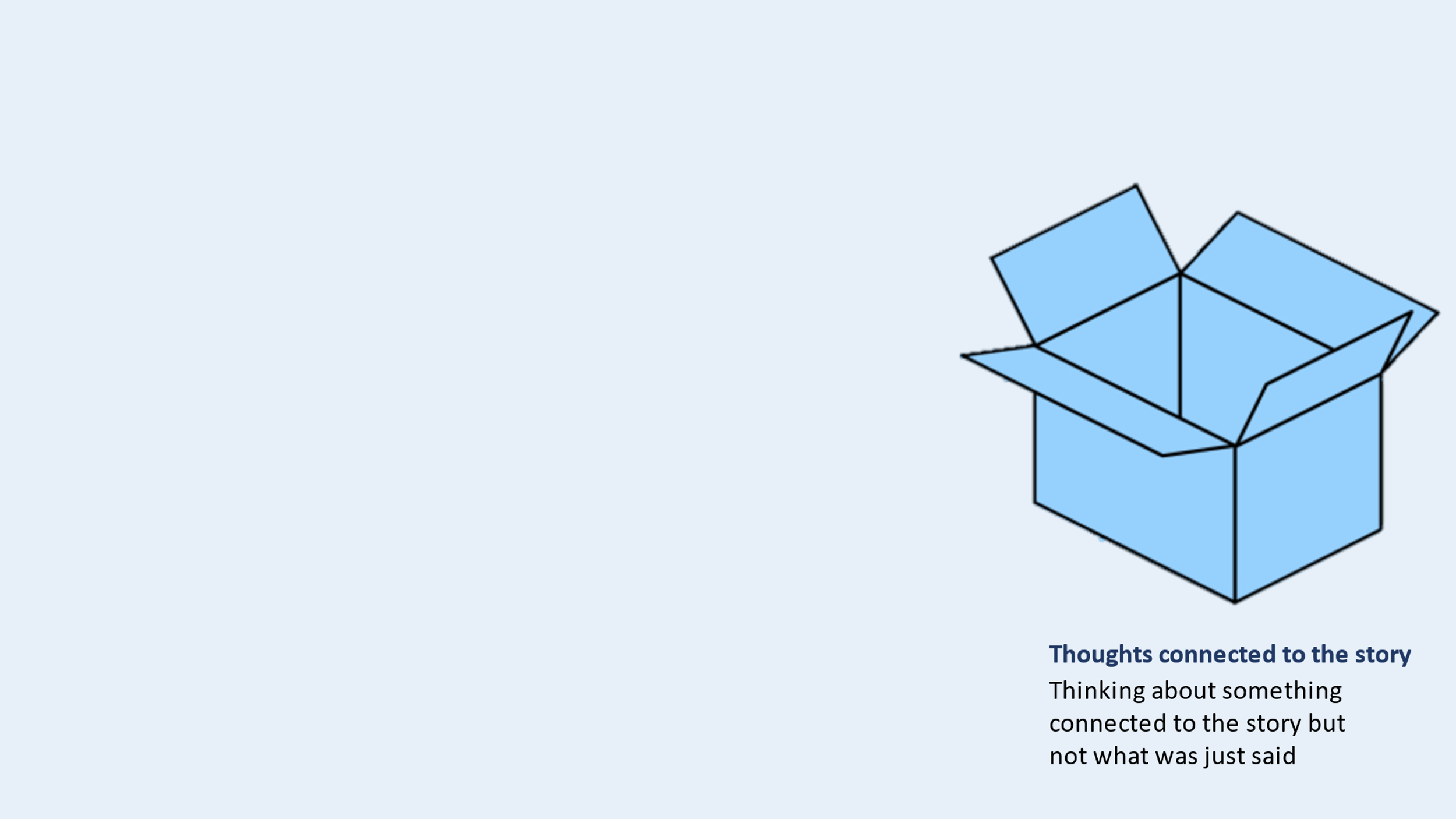
Lastly, we have this box for thoughts that are different from what was just said in the story but are still connected to the story. We call these “thoughts connected to the story”. For example, noticing that you're not paying attention and that you should try to ‘focus’ on the story, or wondering if you will remember a fact from the story, or guessing what the quiz will be like will go inside this box.


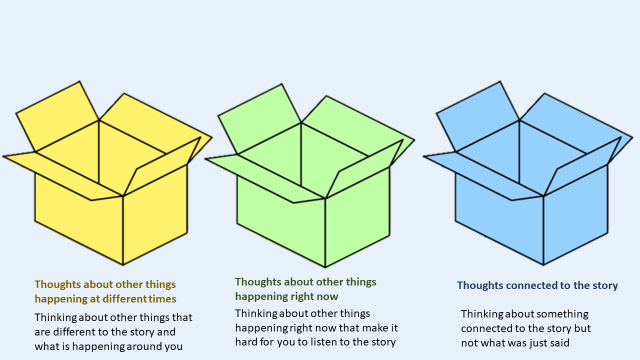


These are the boxes we can sort the different kinds of thoughts into. Would you like to play a game sorting Panda’s thoughts into these boxes?

***Sorting Activity***


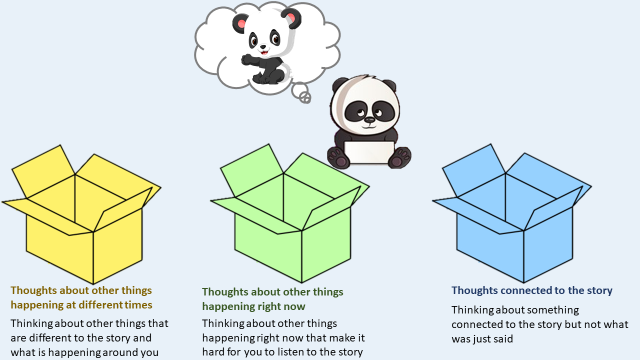


Panda is supposed to be thinking about what was just said in the story but instead is thinking about their baby sister. She has crawled into the room and now Panda is thinking about her…where should we put this thought? Is the thought connected to the story, is it a thought about other things happening at different times, or is Panda thinking about other things happening right now?

*Wait for child’s response*

*If correct:* Yes, that’s right! Panda’s thought is about other things happening right now.

*If incorrect:* Not quite right, Panda’s thought is about other things happening right now.


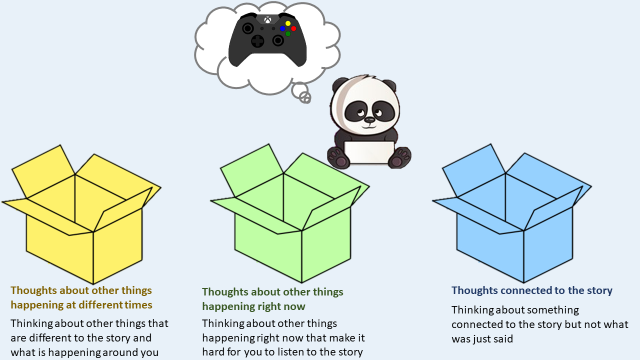
Panda is supposed to be thinking about what was just said in the story but instead Panda is thinking about playing games on the xbox later. Where should we put this thought? Is the thought connected to the story, is it a thought about other things happening at different times, or is Panda thinking about other things happening right now?

*Wait for child’s repsonse*

*If correct:* Yes, that’s right! Panda’s thought was about other things happening at different times.

*If incorrect:* Not quite right, Panda’s thought was about other things happening at different times.


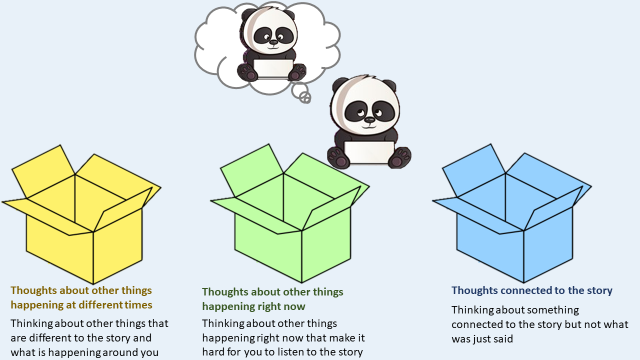


Panda is supposed to be thinking about what was just said in the story but instead Panda is thinking: "Uh oh, I'm not doing my best… I should be listening to the story more carefully so that I don't miss anything important". Where should we put this thought? Is the thought connected to the story, is it a thought about other things happening at different times, or is Panda thinking about other things happening right now?

*Wait for child’s response*

*If correct:* Yes, that’s right! Panda’s thought is about something connected to the story.

*If incorrect:* Not quite right, Panda’s thought is about something connected to the story.

**3. Training activity**

Successful completion of training trials was a prerequisite to taking part in the mind wandering task. This process helped to ensure all children could comprehend the thought probes and answer them accordingly. Children received immediate feedback on their responses during the training phase. On the first attempt, 93% of children passed the exercise and, on the second attempt, the remaining 7% successfully completed the training. Provided below is the precise wording used in the training exercise. The screens that accompanied the training exercise are also supplied below.

***Training Activity Q1-Q4***


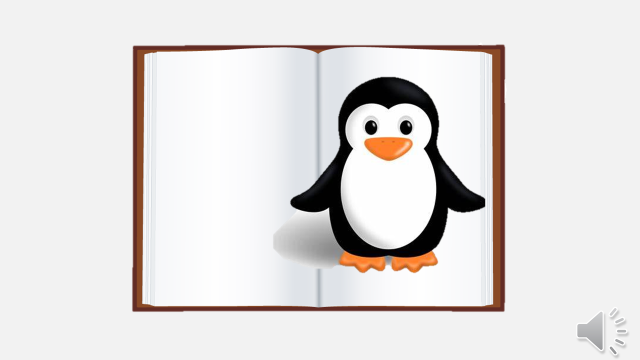


Panda is listening to a story about penguins, shall we listen with Panda and have a go at answering some questions about Panda’s thoughts?


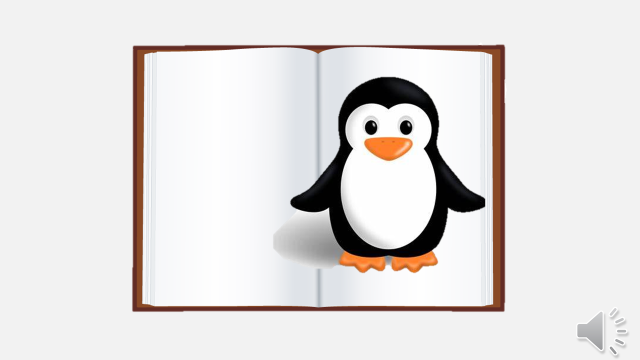


Penguins are a group of flightless birds. Almost all penguins live in the Southern Hemisphere, with only one species, the Galápagos penguin, found in the Northern Hemisphere.

*Researcher*: Panda! What were you thinking about just now?

WHAT WERE YOU THINKING ABOUT JUST NOW?


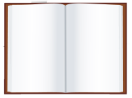

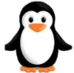

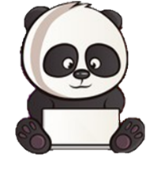

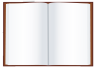

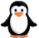


*Panda*: Penguins are found in the Southern & Northern Hemisphere!

*Researcher*: So, which answer should we pick? Is panda thinking about what has just been said in the story or something different?

*Wait for child’s response*

*Researcher:* Panda was thinking about what was just said in the story! Let’s continue with the story.


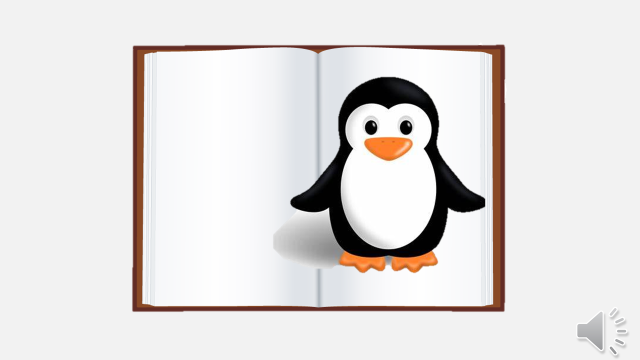


Penguins are made for life in the water. They have a black and white coat which is perfect for swimming. Most penguins eat krill, fish, squid and other forms of sea life. They catch these while swimming underwater.

WHAT WERE YOU THINKING ABOUT JUST NOW?


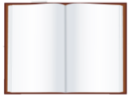

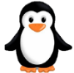

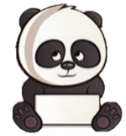

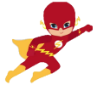


*Researcher:* Panda! What were you thinking about just now?

*Panda*:  A superhero movie I watched last night.

*Researcher:* So, what should we answer? Is panda thinking about what has just been said in the story or something different?

*Wait for the child’s response.*

*Researcher:* Panda was thinking about something different than what was just said in the story.


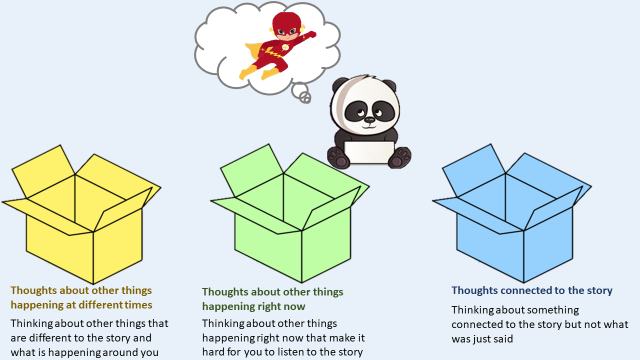


Panda was supposed to be thinking about what was just said in the story but instead is thinking about a superhero movie. Where should we put this thought? Is the thought connected to the story, is it a thought about other things happening at different times, or is Panda thinking about other things happening right now?

*Wait for child’s response*

*If correct:* Yes, that’s right! Panda’s thought is about other things happening at different times.

*If incorrect:* Not quite right, Panda’s thought is about other things happening at different times.


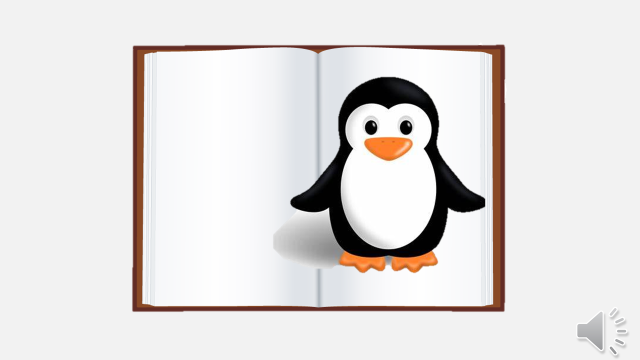


Penguins spend roughly half of their lives on land and the other half in the sea. The largest living species is the emperor penguin and the smallest penguin species is the little blue penguin, also known as the fairy penguin.

WHAT WERE YOU THINKING ABOUT JUST NOW?


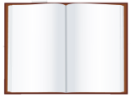

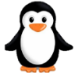

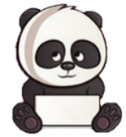

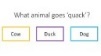


*Researcher*: Panda! What were you thinking about just now?

*Panda*: I was wondering what I’ll need to remember for the quiz.

*Researcher*: So, which answer should we pick? Is panda thinking about what has just said in the story or something different?

*Wait for child’s response*

Panda was thinking about something different than what was just said in the story.


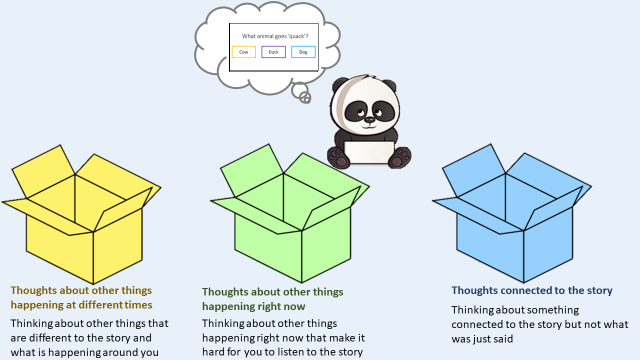


Panda was supposed to be thinking about was just said in the story but instead Panda is thinking about they will need to remember for the quiz. Where should we put this thought? Is the thought connected to the story, is it a thought about other things happening at different times, or is Panda thinking about other things happening right now?

*Wait for child’s response*

*If correct:* Yes, that’s right! Panda’s thought was connected to the story.

*If incorrect:* Not quite right, Panda’s thought was connected to the story.


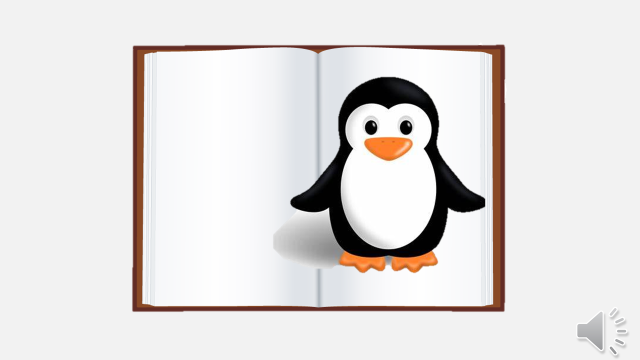


The larger penguin species of penguins live in colder regions, and the smaller penguin species like to live in slightly warmer climates.

*Researcher:* Panda! What were you thinking about just now?

WHAT WERE YOU THINKING ABOUT JUST NOW?


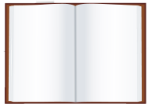

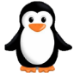

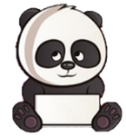

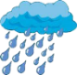


*Panda*:  I was thinking about the weather, I can hear the rain hitting off my window!

*Researcher:* So, what should we answer? is panda thinking about what has just been said in the story or something different?

*Wait for child’s response*

*Researcher:* Panda was thinking about something different than what was just said in the story.


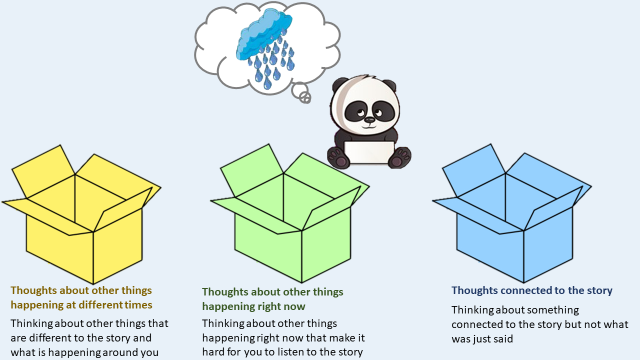
Panda was supposed to be thinking about what was just said in the story but instead Panda is thinking about the rain hitting off their window. Where should we put this thought? Is the thought connected to the story, is it a thought about other things happening at different times, or is Panda thinking about other things happening right now?

*Wait for child’s response*

*If correct:* Yes, that’s right! Panda’s thought is about something happening right now.

*If incorrect:* Not quite right, Panda’s thought is about something happening right now.

*If the children have successfully completed the training without errors thus far, they may continue to the listening activity. Otherwise, four more training questions will be played.*

***Training Activity Q5-Q8***


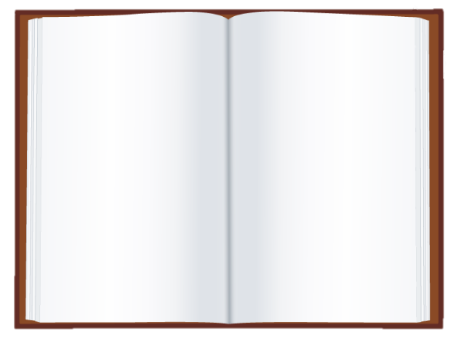

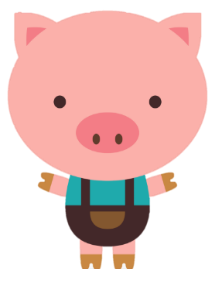


Panda is listening to a story about three little pigs, shall we listen with Panda and have a go at answering some more questions about Panda’s thoughts?


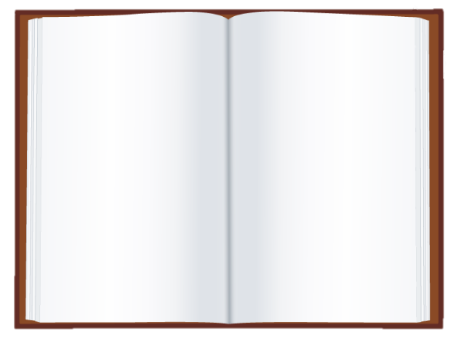

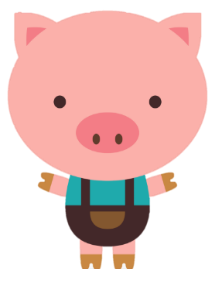


Once upon a time there were three little pigs, called Parker, Perry and Patty. They decided they would each like to build a house of their own.

WHAT WERE YOU THINKING ABOUT JUST NOW?


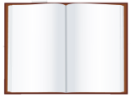

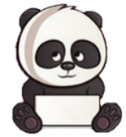

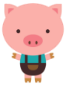

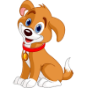


*Researcher:* Panda! What were you thinking about just now?

*Panda*:  I could hear my dog barking so I was thinking about what could be making the dog bark.

*Researcher:* So, what should we answer? Is panda thinking about what has just been said in the story or something different?

*Wait for child’s response*

*Researcher:* Panda was thinking about something different than what was just said in the story.

**
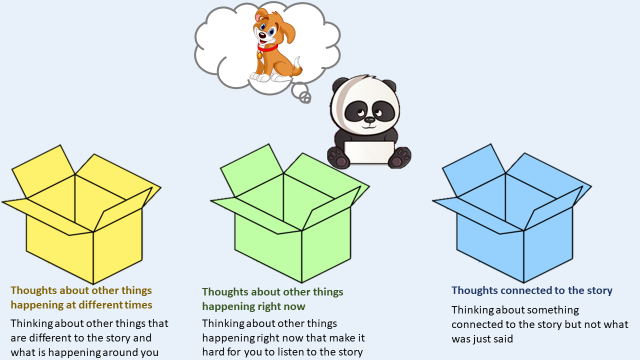
**Panda was supposed to be thinking about what was just said in the story but instead Panda is thinking about what is making the dog bark. Where should we put this thought? Is the thought connected to the story, is it a thought about other things happening at different times, or is Panda thinking about other things happening right now?

*Wait for child’s response*

*If correct:* Yes, that’s right! Panda’s thought is about something happening right now.

*If incorrect:* Not quite right, Panda’s thought is about something happening right now.


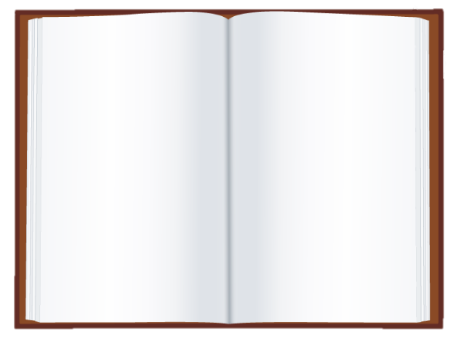

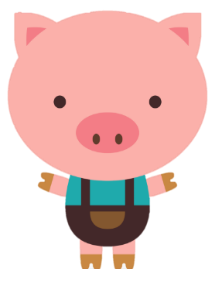


Parker decided to build a straw house, Perry thought it would be a good idea to build a house with sticks and Patty wanted to build a house with bricks.

*Researcher*: Panda! What were you thinking about just now?

WHAT WERE YOU THINKING ABOUT JUST NOW?


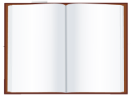

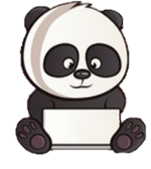

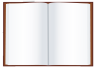

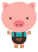

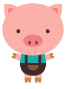


*Panda*: Patty building a house with bricks.

*Researcher*: So, which answer should we pick? Is panda thinking about what has just been said in the story or something different?

*Wait for child’s response*

*Researcher:* Panda was thinking about the story! Let’s continue with the story.


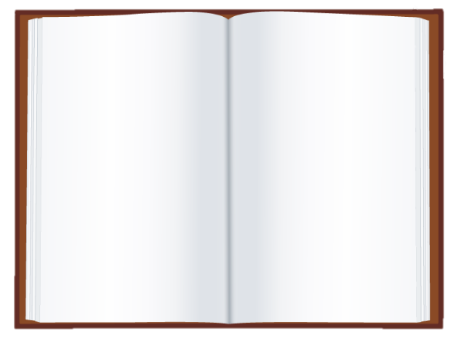

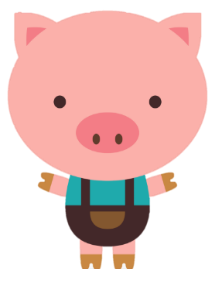


One day the big bad wolf came to town. The big bad wolf huffed and puffed and easily blew down the house made of straw. So, Parker quickly ran to the Perry’s stick house.

*Researcher:* Panda! What were you thinking about just now?

WHAT WERE YOU THINKING ABOUT JUST NOW?


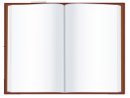

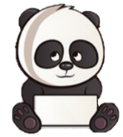

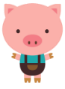

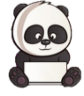


*Panda*:  I was thinking that I need to try harder to listen to what is being said in the story.

*Researcher:* So, what should we answer? Is panda thinking about what has just been said in the story or something different?

*Wait for child’s response*

*Researcher:* Panda was thinking about something different than what was just said in the story.

**
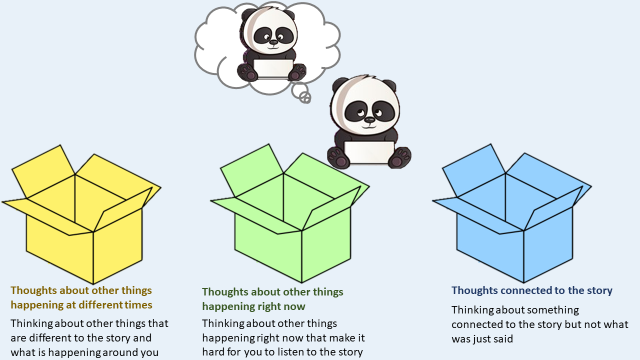
**

Panda was supposed to be thinking about what was just said in the story but instead Panda is thinking about how Panda should focus on what is being said in the story. Where should we put this thought? Is the thought connected to the story, is it a thought about other things happening at different times, or is Panda thinking about other things happening right now?

*Wait for child’s response*

*If correct:* Yes, that’s right! Panda’s thought is connected to the story.

*If incorrect:* Not quite right, Panda’s thought is connected to the story.


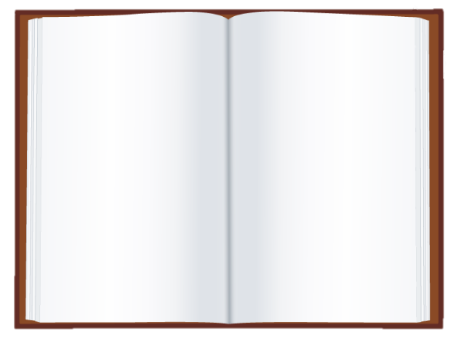

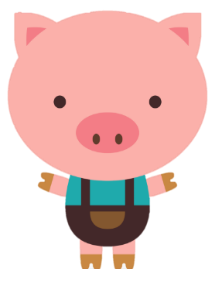


The big bad wolf huffed and puffed and blew down the stick house! Parker and Perry ran to Patty’s brick house. The wolf huffed and puffed but nothing happened, the big bad wolf could not blow down the brick house. Parker, Perry, and Patty were safe from the big bad wolf.

WHAT WERE YOU THINKING ABOUT JUST NOW?


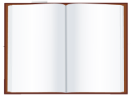

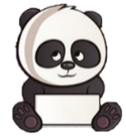

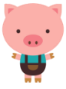

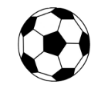


*Researcher:* Panda! What were you thinking about just now?

*Panda*:  I was thinking about how much fun I had playing football with my friends earlier.

*Researcher:* So, what should we answer? Is panda thinking about what has just been said in the story or something different?

*Wait for child’s response*

*Researcher:* Panda was thinking about something different than what was just said in the story.


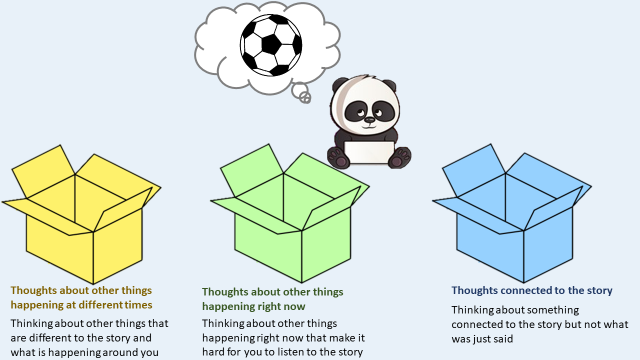
Panda was supposed to be thinking about what was just said in the story but instead Panda is thinking about how much Panda enjoyed playing football earlier today. Where should we put this thought? Is the thought connected to the story, is it a thought about other things happening at different times, or is Panda thinking about other things happening right now?

*Wait for child’s response*

*If correct:* Yes, that’s right! Panda’s thought is about other things happening at different times.

*If incorrect:* Not quite right, Panda’s thought is about other things happening at different times.

**4. T1 audio story transcript**

Mind wandering at time 1 was measured via intermittent thought probes embedded into an audio story about ancient Egypt. Please find below the transcript of the audio story alongside the screen presentation. When ‘***Thought probe***’ appears in the subsequent text it denotes that a thought probe occurred at this point. The following story is an original piece that was written for the purpose of this research.


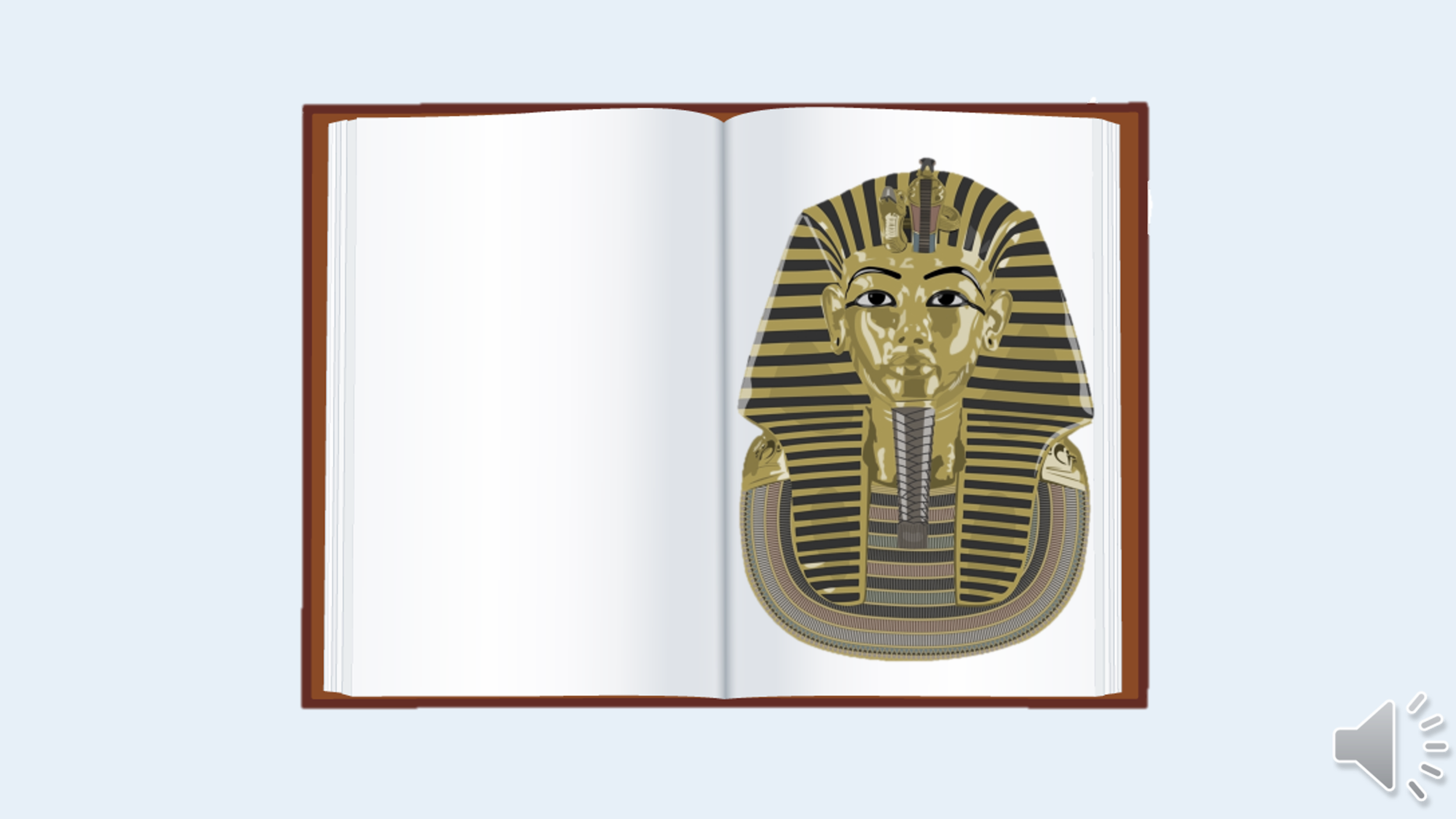
*Recording 1 of 8: 211 words lasting 88 s* Today, I am going to tell you a story about an Egyptian Pharaoh from a very long time ago. This Pharaoh was called Olufemi, during this story I will call Olufemi ‘Olu’ for short. You have maybe heard about ancient Egypt; it was one of the first civilisations in the world and people today are still fascinated by it.  A civilisation is a group of people who share things like language, laws and a certain way of life. You are living in a civilisation right this second! Anyway, back to ancient Egypt, so, where was ancient Egypt? Ancient Egypt and modern-day Egypt is located in the northeastern part of Africa. Africa is one of the world’s seven continents. A continent is basically a large solid area of land and there can be many countries within a continent. So, Egypt is one of the countries which is located in the continent of Africa. Ancient civilisations lived near rivers this was because of the water. These people, like us, needed the water to drink and to water their crops. Crops are grown to keep us alive. There are different kind of crops, but for an ancient civilisation food crops would have probably been the most important. So early civilisations needed rivers for water and food.

***Thought probe (example below).***Note that all other probes will follow the same structure.


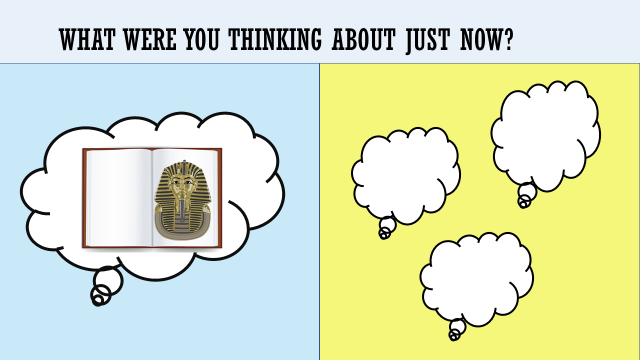


What were you thinking about just now? What was just said in the story or something different? Remember there are no right or wrong answers.


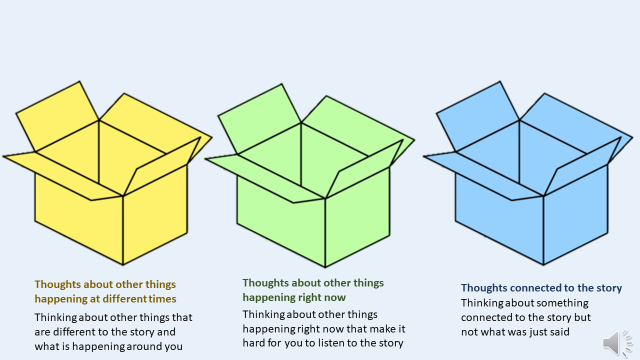
*(if ‘Something Different’)*

Can you tell me in which box does your thought belong? Is the thought connected to the story, is it a thought about other things happening at different times, or is it about other things happening right now?


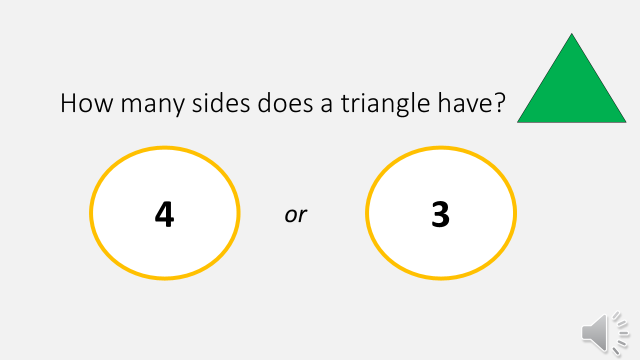
*(if ‘The Story’)*

How many sides does a triangle have? 4 or 3?

**Listening Activity Transcript continued**


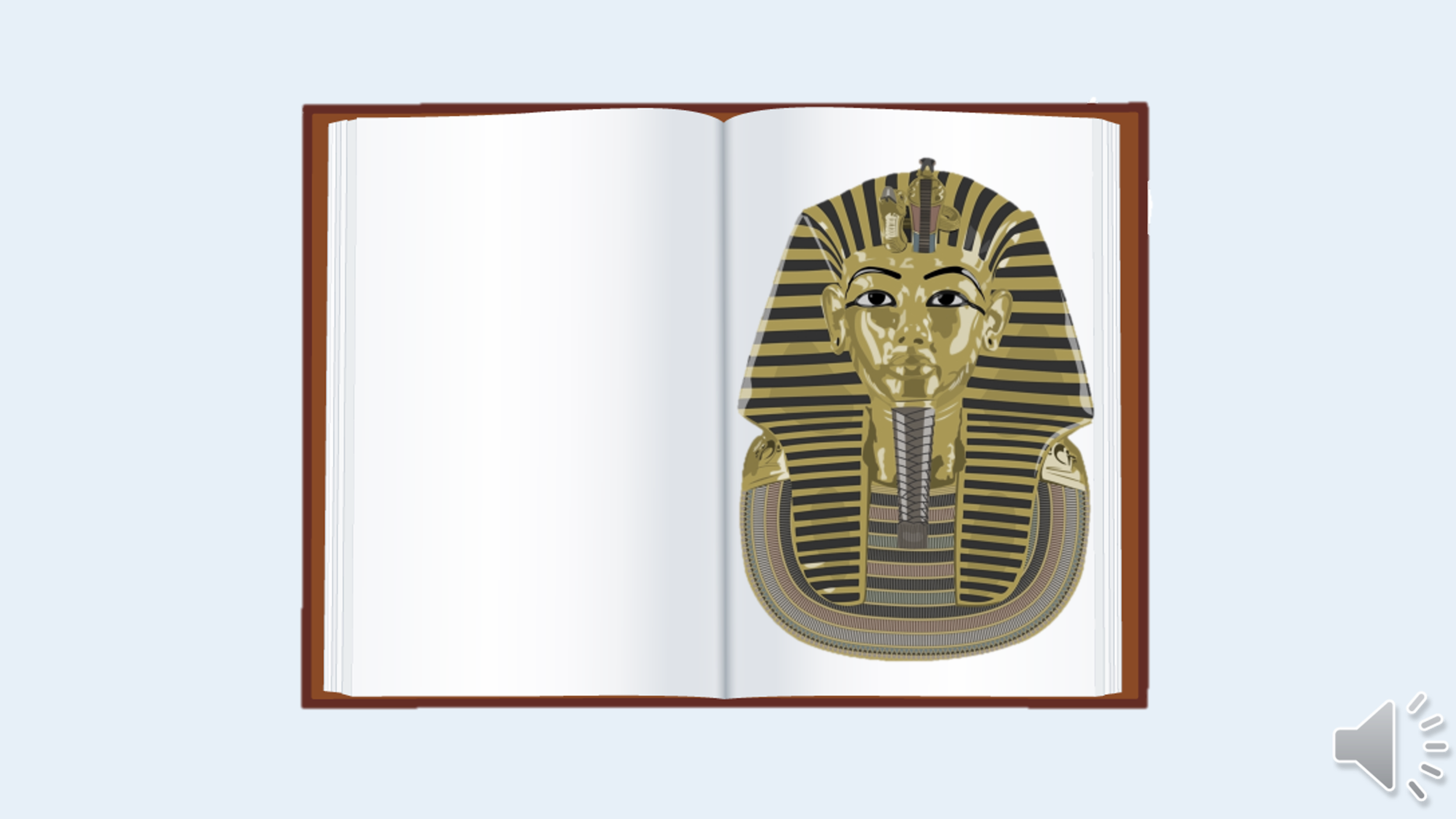
*Recording 2 of 8: 234 words lasting 90 s*

The river that was near the civilisation of Ancient Egypt was the river Nile. The river Nile still runs through Africa today; it is actually the longest river in the world.  The same river that helped the people of ancient Egypt is still helping people today. The river Nile really helped make ancient Egypt such a great early civilisation. The Egyptian pyramids are well known; they are ancient pyramid-shaped structures located in Egypt. The pyramids are large structures with four sides that slope upwards to meet at a point, the point is called the apex. The sides are made up of triangles but the pyramids in Egypt are actually known as square pyramids this is because their bases are square. It is amazing that they were built a very long time ago and they are still standing today! Most of the Egyptian pyramids were built as tombs for the country’s important people during ancient times. What that means is that in ancient Egypt, when someone important had died their body would be placed in a tomb underneath the pyramids. Ancient Egyptians were kept busy by building pyramids! Have you ever heard of an archaeologist? An archaeologist is a person who looks at very old objects and places to learn things about the past. Archaeologists have found over 130 pyramids in Egypt and they are still looking for more! The pyramids would get built very, very slowly.

***Thought probe***


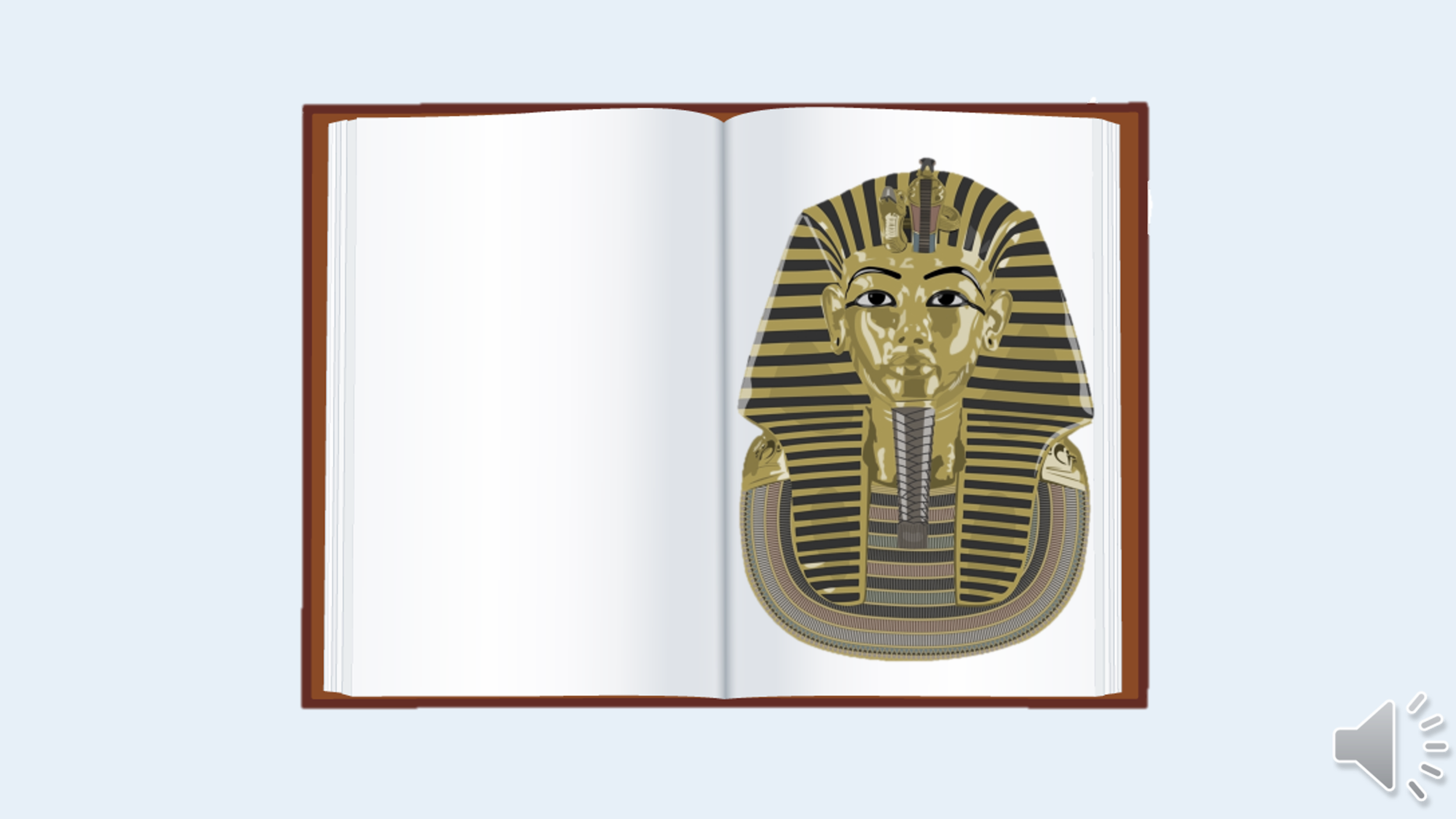


*Recording 3 of 8: 227 words lasting 88 s*

The Great Pyramid of Giza took 23 years to build but that is hardly surprising when you think about the weight of those blocks of rock and how high the pyramid is. It is believed that it took about 20,000 workers to build it. Amazing. I will come back to this later, but the Great Pyramid of Olu also took a long time to build, about 19 years! There are some theories as to how the pyramids were built, but no-one knows for sure. The Ancient Egyptians were great at inventing and invented a lot of the things we still use today!  They invented medicine, musical instruments, paper, pens, locks, keys, make up and even… toothpaste! People had many different jobs in ancient Egypt, there were farmers, craftspeople, soldiers, and priests and priestesses. There were also people called scribes, who were very important in running the country and they were considered to be very powerful. Why? Well, they were the only people who could read and write, and they helped run the country. Pharaohs in ancient Egypt wore ‘false beards’, this accessory was very important for making a Pharaoh look very powerful. Most false beards were made from metal, such as copper, gold, silver and attached to the Pharaoh’s chin. Pharaoh Olu’s false beard was made out of lead. Both men and women Pharaohs wore false beards.

***Thought probe***


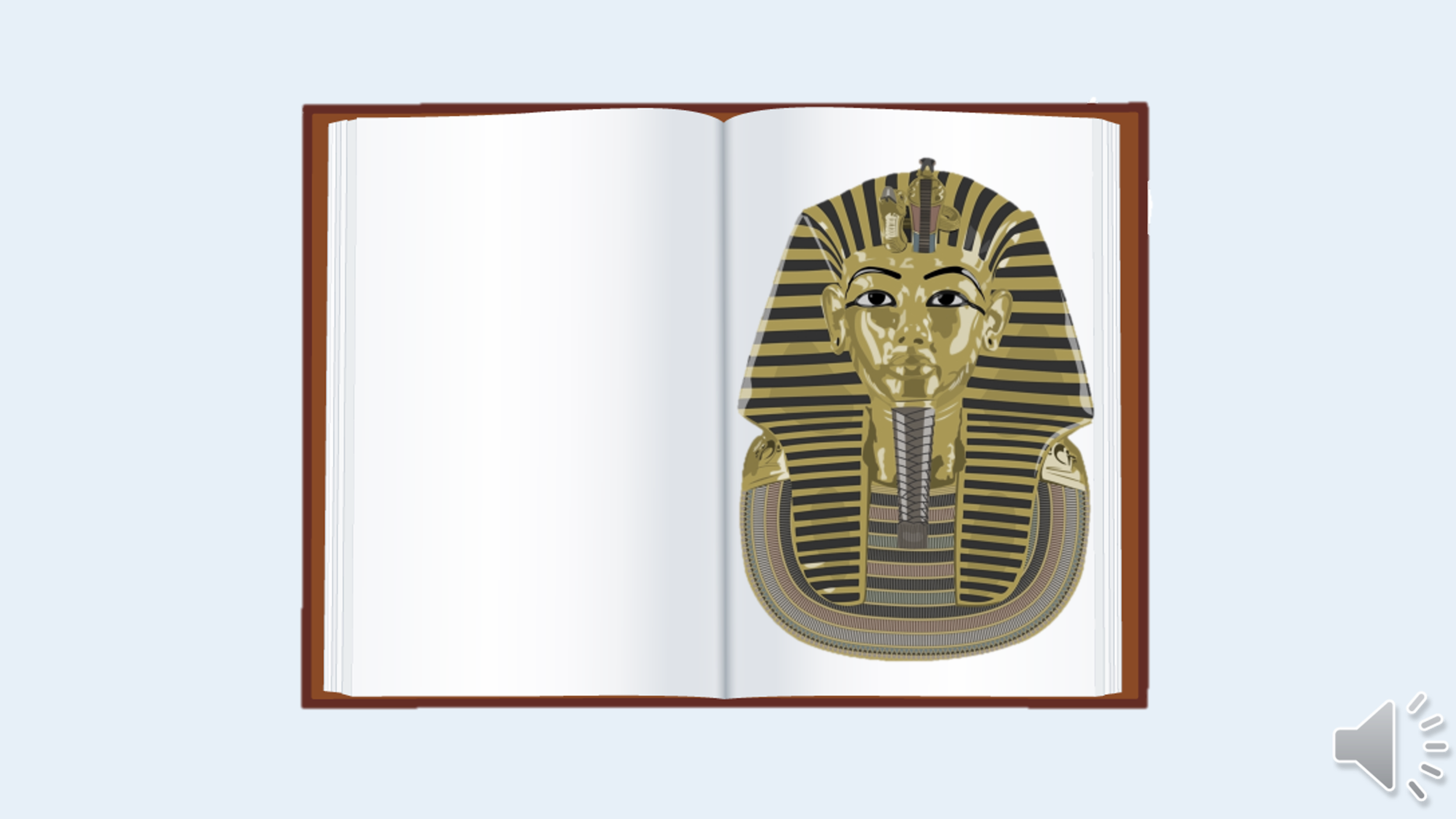
*Recording 4 of 8: 239 words lasting 93 s*

So, I’m going to tell you about a Pharaoh in ancient Egypt. The Pharaoh was the king or queen of everything in ancient Egypt. The people believed the Pharaoh was a child of the gods and after death the Pharaoh would also become a god. You have perhaps not heard much about Pharaoh Olu before; Pharaoh Olu was born a very long time ago in the year 900BC. Olu became an Egyptian pharaoh at 12 years old, making Olu one of the youngest Pharaoh’s ever! We didn’t know about Olu for a very long time, the tomb under the pyramid dedicated to Pharaoh Olu was left undiscovered for over 3000 years, it wasn’t until very recently that people finally found the tomb of Pharaoh Olu! But let us go back to when Pharaoh Olu was alive! Olu had just become the Pharaoh of ancient Egypt. When someone became a Pharaoh in ancient Egypt, they had to wear big headdresses and lots of jewellery. Olu liked to wear lots of jewellery but most of all Olu loved big and colourful headdresses. Pharaoh Olu’s favourite one was a dazzling gold with vibrant blue stripes! This matched Olu’s false beard which was decorated with blue spots. Pharaoh Olu also liked to wear red robes; the fabric of Olu’s robes was made from crinkle cotton. This fabric is great for helping people stay cool, this was important as the sun is very hot in Egypt!

***Thought probe***


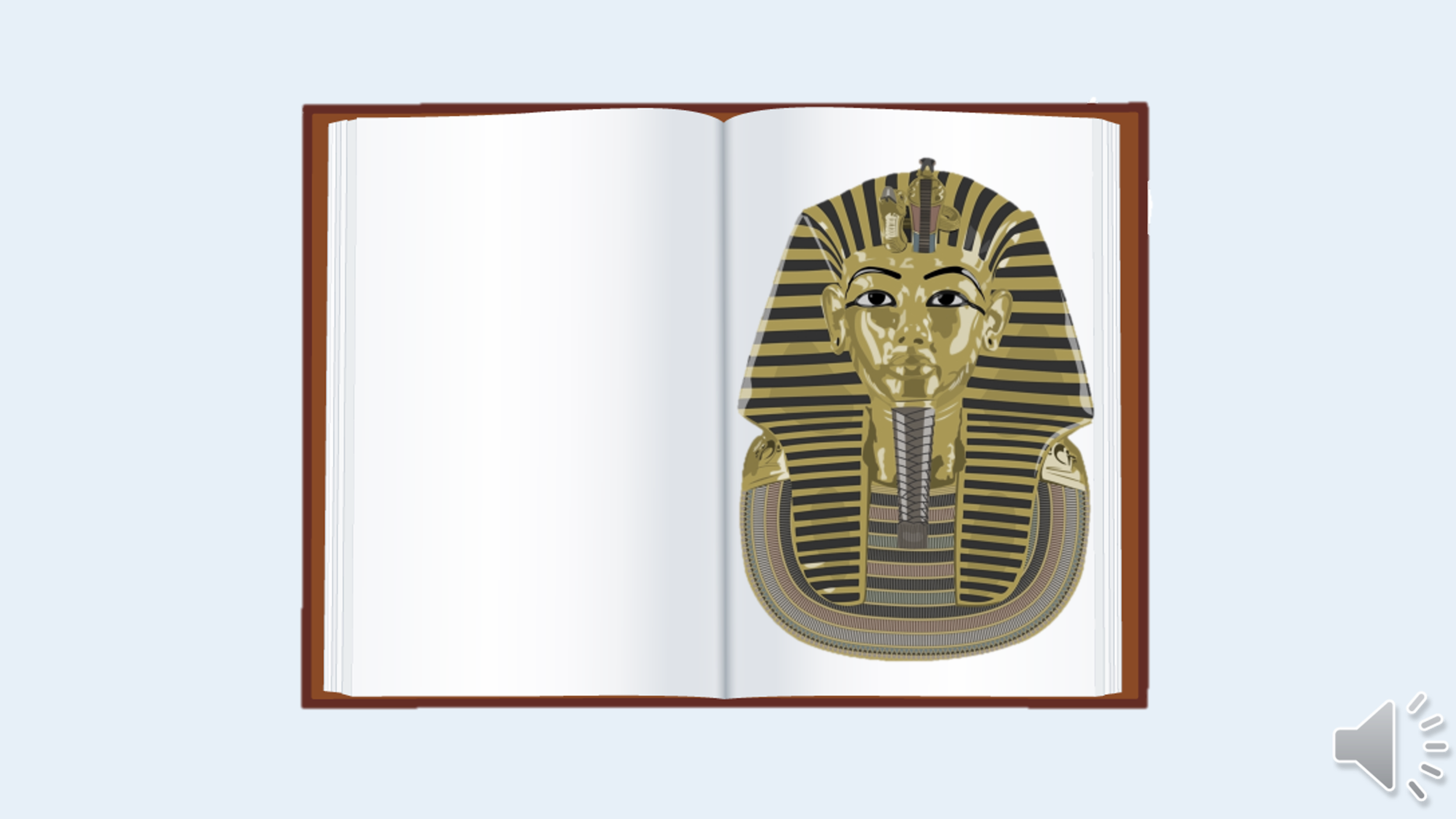
*Recording 5 of 8: 215 words lasting 80 s*

On very hot days in Egypt the temperature can reach 48 degrees Celsius. That is very hot! This is why it was so important for Pharaoh Olu to wear clothes that helped to keep their body feel cool. As it was so hot during the day, Pharaoh Olu also liked to eat refreshing snacks to keep cool. Pharaoh Olu liked to eat moon drop grapes. Grapes are mostly made up out of water, so they make a very refreshing snack. So, grapes helped to keep Pharaoh Olu from feeling too thirsty on the sunny days in ancient Egypt. The civilisation of ancient Egypt lasted for many, many years and because of this there were at least 170 different pharaohs who ruled in ancient Egypt at different times. For this reason, when Olu become a Pharaoh, Olu decided they wanted to leave behind a big legacy. What this means is that Pharaoh Olu really wanted people to remember their rule forever and ever. So, Olu thought a good way to do this would be to build a very big statue next to the pyramids. Olu sat on a fancy golden throne made just for the Pharaoh of ancient Egypt, Olu was being fanned with Olu’s favourite ostrich feather fan while Olu pondered what kind of statue they could build.

***Thought probe***


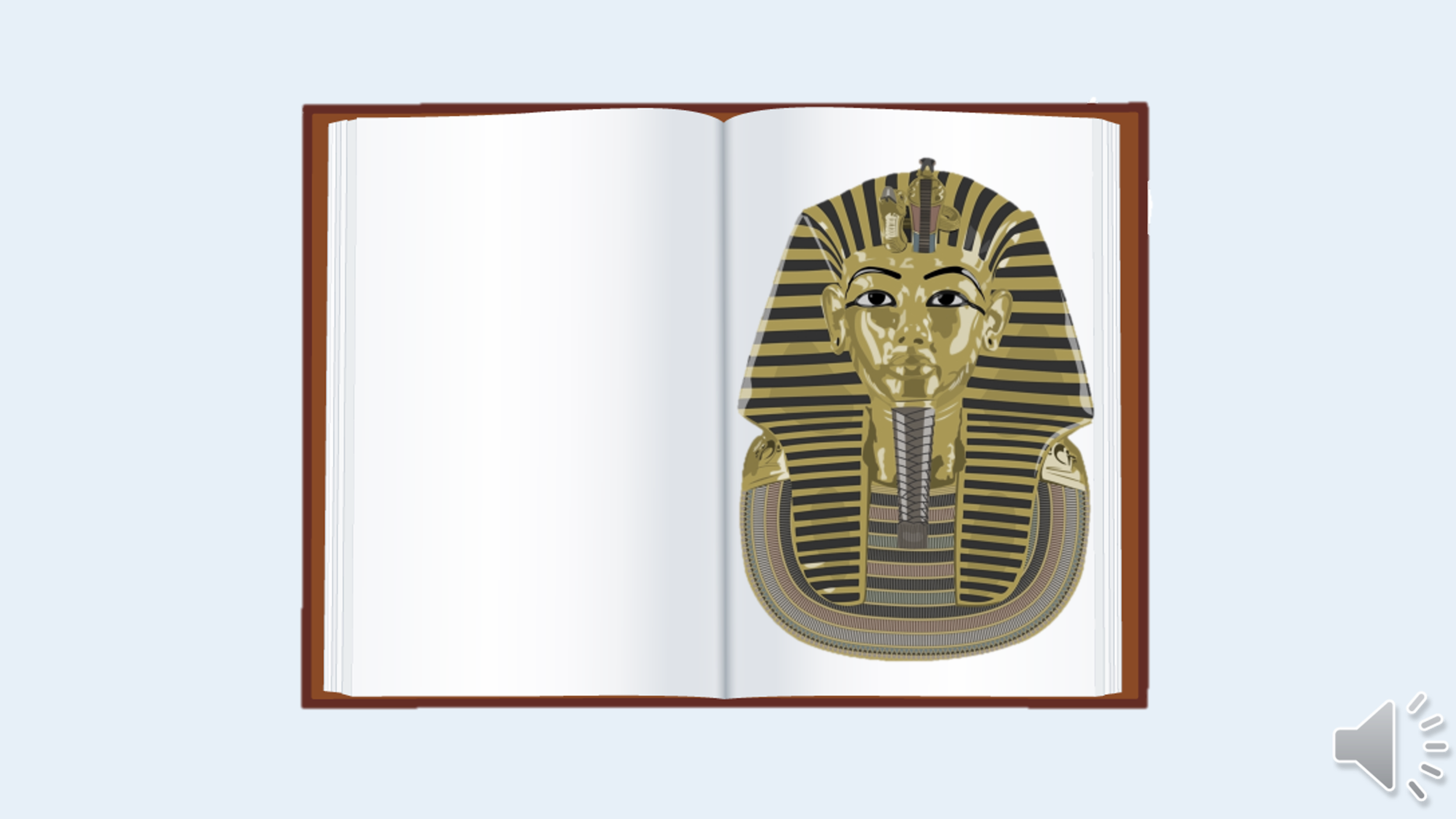
*Recording 6 of 8: 241 words lasting 98 s*

Pharaoh Olu was discussing the statue they wanted to build with a very important priestess of the time, she was called Neith. Pharaoh Olu and priestess Neith finally came to a decision; they decided the statue had to be big – gigantic and strong! This was so the statue could stand the test of time and people could come to see the statue for years and years to come.

Pharaoh Olu decided to get the workers of ancient Egypt to build the Bonx. The Bonx is a funny kind of statue because it has the body of a lion but the head of buffalo! This statue was very valuable to Pharaoh Olu and all the people of ancient Egypt. I will come back to this later but lots of different kinds of animals were very important to ancient Egyptian culture.
So, Pharaoh Olu built the Bonx and the statue is thousands of years old!  The statue is very, very old and it is actually now missing its nose! Oh no! How did this happen? Hm, well some people think it maybe got damaged a long time ago, maybe from a cannonball hit after being used for target practice. But I can tell you those people are wrong. I know what really happened to the Bonx’s nose! One very sunny day in Egypt, the Bonx was sunbathing when the statue felt a tickle in its nose…AA-CHOO. It was then that the Bonx sneezed a little too hard and the statue’s nose fell off!

***Thought probe***


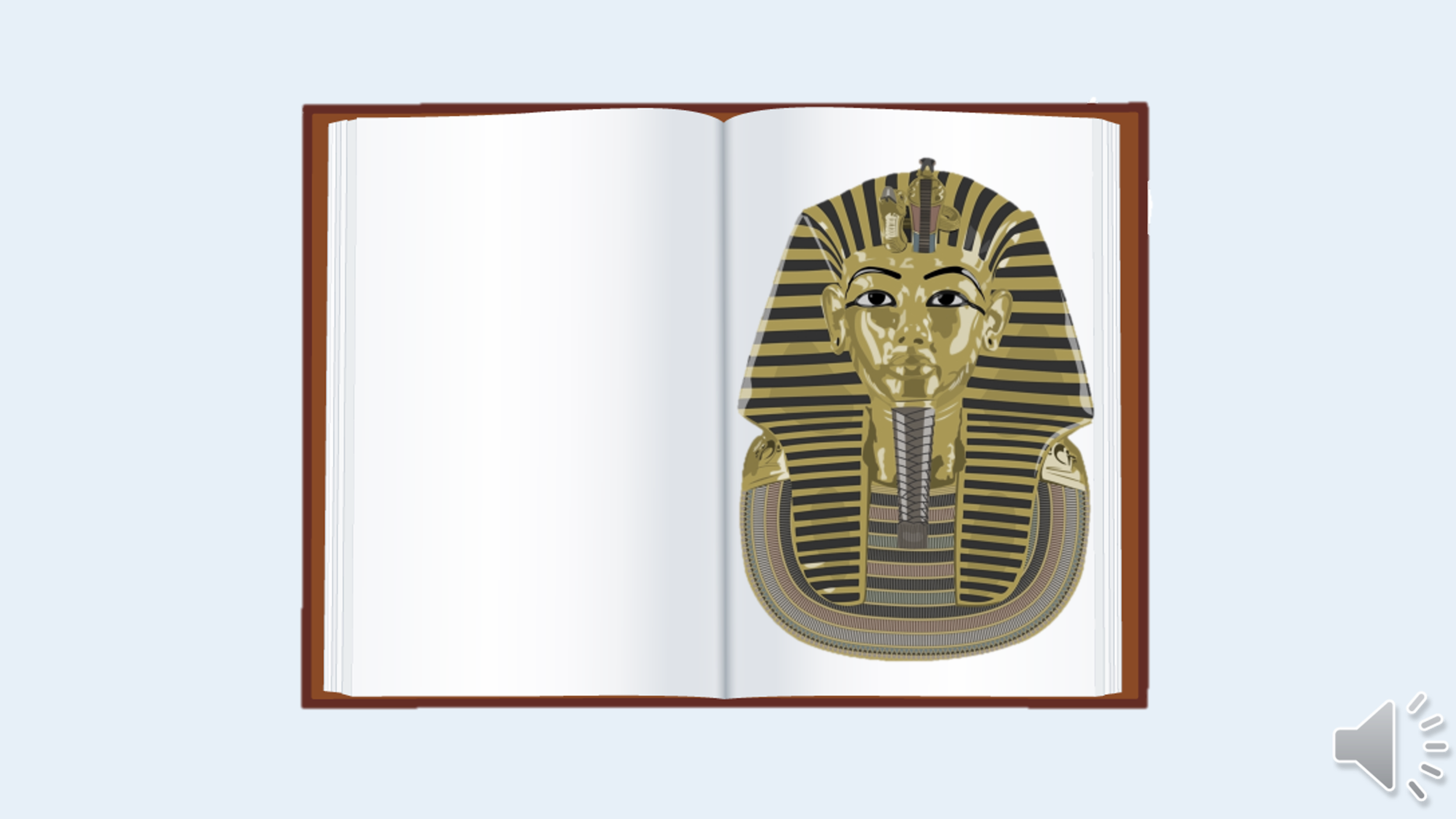
*Recording 7 of 8: 249 words lasting 105 s*

Oh no, the Bonx really misses its nose, and one thing is for certain, the Bonx would really like its nose back. So, if you happen to find the Bonx’s nose please do return it to the statue and bring some superglue to help them stick it back on! Once Pharaoh Olu’s workers had finished building the Bonx, Olu wondered what else could be done to help build their legacy? Pharaoh Olu really wanted to make sure people would remember them. Pharaoh Olu discussed the matter with an important Egyptian priest of the time, the priest was called Zosar. Pharaoh Olu and Zosar decided they would need to have the workers build a very impressive pyramid with a very magnificent tomb underneath that pyramid. Pharaoh Olu hired a lot of workers to build this pyramid. They decided to name the pyramid to be named ‘the Great Pyramid of Olufemi’. Underneath this pyramid, Pharaoh Olu decided to transform an underground cave into a cosy tomb. Pharaoh Olu had several requirements for this tomb, Olu wanted the tomb to be within a group of very important pyramids called ‘Valley of Pharaohs’. Pharaoh Olu wanted a very big tomb so that Olu’s golden throne and all Olu’s relics could fit inside it. Pharaoh Olu wanted this tomb to be very comfortable, and it is just as well because Olu would be left undisturbed until it was discovered in the year 2018. That means Pharaoh Olu was left alone in the tomb for over 3000 years, that is a very long time!

***Thought probe***


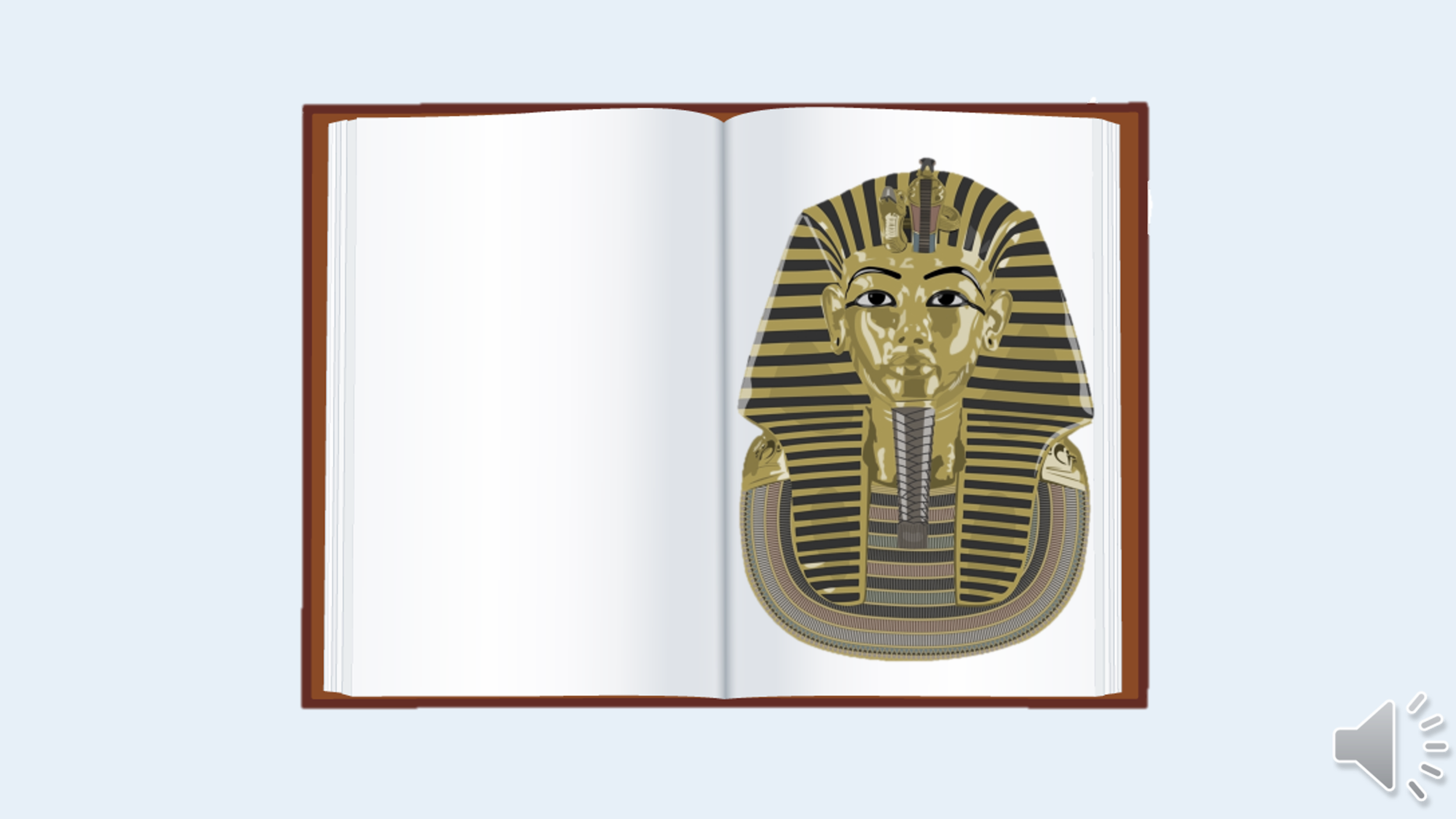
*Recording 8 of 8: 238 words lasting 98 s*

Sadly, Pharaoh Olu did die one day, after eating one too many prickly cucumbers. It was then that Olu was placed in this fancy tomb. When an important person had died in ancient Egypt the people would throw a big ceremony for that person. Lots of priests and priestesses would help to place the important person in their tomb. The very powerful priests and priestesses in ancient Egypt and liked to wear liked to wear purple robes and slippers.

Before the story ends, let’s talk about cats! Cats are one of the most popular pets in the world, cats have been kept as pets for thousands of years. There are lots of different types of cats, they can be different colours and sizes. Cats can have long hair, short hair and some have no hair at all! The people of Ancient Egypt believed cats to be sacred and holy. What this means is that in Ancient Egypt the people practically worshipped cats! Pharaoh Olu was no exception, when Olu was alive, Olu had two very special cats, they were called Fluffy and Sooty. They were both Siamese cats with striking orange eyes and cute little noses that were as black as coal. Pharaoh Olu loved these cats very much and treated them very well. They both had collars with bells, this meant Pharaoh Olu could always hear them coming as their bells made tinkling noises when they walked.

***Thought probe***

**5. T2 audio story transcript**

Mind wandering at time 2 was measured via intermittent thought probes embedded into an audio story about a dinosaur species. Please find below the transcript of the audio story alongside the screen presentation. When ‘***Thought probe***’ appears in the subsequent text it denotes that a thought probe occurred at this point. The following story is an original piece that was written for the purpose of this research.


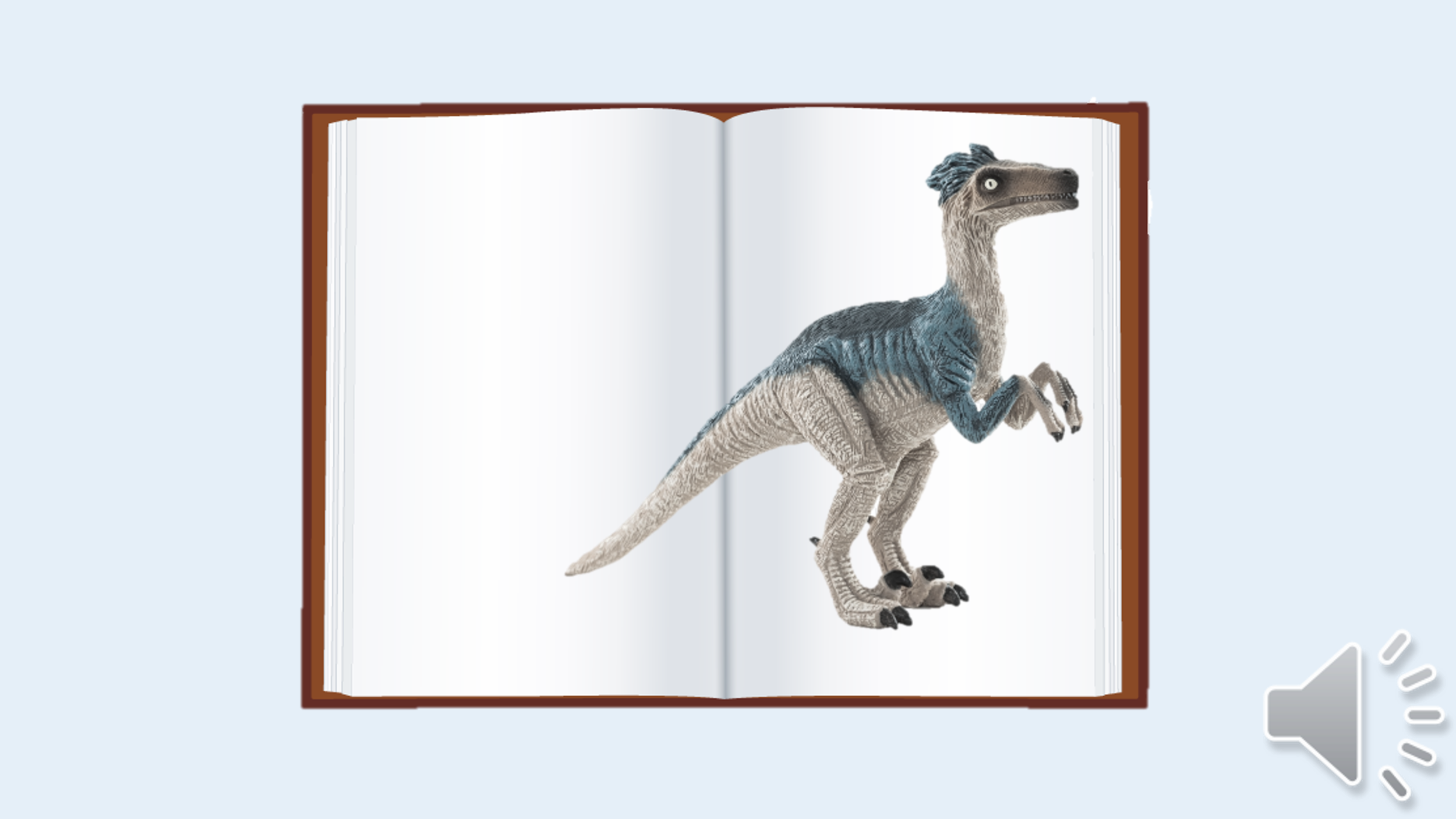
*Recording 1 of 8: 183 words lasting 65 s*

The dinosaurs ruled the earth for 165 million years. That is a really long time and for this reason people have split these millions of years into three different time periods with different names. First came the Triassic period and then came the Jurassic period and lastly came to Cretaceous period. All these big words are used to refer to different times when the dinosaurs roamed the earth. Today I am going to tell you a story about a dinosaur that lived 70 million years ago. This dinosaur, called Nikephorous, was alive during the Cretaceous period. Nikephorous is a very long name so let’s call this animal Nike for short. The name of the animal is very important because it means ‘carrying victory’ in a very old language called ancient Greek! How amazing is that? This animal was very victorious, and this is why it was given this very special name. Most dinosaur names are made up from old Greek words and either tell us something about the animal, or about where the animal lived or about the person who discovered the dinosaur!

***Thought probe (example below).***Note that all other probes will follow the same structure.


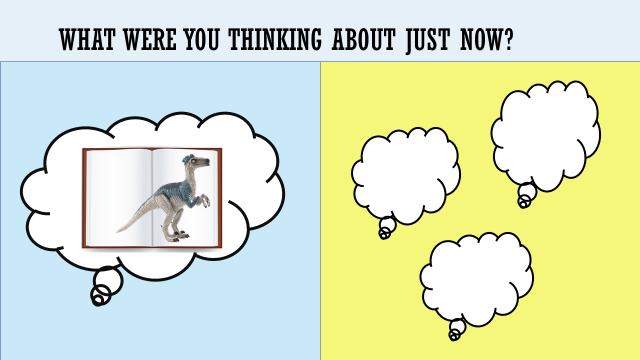


What were you thinking about just now? What was just said in the story or something different. Remember there are no right or wrong answers.


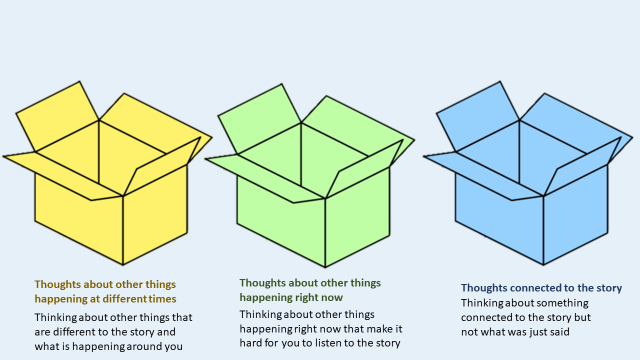
*(if ‘Something Different’)*

Can you tell me in which box does your thought belong? Is the thought connected to the story, is it a thought about other things happening at different times, or is it a thought about other things happening right now?


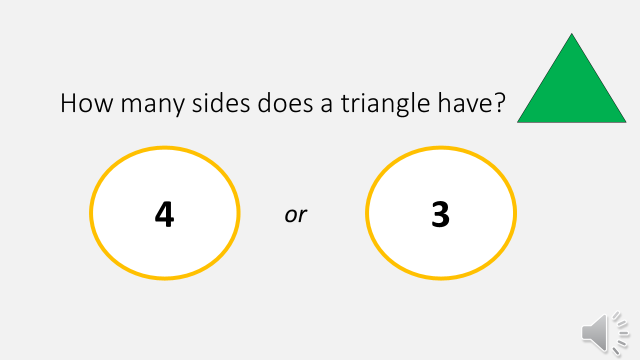


*(if ‘The Story’)*

 How many sides does a triangle have?

4 or 3?

**Listening Activity Transcript continued**


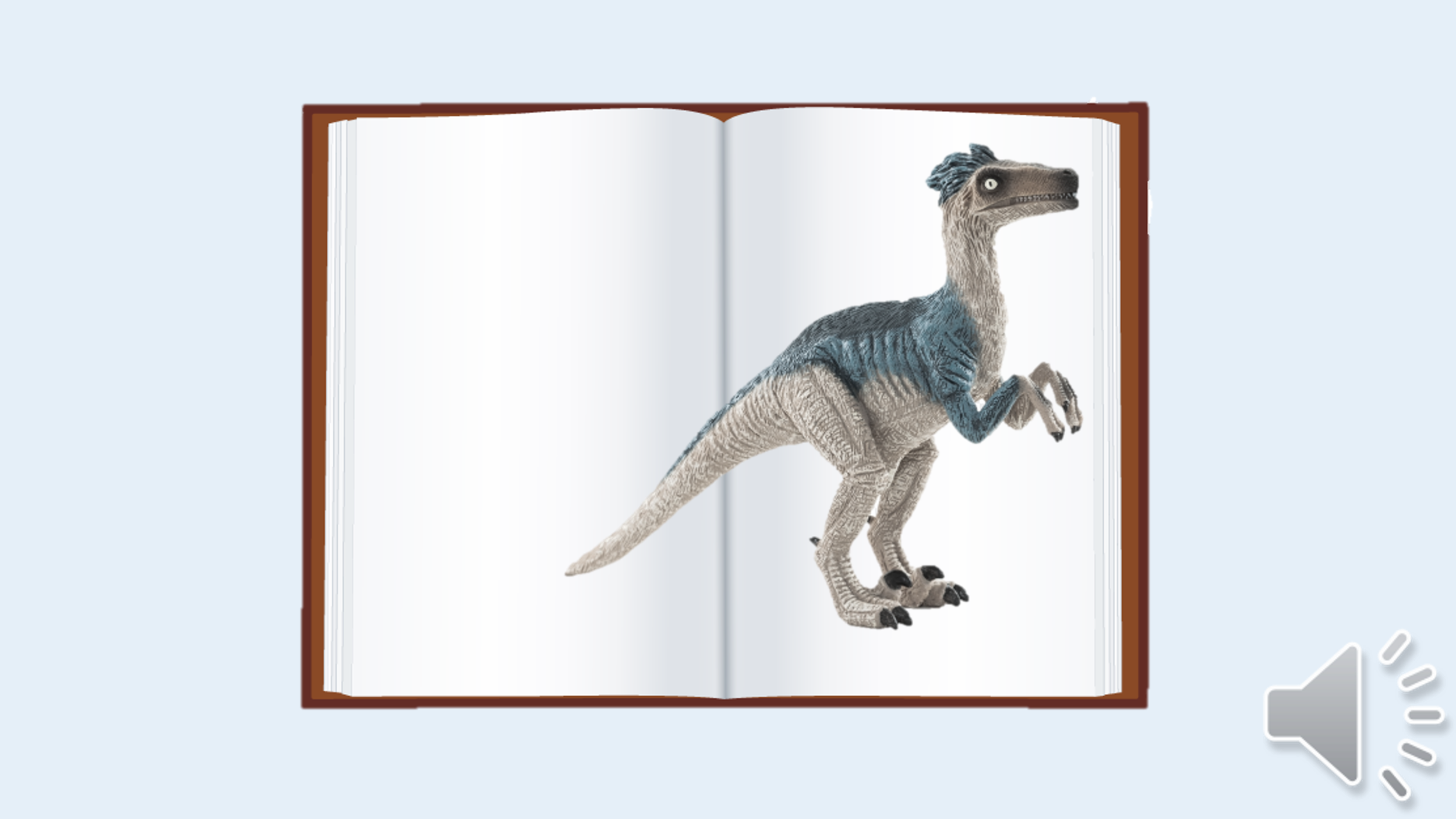
*Recording 2 of 8: 228 words lasting 85 s*

All the dinosaurs, including the Nike dinosaur, are now extinct. What this means is that there are no more dinosaurs that live anywhere on our world. There is a lot we don’t know about dinosaurs. This is probably because people and dinosaurs didn’t live at the same time and so we have no written records about the dinosaurs from those millions and millions of years ago! Although, we can learn all about dinosaurs from things called fossils. Fossils are very special rocks which give us clues about the past by giving us ideas about animals and plants that lived a long time ago. Have you heard of palaeontologists? That is a big word to describe scientists who are interested in fossils! Fossils can give us an idea of how different dinosaurs might have looked by protecting dinosaur bones, teeth, their footprints and even their eggs! From fossils we know that dinosaurs came in lots of different shapes and sizes! Some dinosaurs were very, very, very big while other dinosaurs were very, very, very tiny. One of the smallest dinosaurs we know about is called Compsognathus, this dinosaur was about the same size as a chicken! A Nike dinosaur could grow to be about 2 metres tall. That's smaller than most basketball players. A very big dinosaur was the diplodocus, this dinosaur was as long as a football pitch!

***Thought probe***


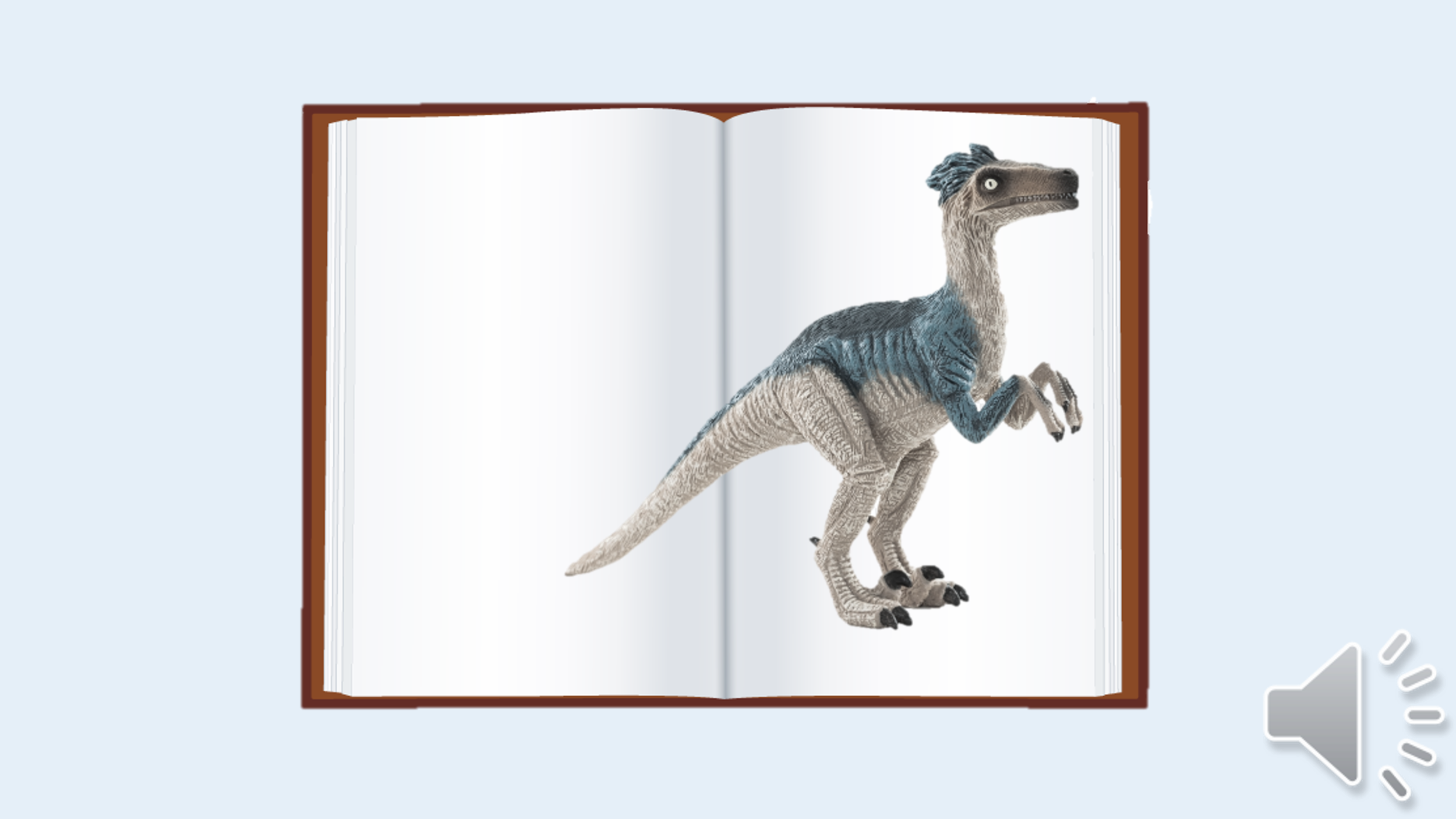
*Recording 3 of 8: 181 words lasting 75 s*

So where could we find Nike dinosaurs? These dinosaurs lived in the Amazon rainforest. They liked to live beside the edge of the Amazon River. You might have heard about the Amazon River, it is the biggest river in the world, what this means is that it has the most water in it compared to any other river. The Amazon River & Rainforest is in South America which is one of the world’s seven continents. All the Nike dinosaurs really liked to live here! Nike dinosaurs were herbivores, which means they liked to eat plants, vegetables, and fruits. There were lots of plants for the Nike dinosaurs to eat in the Amazon rainforest! Nike dinosaurs loved to eat mango; a fruit which grows easily in the heat of the Amazon rainforest! The Nike dinosaurs loved to live in the forest as the trees made a good hiding place if there were big dinosaurs out and about. The Amazon rainforest is a truly amazing place, so many kinds of animals live there and lots of different kinds of foods can grow there.

***Thought probe***


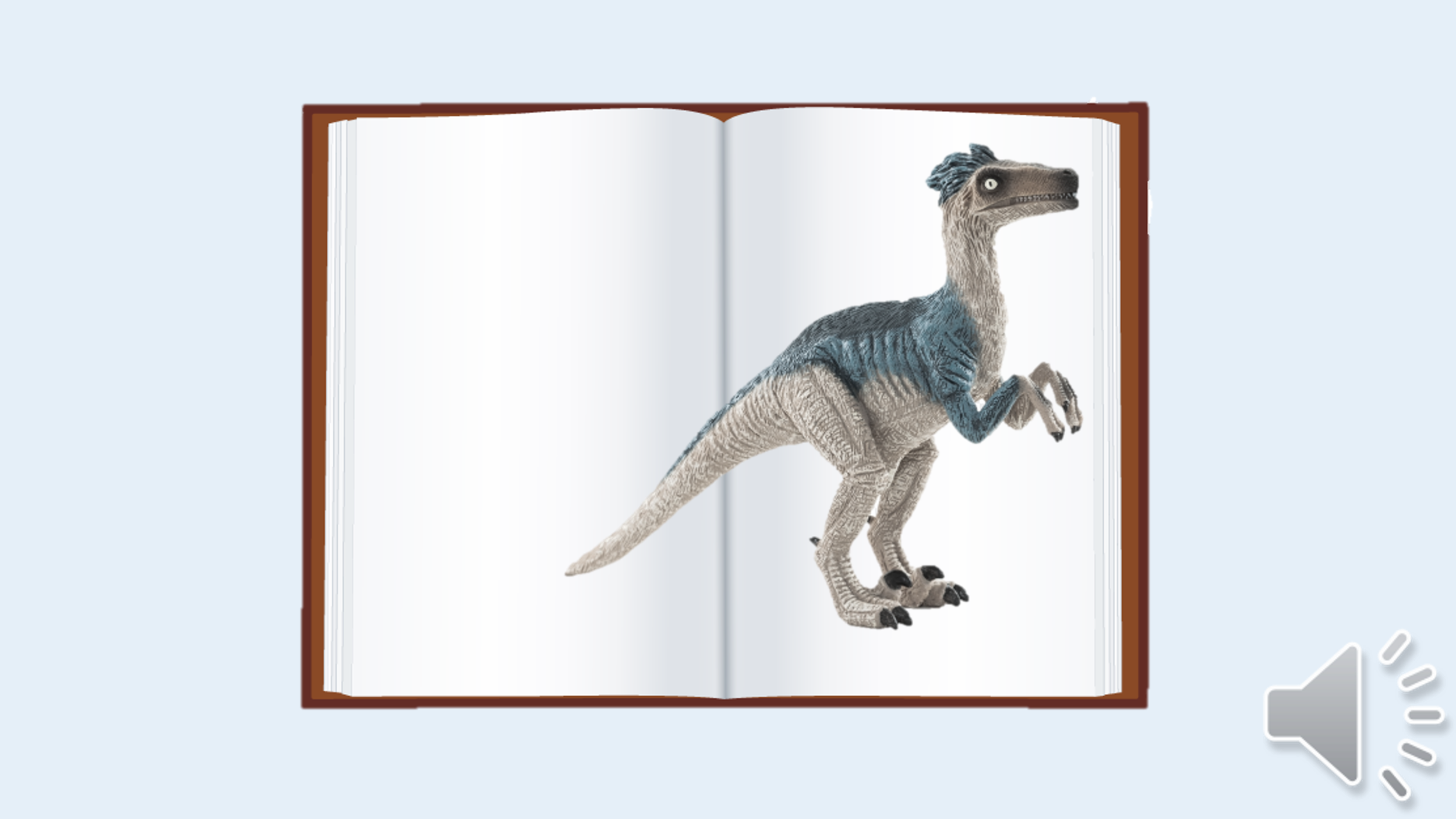
*Recording 4 of 8: 232 words lasting 90 s*

The skeletons of dinosaurs have been found all over the world. Even in Northern Ireland, how amazing is that? Fossilised bones of two different types of dinosaurs have been found in Co. Antrim. Fossilised eggs from the dinosaurs have also been found. Discovering a dinosaur egg is a very special find for a palaeontologist (if you remember they are the scientists who are really interested in dinosaurs). Dinosaur eggs have taught us a lot about how baby dinosaurs were born. As far as we know, all dinosaurs that lived were oviparous. That is a fancy word to say we think all dinosaurs laid eggs. We think that a female dinosaur could lay up to 20 eggs at a time! That is an awful lot of eggs. Dinosaurs would build special nests to keep their eggs safe and warm. Just like birds and reptiles do today. Dinosaurs used different materials to build their nests, some used soil, and others used leaves from plants. Some dinosaurs even dug holes in the ground to lay their eggs in! Like all other dinosaurs that we know of so far, the Nike dinosaurs also laid eggs. Their eggs were oval shaped and covered in yellow spots. The Nike dinosaur could lay up to five eggs at a time. Their egg nests were made from banana leaves and located close to the Amazon River within the Amazon rainforest.

***Thought probe***


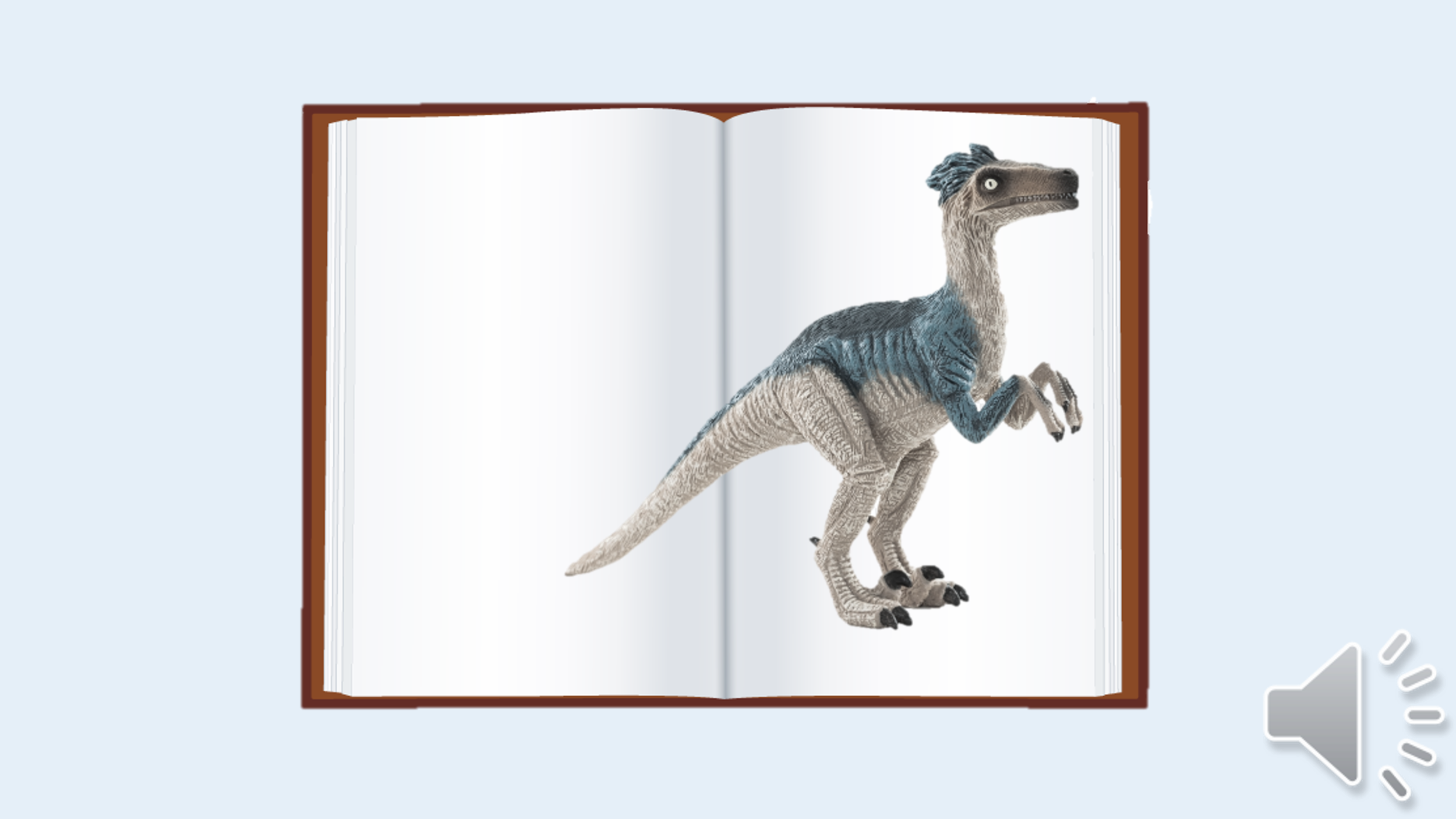
*Recording 5 of 8: 160 words lasting 75 s*

How do we know what dinosaurs looked like? Fossils can tell scientists what dinosaur skin was like. What this means, we can tell if a dinosaur was covered in scales, pebbles or if they were covered in feathers! Scientists have been studying the fossils of the Nike dinosaur very, very, very carefully and they discovered this dinosaur was covered in feathers.

One thing the fossils can't tell us is what colour the dinosaurs were, so we don’t know if dinosaurs were orange, green, blue or even pink. Scientists have to take a really smart guess at what colours the dinosaurs might have been…some scientists base their smart guesses on what certain animals alive today look like – such as elephants, rhinoceroses, crocodiles. These scientists think that dinosaur skins were probably dull shades of grey, green and blue. Although, some scientists do think the opposite might be true and the dinosaurs could have been orange, green maybe with yellow and purple spots!

***Thought probe***


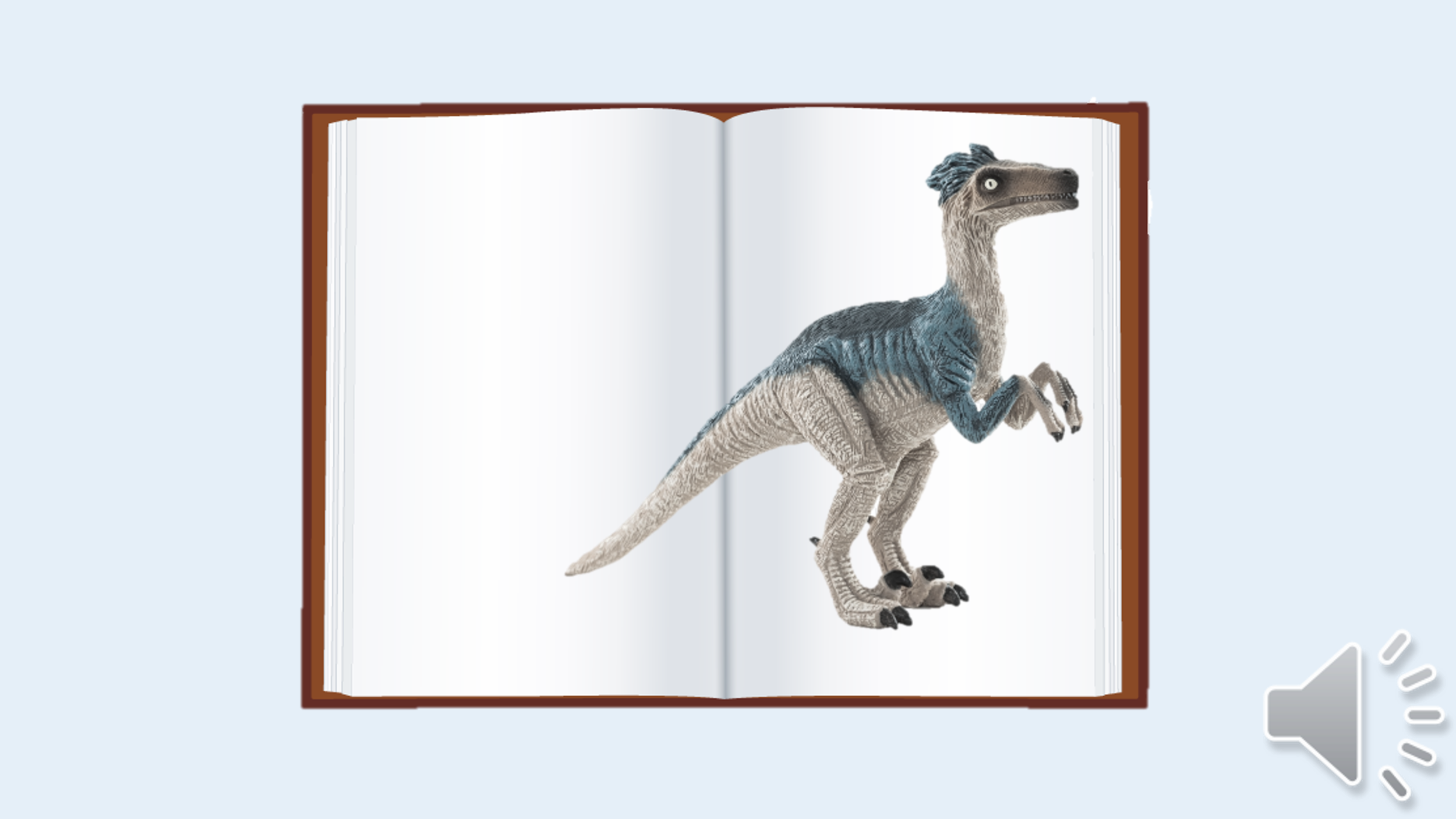
*Recording 6 of 8: 175 words lasting 75 s*

The bones of 700 different types of dinosaurs have been found. How amazing is that? So, we know there were at least 700 types of dinosaurs that roamed the earth at one point. Scientists can sometimes sort the dinosaurs into different groups, sometimes they do this by that the dinosaurs ate, when they lived and sometimes by their size. Some dinosaurs, like tyrannosaurus rex were carnivores - meaning they only ate meat. Some dinosaurs, like Nike, were herbivores – meaning they only ate plants. Other dinosaurs were omnivores like velociraptors – which means they ate both meat and plants. Each dinosaur was different and special in its own way. The Nike was a smart dinosaur, scientists think this dinosaur was so successful at keeping safe from bigger dinosaurs by hiding in the trees of the Amazon rainforest. The Stegosaurus is a well-known dinosaur from the Jurassic period. This dinosaur had impressive plates all the way down its back, it was as big as an elephant, but its brain was only the size of a ping pong ball!

***Thought probe***


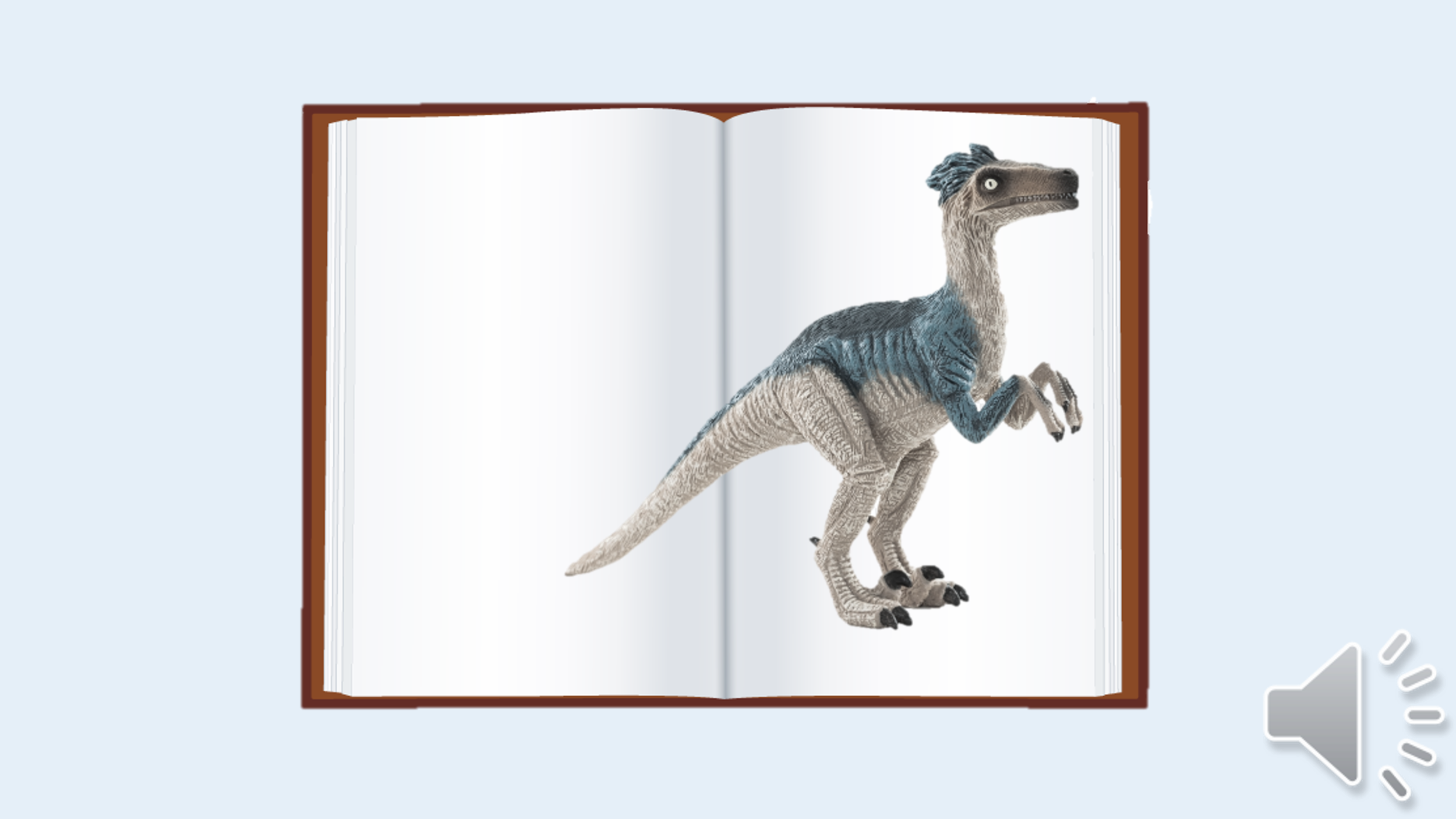
*Recording 7 of 8: 180 words lasting 70 s*

The tyrannosaurus rex was one of the biggest meat eaters, it lived at the very end of the era of the dinosaurs. T-Rex was a fierce dinosaur with teeth the size of bananas, they walked on two legs and could run at speeds up to 20 mph. No one knows for sure what happened to the dinosaurs. They ruled the earth for millions and millions of years but suddenly disappeared about 66 million years ago. It has long been believed that a massive asteroid hit the earth 66 million years ago and this is what wiped out the dinosaurs! An asteroid is a giant rock that comes from space, scientists think when this hit the earth it caused lots of volcanic eruptions, wildfires and tons of toxic gases. Although, scientists think that the Nike dinosaurs became extinct long before the asteroid. From looking very closely at the special fossil rocks scientists think the last Nike dinosaur was alive about 70 million years ago. No one really knows what caused the Nike dinosaurs to go extinct those many million years ago.

***Thought probe***


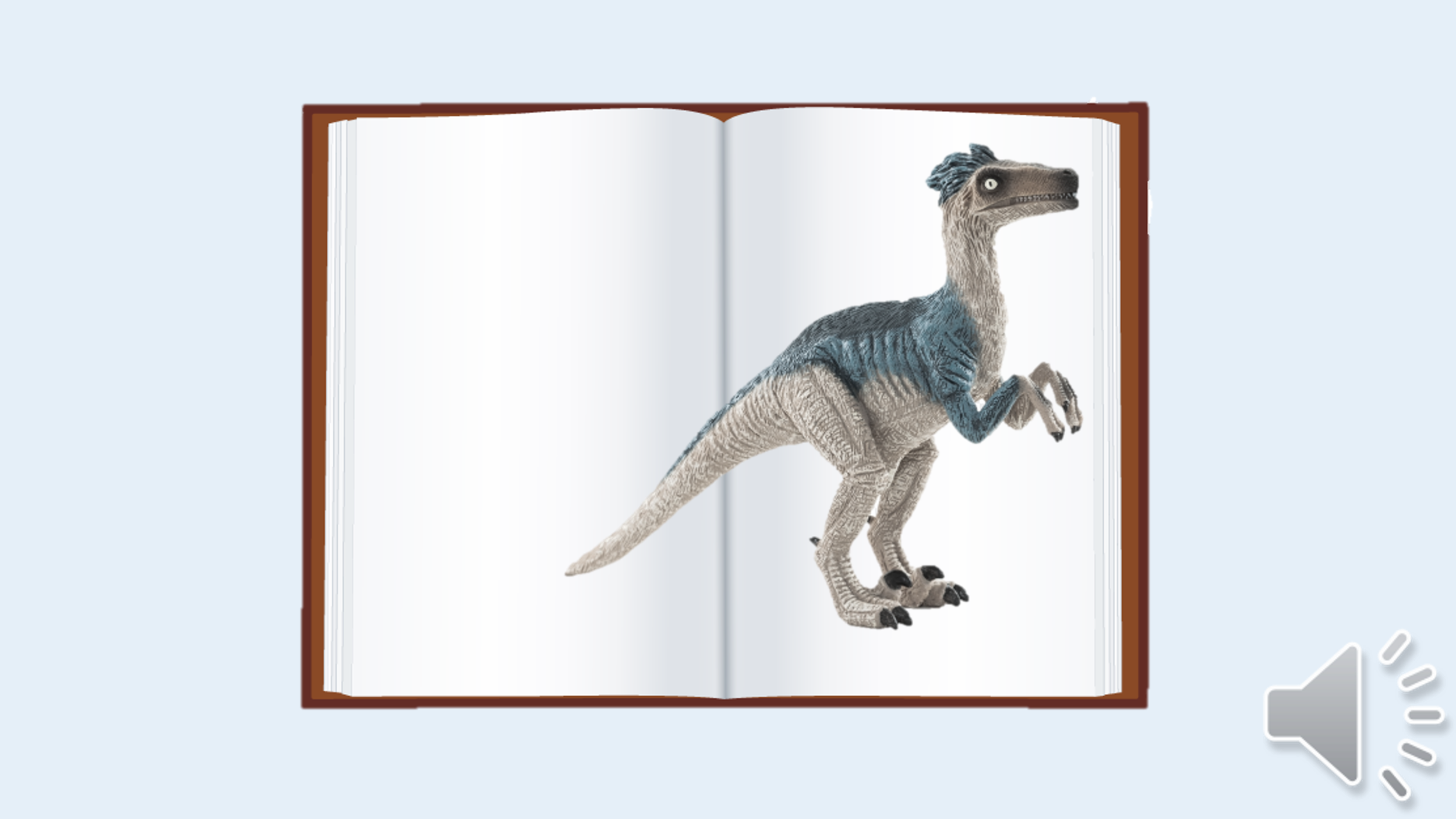
*Recording 8 of 8: 178 words lasting 70 s*

Different animals, not just the dinosaurs, can become extinct. Have you heard about the woolly mammoth? It was a very big animal with very long tusks that became extinct several thousand years ago. More recently the animal called the dodo became extinct a few hundred years ago. The dodo was a bird who couldn’t fly and came from a country called Mauritius. Before an animal becomes extinct, scientists will name these animals as endangered. What this means is that animal is very close to becoming extinct. There are several endangered animals today, certain types of elephants, leopards and rhinos are endangered. People can help endangered animals by taking care of our planet. This is a reason why it is important for people to recycle and reuse certain items. I always like to reuse glass jars. Recycling means we can take certain things and use them again. Instead of putting a plastic bottle or cardboard into the black bin you can put it into your recycling bin and the bin collectors will take these things to a recycling centre.

**8. Memory recall tests**

To measure initial memory recall of the audio story a 10-item multiple choice test was completed shortly after listening to the audio stories at both time 1 (T1) and time 2 (T2). Delayed memory retention of the audio story presented at T1 was completed after a seven-day delay at T2. The presentation of T1 Set A and Set B tests were counterbalanced, 50% of participants completed Set A at T1 and Set B at T2 and the other 50% of participants completed Set B at T1 and Set A at T2. All test questions were derived from the novel components of the story. The image below illustrates how the question was presented on the screen for participants. Please note, all questions and possible answers were read aloud by the researcher.

**T1 Set A test**


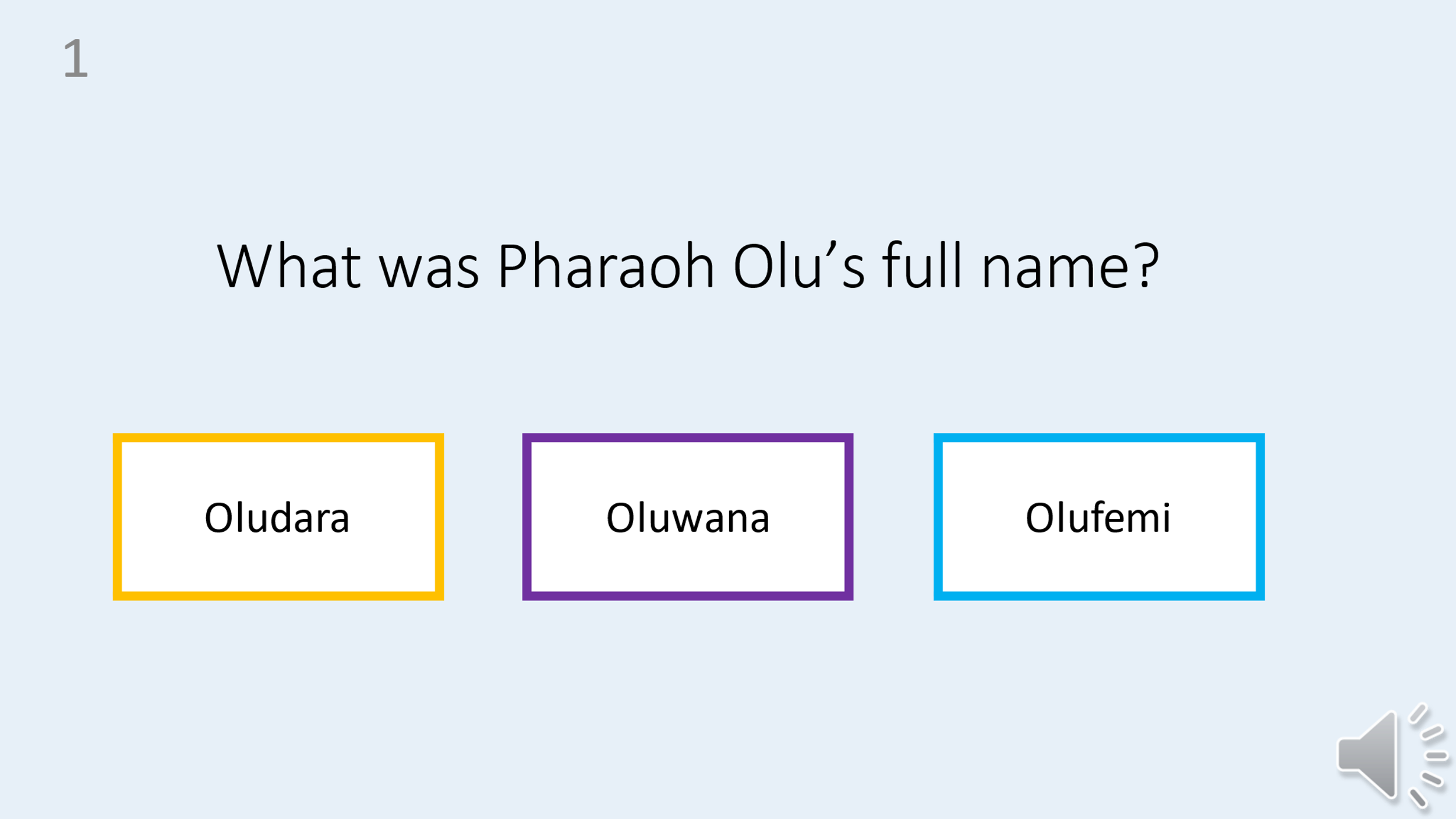


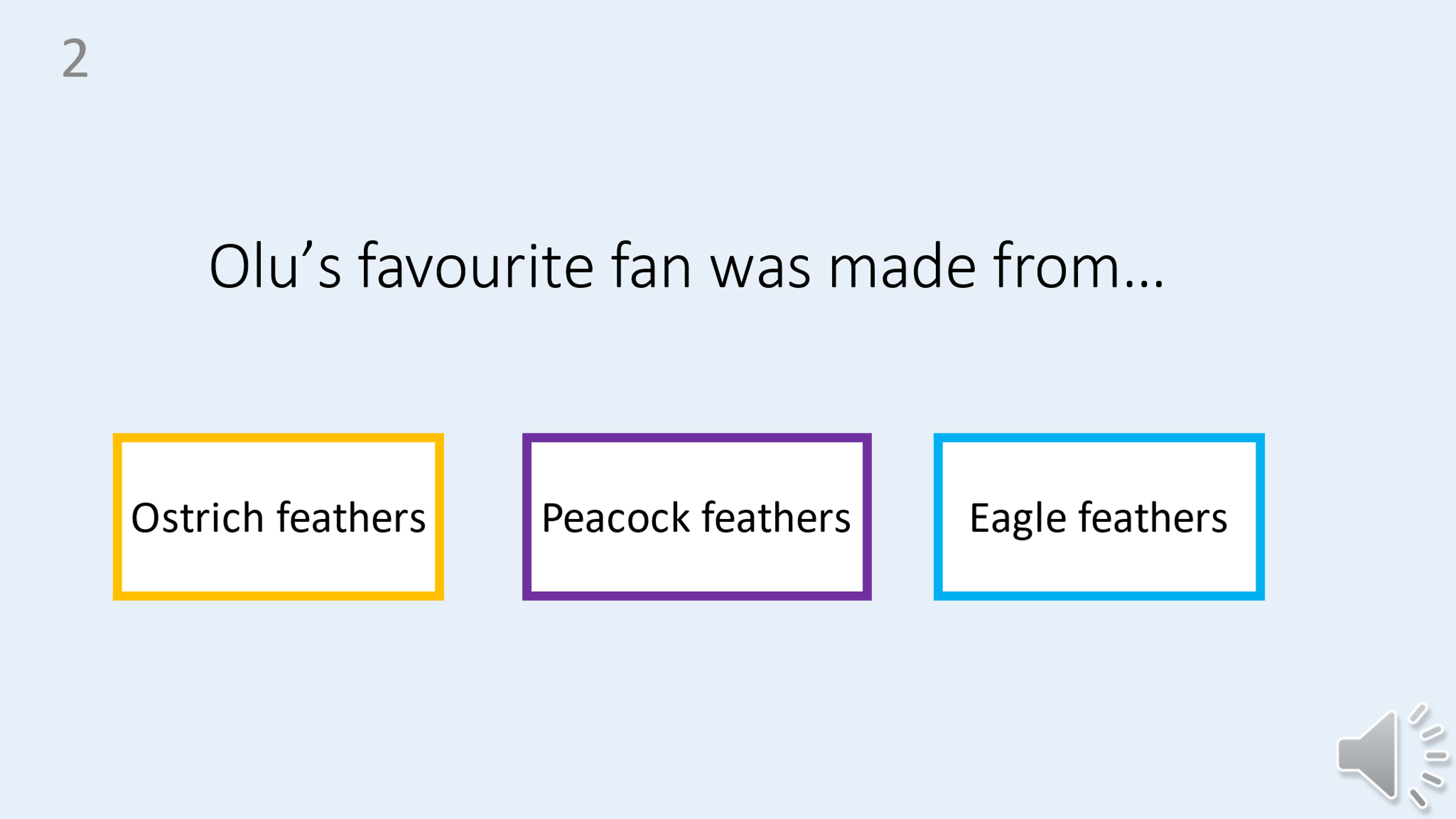


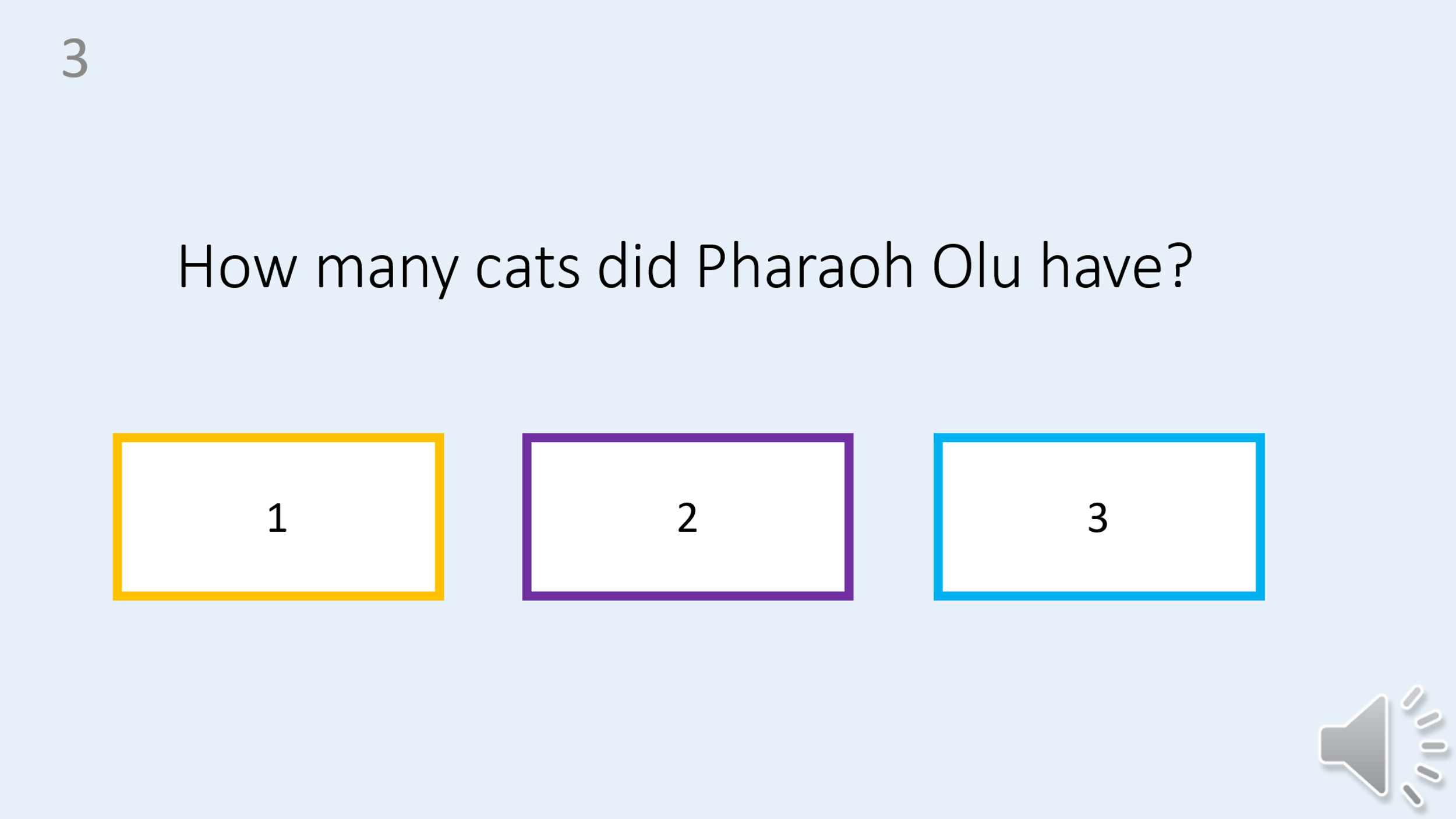


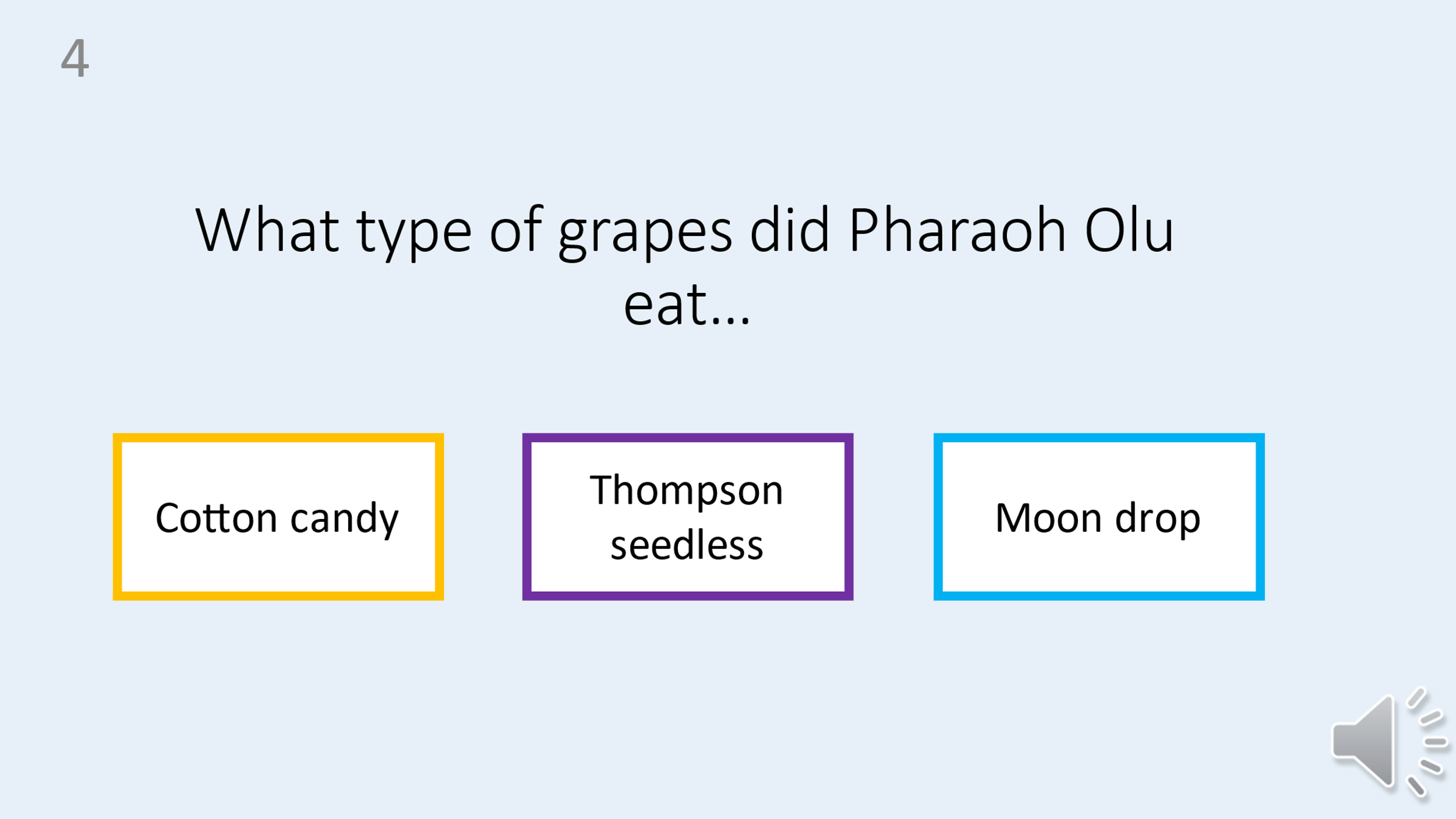


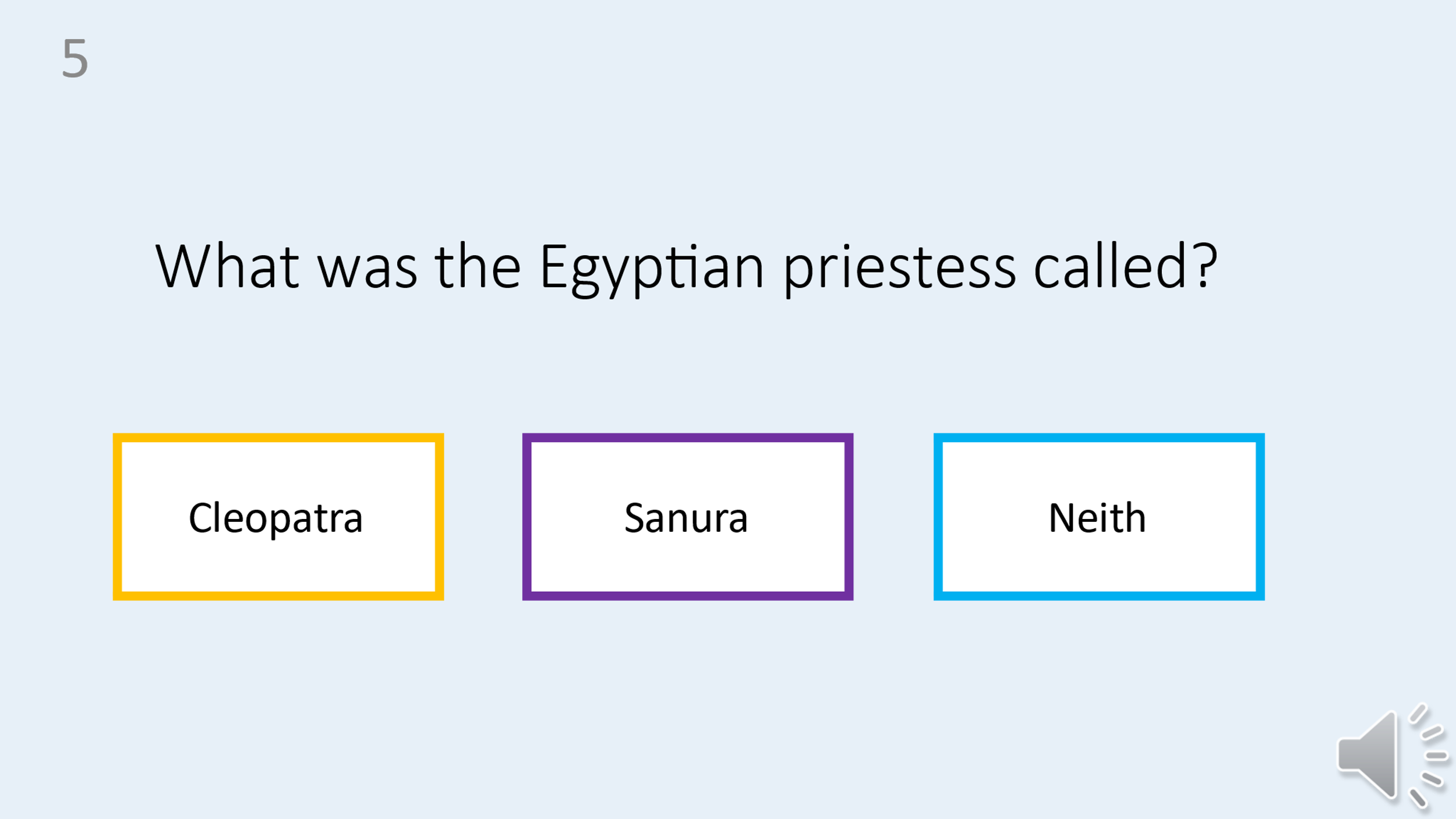


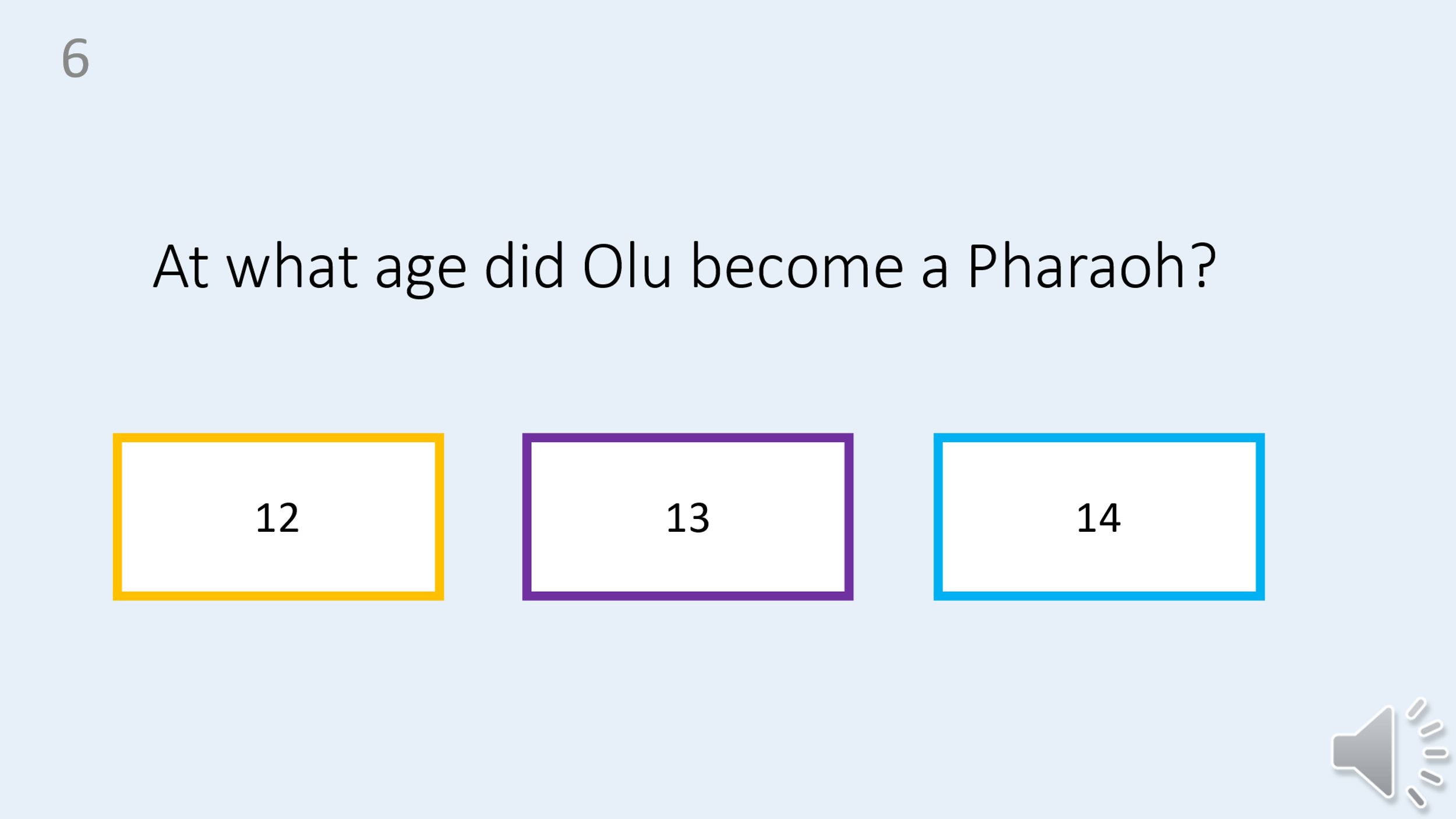


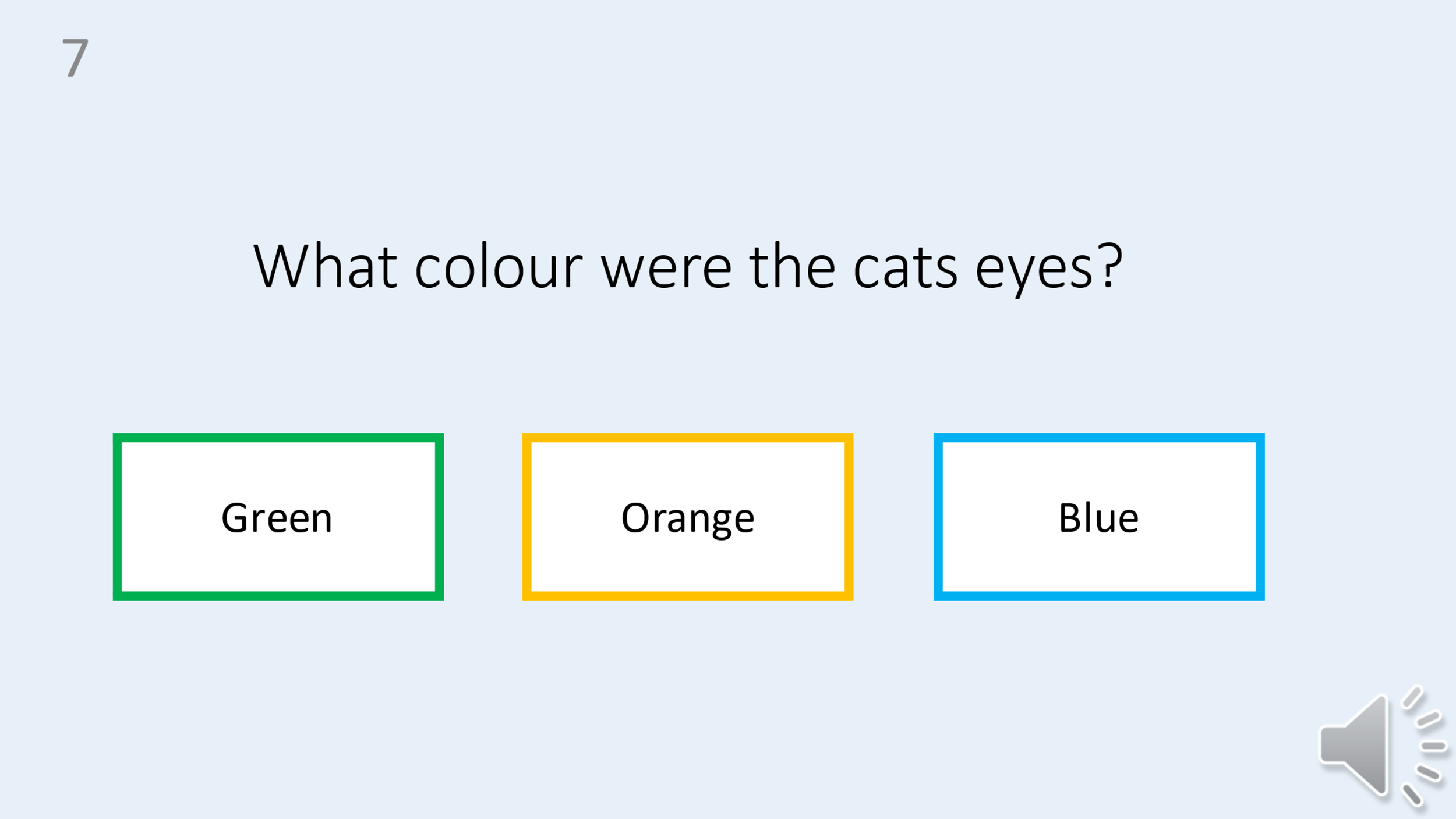


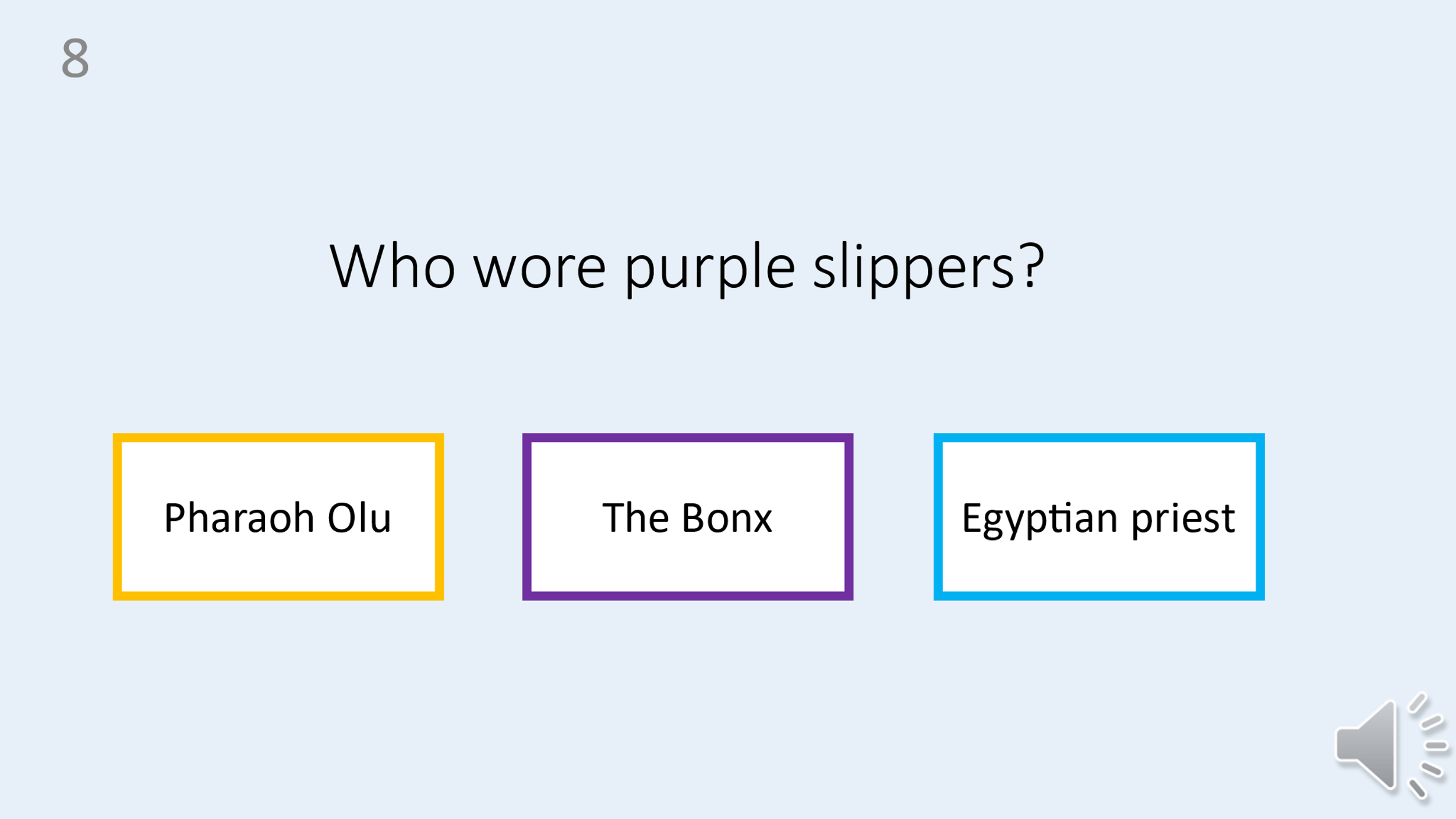


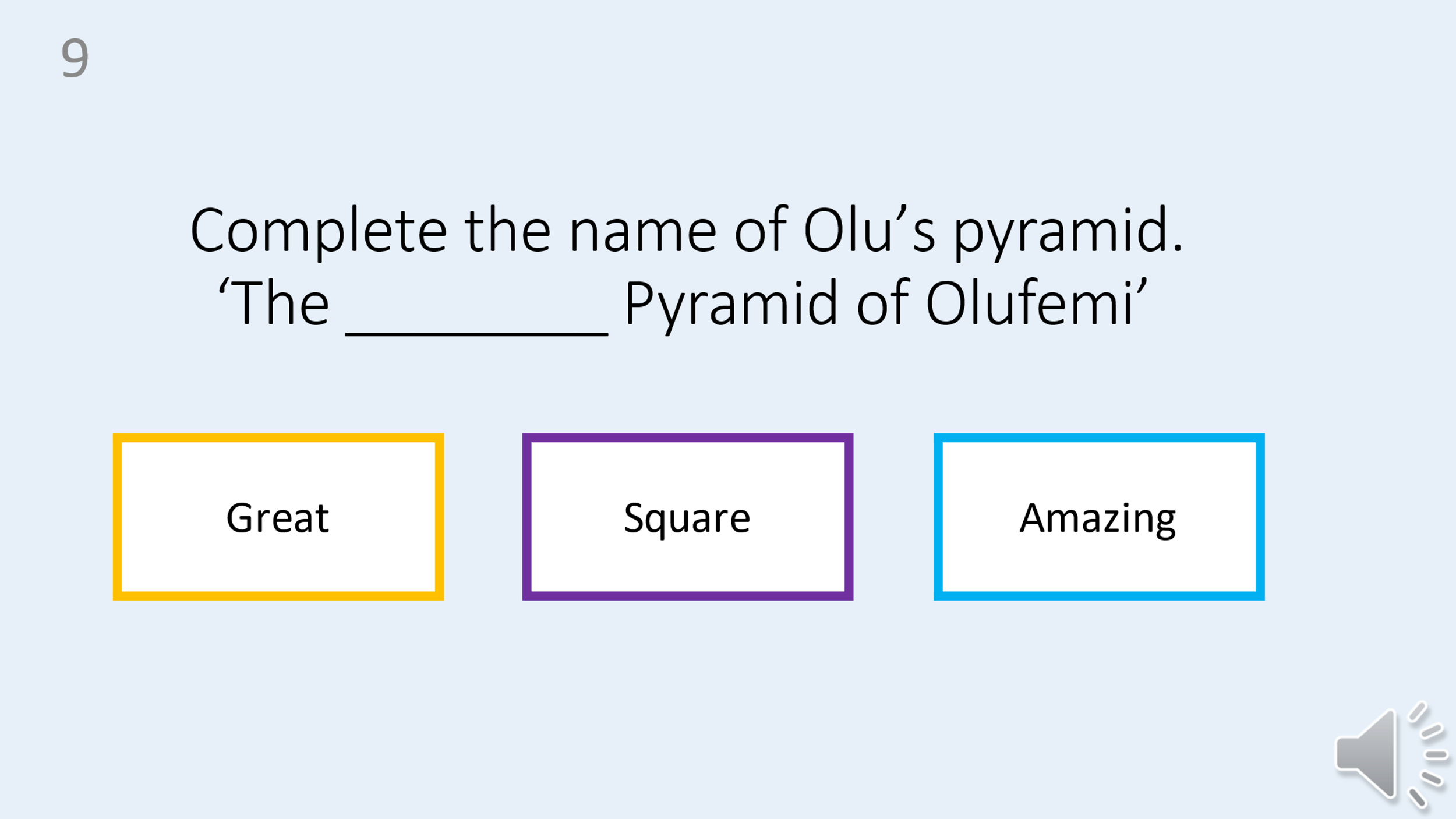


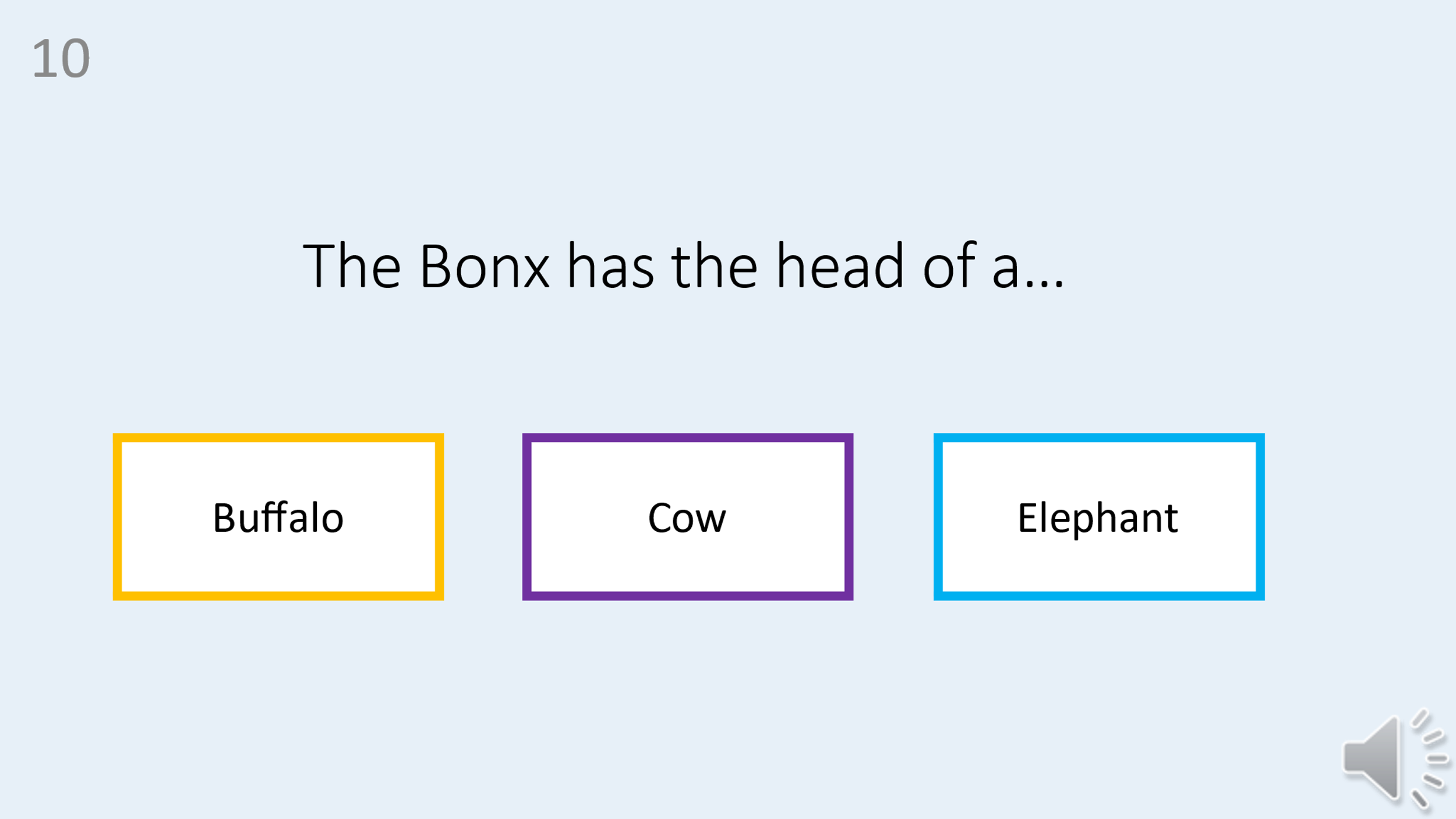


**T1 Set B test**


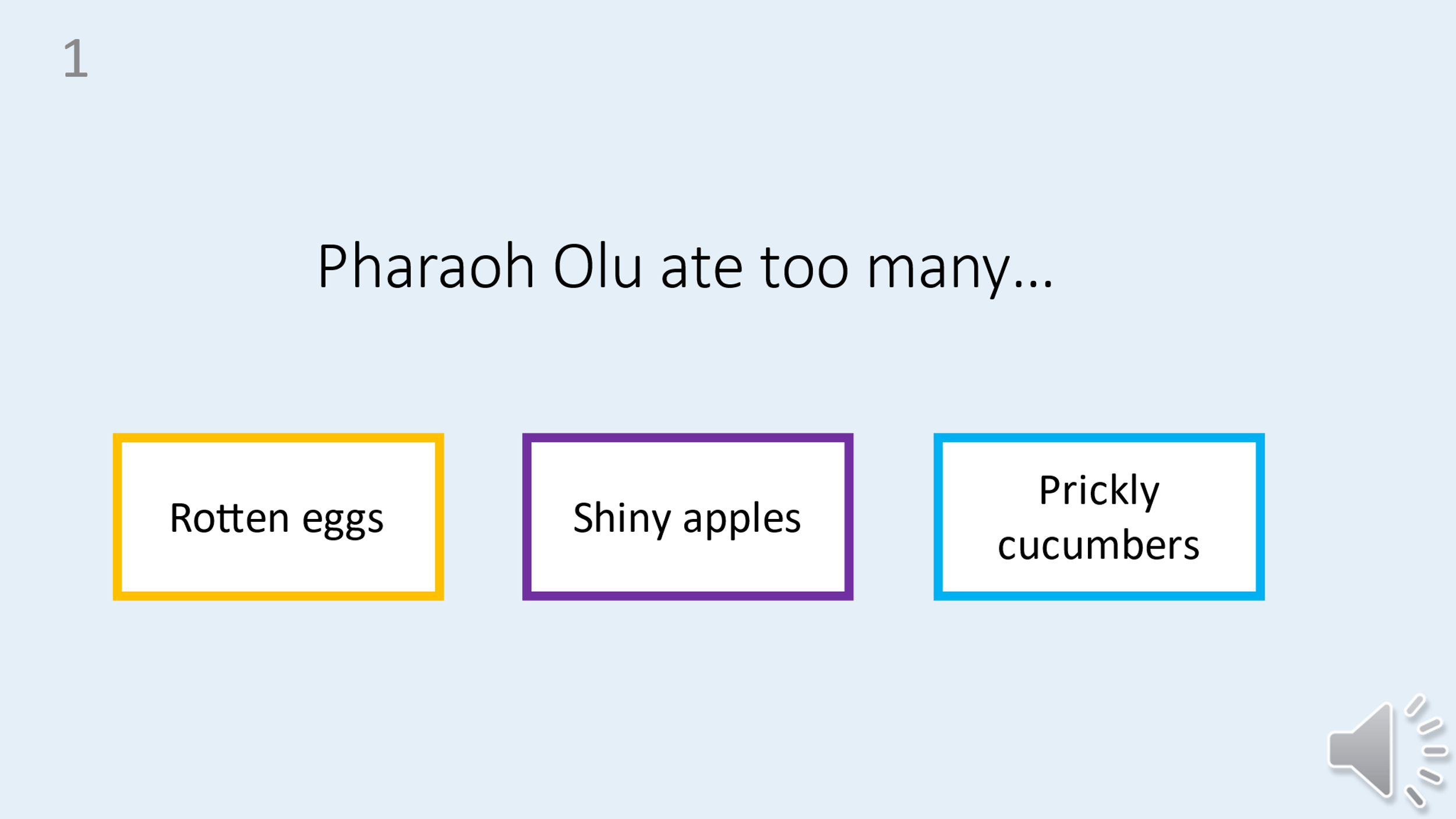


**T2 test**
